# Supplementary material for: Human ZBP1 induces cell death‐independent inflammatory signaling via RIPK3 and RIPK1
Source: EMBO Rep. 2022 Oct 21;23(12):e55839. doi: 10.15252/embr.202255839 (PMC9724671; doi:10.15252/embr.202255839)
Supplement: Supplementary file 1 — Expanded View Figures PDF [file EMBR-23-e55839-s001.pdf]

Expanded View Figures

Figure EV1. ZBP1 induces inflammatory signaling independently of cell death.

A Western blot analysis of ZBP1 levels in HT29, HT29/TO-ZBP1^{WT}, and HT29/TO-ZBP1$^{Z\alpha1\alpha2mut}$ cells treated or not with Dox or IFNβ (10 ng/ml) for 24 h. Blots are representative of two biological replicates.

B Cell death analysis of HT29 and HT29/TO-ZBP1 cells as indicated following 48 h treatment with Dox using SytoxGreen to stain dead cells. Data are presented as mean ± S.E.M ($n = 5$ biological replicates). One-way ANOVA and Sidak's multiple comparisons tests were used to test for the statistical differences between indicated conditions and untreated condition of the same cell line. *$P = 0.0283$; ****$P < 0.0001$.

C Relative viability of HT29/TO-ZBP1^{WT} and HT29/TO-ZBP1$^{Z\alpha1\alpha2mut}$ cells treated with Dox for up to 72 h was determined by the CellTitre-Glo assay. Values were normalized to that of 0 h for each cell line. Data are presented as mean with S.E.M ($n = 3$ biological replicates). Two-way ANOVA and Tukey's multiple comparisons tests were used to test for the statistical differences between different cell lines. n.s. = not significant ($P = 0.9510$); ****$P < 0.0001$.

D Cell death analysis of HT29/TO-ZBP1^{WT} cells by SytoxGreen staining following treatment with TSZ in combination with the indicated inhibitors. Cells were pretreated with 100 nM LCL161, 20 μM zVAD combined with10 μM GSK'840, 10 μM GSK'872, or 10 μM Nec1s for 1 h followed by treatment with 10 ng/ml TNF. Cell death analysis was carried out at the end of 24 h incubation with all compounds. Data are presented as mean ($n = 2$ biological replicates) with individual data points indicated.

E, F Cytokine concentrations in the culture media of HT29/TO-ZBP1 cells treated with Dox for 24 h. Cells from the same wells were lysed for Western blot to determine ZBP1 expression levels. (E) Data are presented as mean with S.E.M ($n = 6$ biological replicates). Brown-Forsythe and Welch ANOVA tests and Dunnet's T3 multiple comparisons test were used to test for statistical significances between indicated conditions. ***$P = 0.0002$; *$P = 0.0447$; ****$P < 0.0001$; n.s., not significant ($P = 0.2153$). Western blots are representative of six biological replicates. (F) Data are presented as mean with S.E.M ($n = 4$ biological replicates). Unpaired t-tests were used to test for the statistical differences between the indicated conditions. *$P = 0.0257$; **$P = 0.0034$. Cell lysates from one biological replicate were analyzed by Western blotting.

G CXCL1 concentration in the culture media from the experiment described in (Fig 1E). Data are presented as mean with S.E.M. ($n = 3$ biological replicates). Two-way ANOVA tests and Sidak's multiple comparisons test were used to test for statistical differences between indicated conditions. n.s., not significant ($P > 0.09$); ****$P < 0.0001$.

H CXCL8 concentration in the culture media of HT29/TO-ZBP1^{WT} cells treated with 0 or 50 ng/ml Dox in combination with DMSO, 10 μM Nec1s, 10 μM GSK'872, or 1 μM NSA for 24 h. Data are plotted as mean with S.E.M. ($n = 4$ biological replicates). One-way ANOVA and Sidak's multiple comparisons tests were used to test for statistical differences between indicated conditions. n.s. = not significant ($P > 0.39$); **$P = 0.0012$; ****$P < 0.0001$; *$P = 0.0297$. Cells from the same wells were analyzed by Western blotting for ZBP1 and RIPK3 levels. Blots are representative of three biological replicates.

 © 2022 The Authors

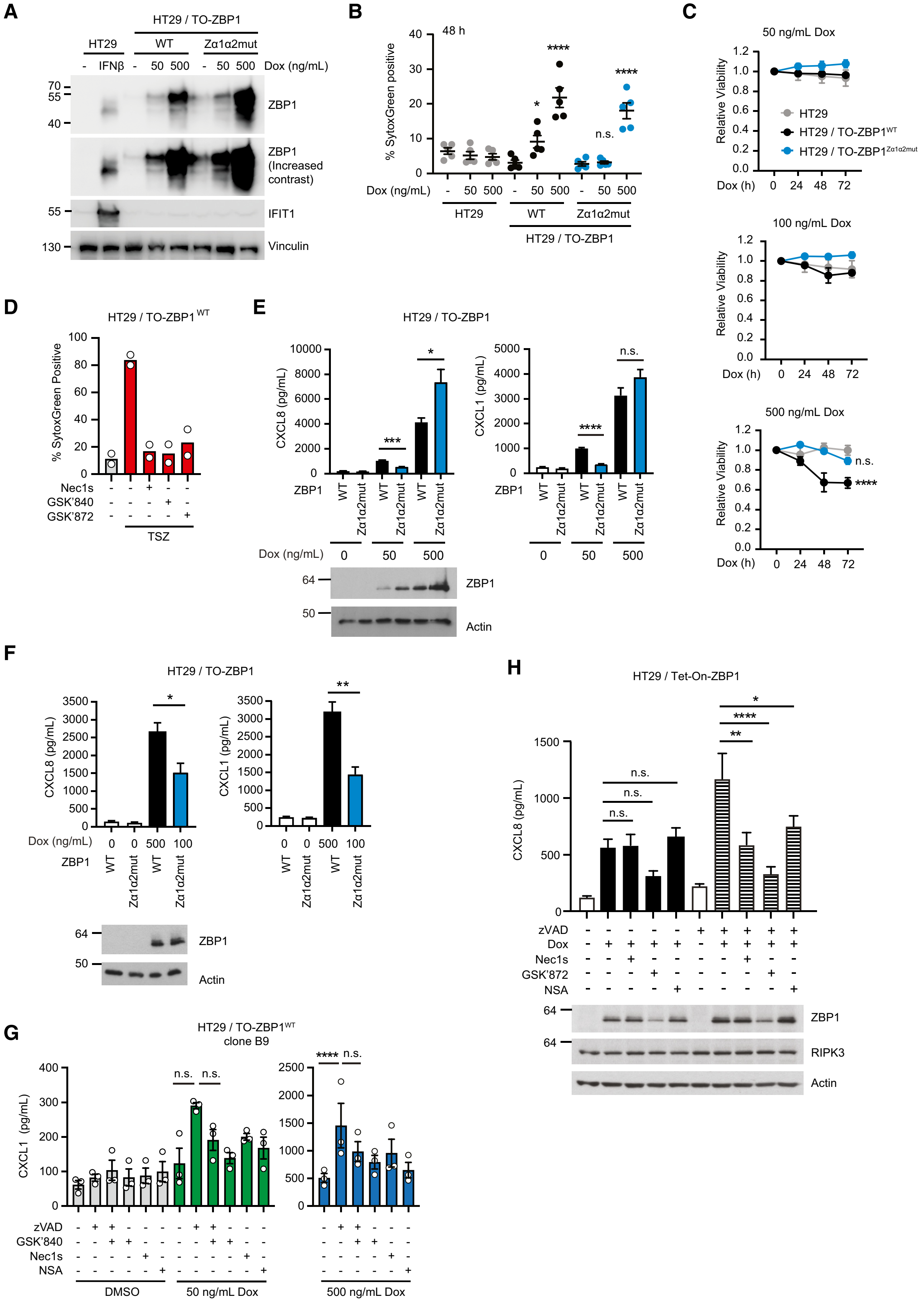
A
HT29 / TO-ZBP1
HT29
WT
Zα1α2mut
- IFNβ - 50 500 - 50 500 Dox (ng/mL)
70
55
40
ZBP1
ZBP1 (Increased contrast)
55
IFIT1
130
Vinculin
B
48 h
% SytoxGreen positive
Dox (ng/mL)
HT29
WT
Zα1α2mut
HT29 / TO-ZBP1
n.s.
C
50 ng/mL Dox
100 ng/mL Dox
500 ng/mL Dox
Relative Viability
HT29
HT29 / TO-ZBP1WT
HT29 / TO-ZBP1Zα1α2mut
Dox (h)
D
HT29 / TO-ZBP1 WT
% SytoxGreen Positive
Nec1s
GSK'840
GSK'872
TSZ
E
HT29 / TO-ZBP1
CXCL8 (pg/mL)
CXCL1 (pg/mL)
ZBP1
WT
Zα1α2mut
Dox (ng/mL)
ZBP1
Actin
F
HT29 / TO-ZBP1
CXCL8 (pg/mL)
CXCL1 (pg/mL)
Dox (ng/mL)
ZBP1
Actin
G
HT29 / TO-ZBP1WT
clone B9
CXCL1 (pg/mL)
zVAD
GSK'840
Nec1s
NSA
DMSO
50 ng/mL Dox
500 ng/mL Dox
H
HT29 / Tet-On-ZBP1
CXCL8 (pg/mL)
zVAD
Dox
Nec1s
GSK'872
NSA
ZBP1
RIPK3
Actin

Figure EV1.

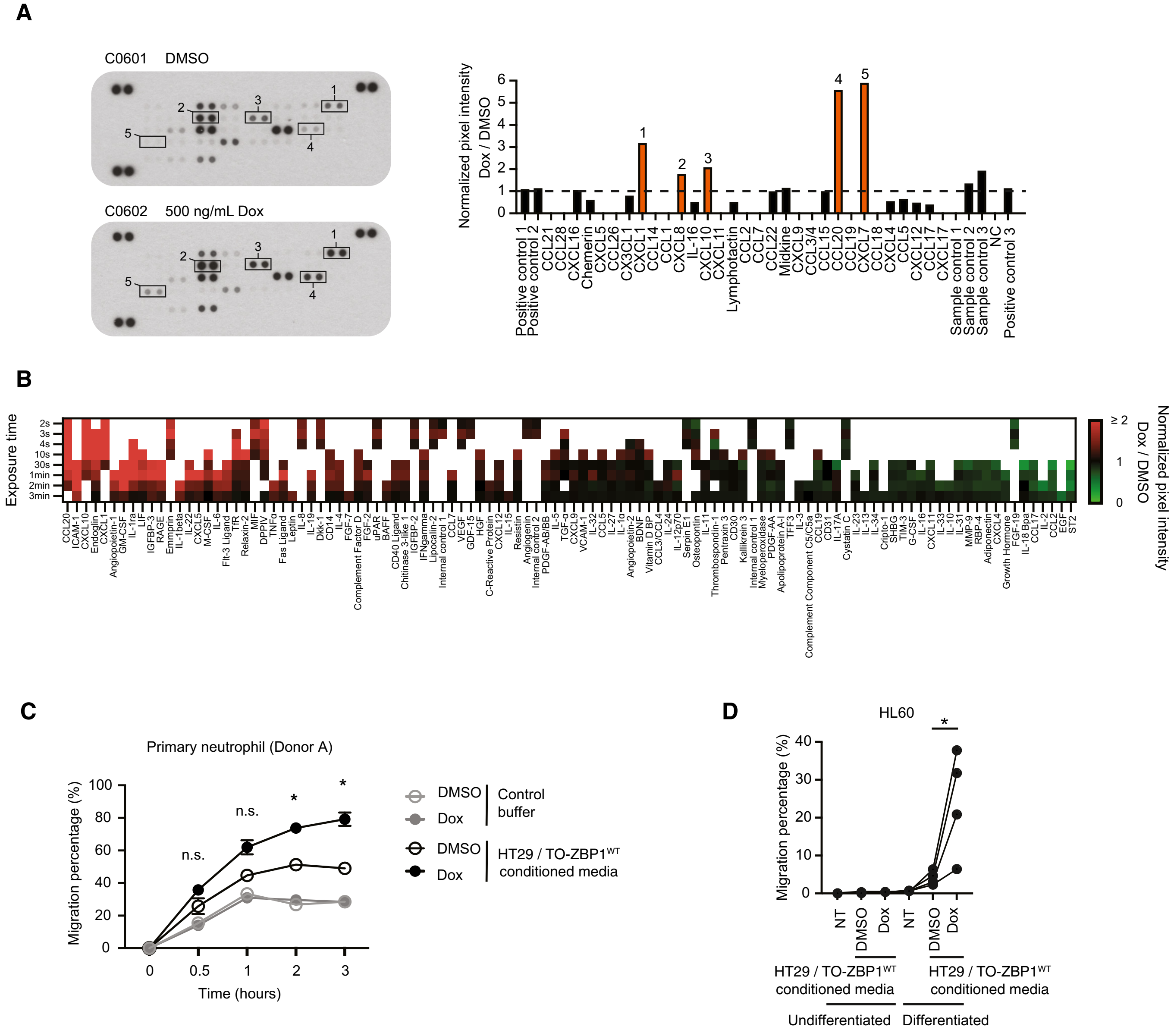

Figure EV2. ZBP1 expression-induced cytokine secretome promotes the chemotaxis of neutrophils.

A, B Chemokine (A) and cytokine (B) arrays of the conditioned media from HT29/TO-ZBP1^{WT} cells treated with DMSO or 500 ng/ml Dox for 24 h. Normalized pixel intensity values are presented relative to values from DMSO-treated samples ($n = 1$ biological replicate).

C Transwell migration of primary neutrophils from Donor A towards conditioned media from HT29/TO-ZBP1^{WT} cells treated with DMSO or 500 ng/ml Dox for 24 h, or control buffer containing equal volume and amount of DMSO or Dox as the conditioned media. Data are presented as mean with S.E.M. ($n = 3$ biological replicates). Two-way ANOVA and Dunnet's multiple comparison tests were used to test for statistical differences between the migration of neutrophils toward conditioned media from DMSO-treated cells and that from Dox-treated cells at each time point. n.s. = not significant ($P > 0.05$); *$P = 0.0113$ for the 2 h time point, $P = 0.0133$ for the 3 h time point.

D Transwell migration of differentiated or undifferentiated HL60 cells toward control buffer (NT) or conditioned media from HT29/TO-ZBP1^{WT} cells treated with DMSO or 500 ng/ml Dox for 24 h. Data from each biological replicate of differentiated HL60 cells and conditioned media are connected with solid lines ($n = 4$ biological replicates). A paired t-test were used to determine the statistical difference between indicated conditions. *$P = 0.0447$.

 © 2022 The Authors

Figure EV3. RIPK1 mediates ZBP1-induced inflammatory signaling and cell death.

A CXCL8 and CXCL1 concentration in the culture media of HT29/TO-ZBP1^{WT} Clone B9 cells following 24 h treatment with 0 or 500 ng/ml Dox in combination with 10 μM GSK'840 or 10 μM GSK'872 as indicated. Data are presented as mean with S.E.M. ($n = 3$ biological replicates). Two-way ANOVA tests and Tukey's multiple comparisons test were used to test for statistical differences between indicated conditions. n.s., not significant ($P \geq 0.05$); *$P = 0.0174$; ***$P = 0.0006$; ****$P < 0.0001$.

B Cytokine concentration in the culture media of HT29/TO-ZBP1^{WT} or two independent clones of HT29/RIPK1-KO/TO-ZBP1^{WT} cells treated with Dox as indicated. N.D., not detected. Data are presented as mean with S.E.M. ($n = 3$ biological replicates). One-way ANOVA and Sidak's multiple comparisons test were used to test for statistical differences between indicated conditions. n.s. = not significant ($P > 0.05$); **$P = 0.0079$ for CXCL1, $P = 0.0012$ for CXCL8; ***$P = 0.0008$; ****$P < 0.0001$. Cell lysates from the same wells were analyzed by Western blotting for ZBP1 and RIPK1 levels. Blots are representative of three biological replicates.

C Relative viability of HT29/TO-ZBP1^{WT} cells or two independent clones of HT29/RIPK1-KO/TO-ZBP1^{WT} cells after treatment with or without 500 ng/ml Dox for up to 3 days as indicated. Viability is normalized to no Dox-treatment within the same cell line. Data are presented as mean with S.E.M. ($n = 3$ biological replicates). Two-way ANOVA and Sidak's multiple comparison tests were used to test for statistical differences between WT and RIPK1-KO cells after Dox treatment. ****$P < 0.0001$.

D Two biological replicates of Ub-conjugate enrichment analysis shown in Fig 3C.

© 2022 The Authors

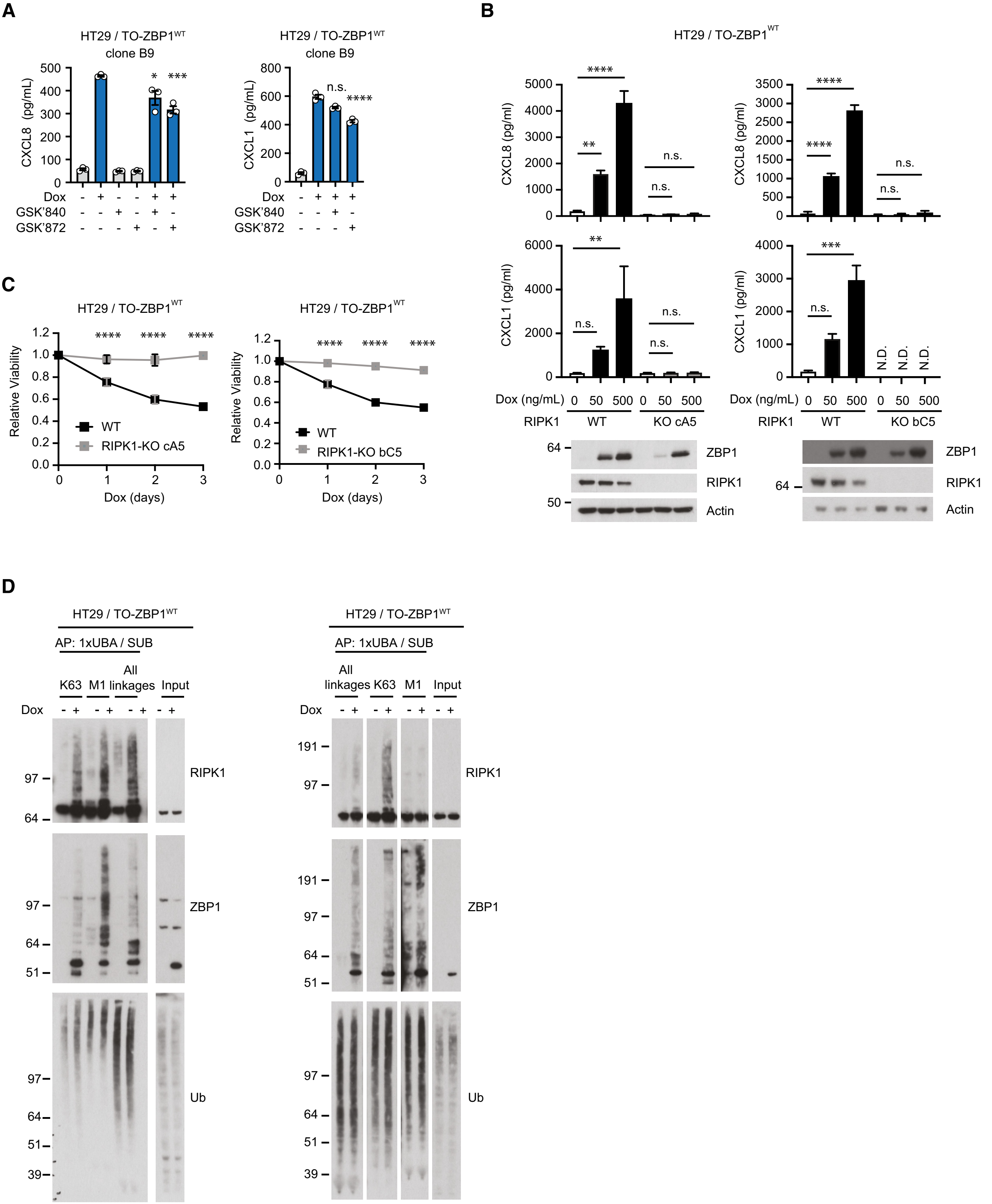

Figure EV3.

© 2022 The Authors

Figure EV4. RIPK3 oligomerization induces inflammatory signaling.

A Relative viability of HT29 or HT29/RIPK3-2xFV cells pretreated with combinations of 100 nM CpA, 20 μM zVAD, and 10 μM Nec1s or DMSO (−) as indicated for 1 h, and then treated with or without 2 ng/ml TNF for 24 h. Viability is normalized to the DMSO-only condition for each cell line. Data are presented as mean with S.E.M. (n = 3 biological replicates). Two-way ANOVA and Sidak's multiple comparison test were used to test for statistical differences between indicated conditions and DMSO-treated condition of each cell line. **P = 0.0044; ****P < 0.0001.

B Western blot analysis of necroptosis markers in HT29 cells pretreated with 100 nM CpA and 20 μM zVAD for 1 h before stimulating with 20 ng/ml TNF as indicated. Blots are representative of four biological replicates.

C Western blot analyses of inflammatory marker proteins in HT29/RIPK3-2xFV cells treated with 100 nM dimerizer. Blots are representative of two biological replicates.

D, E Relative *CXCL8* expression (D) and CXCL8 concentration in culture media (E) of HT29/RIPK3-2xFV cells treated with 0 or 100 nM dimerizer as indicated. Data are presented as mean with S.E.M. (n = 3 biological replicates). A Welch's t-test was used to test for statistical differences between indicated conditions. **P = 0.0016 (D); **P = 0.0093 (E).

F Relative viability of HT29/RIPK3-2xFV cells treated with 0 or 100 nM dimerizer for 24 h in the presence of 0.5 μg/ml mouse IgG. Data are presented as mean with S.E.M. (n = 3 biological replicates). A Welch's t-test was used to test for statistical differences between indicated conditions. n.s. = not significant (P = 0.3268).

G, H Western blot analysis of (G) HEK293FT/RIPK3-2xFV cells (FL or ΔC) and (H) U2OS/NOD2 cells stably expressing RIPK3 variants treated with 100 nM dimerizer as indicated. Blots are representative of two biological replicates.

I Time course of relative *CXCL8* and *TNF* expression in U2OS/NOD2/RIPK3-2xFV cells (FL or ΔC) treated with 100 nM dimerizer as indicated. Data are plotted as mean with S.E.M. (n = 3 biological replicates). Two-way ANOVA and Tukey's multiple comparisons test were used to test for statistical differences between 0 h and indicated time points in FL cells. **P = 0.0021 for 2 h, P = 0.0010 for 3 h; n.s. = not significant (P = 0.6323); ****P < 0.0001.

J, K Relative *CXCL8* and *TNF* expression (J) and CXCL8 concentration in the culture media (K) of HT29/RIPK3-2xFV cells pretreated or not with 2 μM CpA for 1 h before treated or not with 100 nM dimerizer for 3 h. Data are plotted as mean with S.E.M. (n = 3 biological replicates). Unpaired t-tests were used to test for statistical differences between indicated conditions. ****P < 0.0001; *P = 0.0145 (J). A Welch's t-test was used to test for statistical differences between indicated conditions. **P = 0.0097 (K).

L Relative *CXCL8* expression in WT or XIAP-knockout HCT116/RIPK3-2xFV cells treated with or without 100 nM dimerizer for 3 h. Data are plotted as mean with S.E.M. (n = 3 biological replicates). A Welch's t-test were used to test for statistical differences between indicated conditions. n.s. = not significant (P = 0.2057). Cell lysates were analyzed by Western blotting. Blots are representative of two biological replicates.

© 2022 The Authors

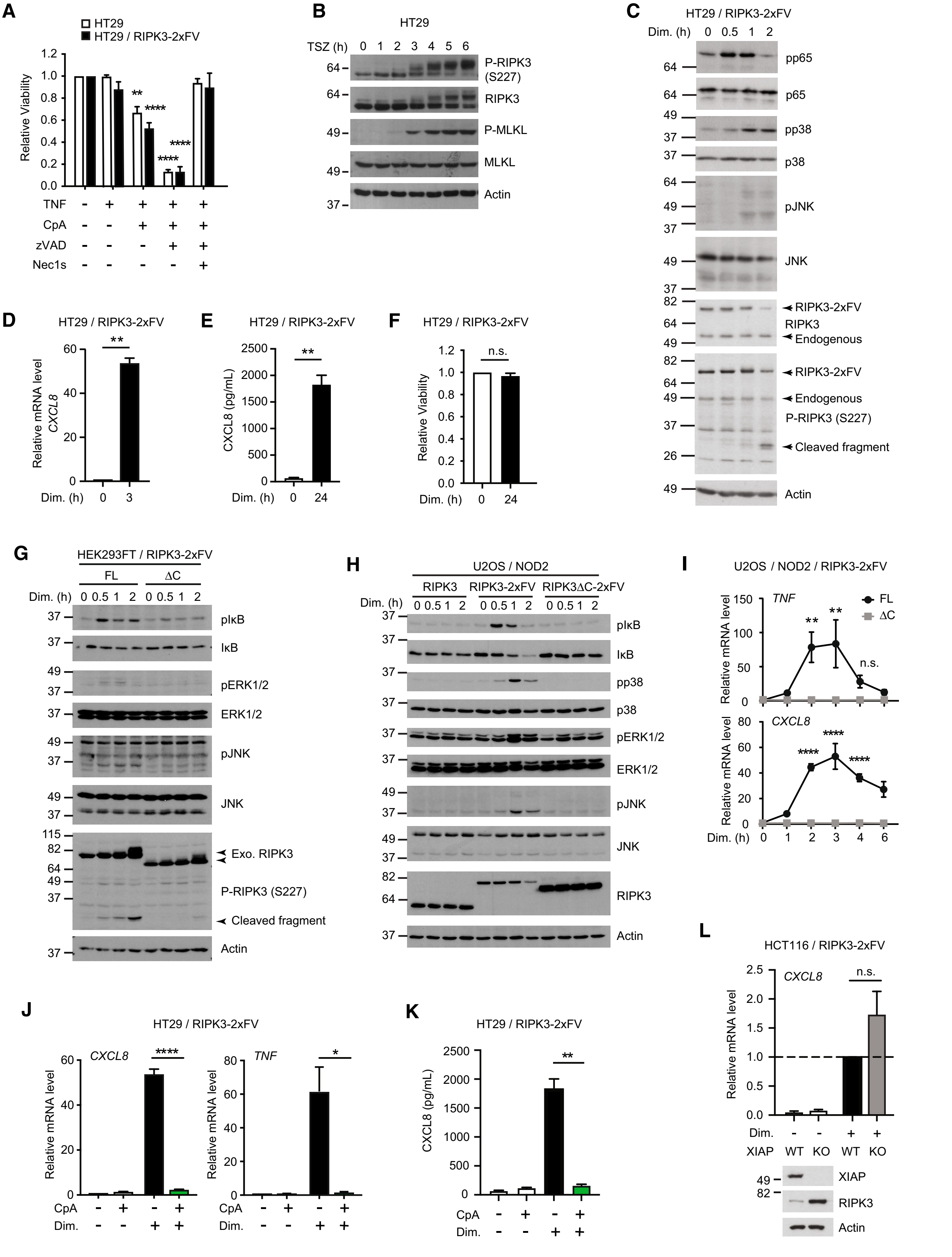

Figure EV4.

 © 2022 The Authors

Figure EV5. ZBP1 mediates SARS-CoV-2-induced inflammation.

A Relative expression of *ZBP1* in post-mortem lung samples of two COVID-19 patients compared with healthy lung biopsies (Blanco-Melo *et al*, 2020a, Data ref: Blanco-Melo *et al*, 2020b).

B Patient-averaged single cell Transcript Per Million (TPM) values of *ZBP1* in lung and peripheral blood of COVID-19 patients in progressive or convalescent stage, compared to healthy controls (Ren *et al*, 2021a, Data ref: Ren *et al*, 2021b). Data are plotted as individual values per patient with mean and S.E.M. ($n = 25$ for healthy, $n = 77$ for progression, $n = 102$ for convalescence patients). Kruskai–Wallis test and Dunn's multiple comparisons test were used to test for statistical differences between indicated conditions. ****$P < 0.0001$; *$P = 0.0375$; **$P = 0.0036$.

C Pearson's correlation of individual single cell TPM values of indicated genes with virus load (Ren *et al*, 2021a, Data ref: Ren *et al*, 2021b). Data are presented as individual values from each cell, with linear regression line and its 95% confidence bands. *ZBP1*, $n = 720$, *IL*-6, $n = 94$, *TNF*, $n = 211$, *CXCL10*, $n = 1{,}053$, *CXCL8*, $n = 1{,}463$, *ACTB*, $n = 2{,}683$ (cells). The positive correlation is significant ($P < 0.0001$) between TPM (virus) and TPM (*ZBP1*), TPM (*IL*-6), TPM (*TNF*), TPM (*CXCL10*), or TPM (*CXCL8*). Correlation between TPM (virus) and TPM (*ACTB*) is not significant ($P = 0.7862$).

D Fold of mRNA levels of SARS-CoV-2-encoded *N1* and *N2* (C) over β-Actin (ACTB, control), at indicated time after infection in Calu-3 cells, normalized over mock 16 h. Data is presented as mean with S.E.M ($n = 3$ biological replicates). Two-way ANOVA and Tukey's multiple comparison tests were used to test for the statistical differences between each time point with 16 h within the same MOI. n.s., not significant ($P > 0.4$); *$P = 0.0376$; ****$P < 0.0001$.

E Western blot analysis of SARS-CoV-2-infected Calu-3 cells at MOI = 2 for the indicated time. Analysis was performed on one biological replicate.

F, G Expression of *IFNB1* and *IFIT1* (F) or SARS-CoV-2-encoded *N1* and *N2* (G) relative to β-Actin in Calu-3 cells with stable knockdown of *ZBP1* (shZBP1-50, shZBP1-52 and shZBP1-53) and control cells (shMM) infected with mock or SARS-CoV-2 virus at MOI = 2 for 72 h. Data are presented as mean with S.E.M ($n = 3$ biological replicates). One-way ANOVA and Sidak's multiple comparisons test were used to test for statistical differences between indicated conditions. n.s. not significant ($P = 0.3831$); ***$P = 0.0006$ for *IFNB1*, $P = 0.0003$ for *IFIT1*; *$P = 0.0143$; ****$P < 0.0001$ (F) and to test for statistical differences between shMM MOI = 2 and indicated conditions. *$P = 0.0294$ for shZBP1-50, $P = 0.0259$ for shZBP1-53; ***$P = 0.0008$; n.s., not significant ($P > 0.05$) (G).

© 2022 The Authors

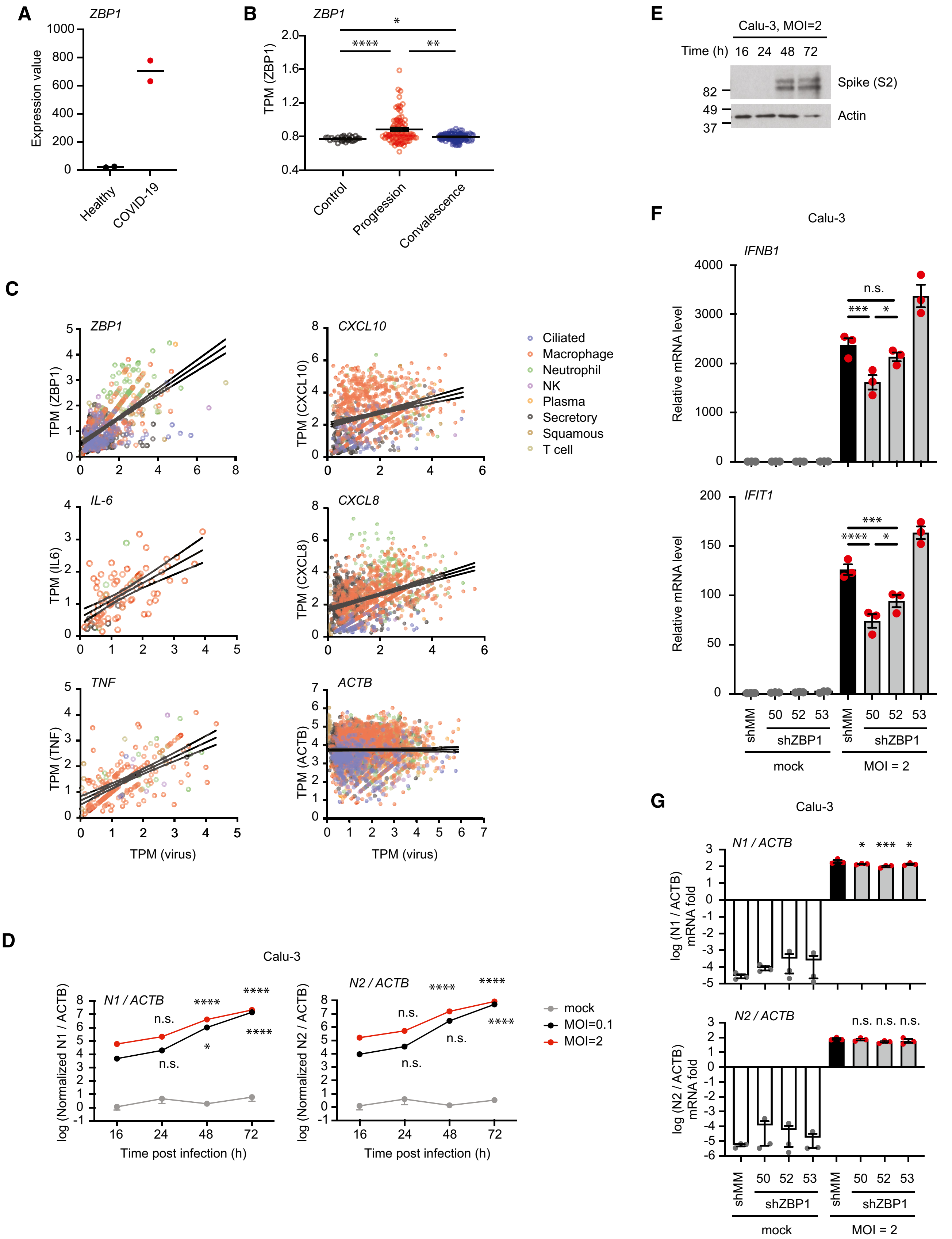

Figure EV5.

 © 2022 The Authors
