# Supplementary material for: Human ZBP1 induces cell death‐independent inflammatory signaling via RIPK3 and RIPK1
Source: EMBO Rep. 2022 Oct 21;23(12):e55839. doi: 10.15252/embr.202255839 (PMC9724671; doi:10.15252/embr.202255839)
Supplement: Supplementary file 2 — Table EV1 [file EMBR-23-e55839-s010.docx]

Table EV1. Mutations in HT29 / RIPK1 KO cells

| HT29 RIPK1 KO CRISPR/Cas9 mutations and effect on protein | | | |
| --- | --- | --- | --- |
| **Gene** | **Clone** | **Protein** | **Gene** |
| RIPK1 | aA3 | p.N199X | c.597-598insT |
| RIPK1 | aA3 | p.L198X | c.595-596del |
| RIPK1 | cA5 | p.M101IfsX23 | c.302dupT |
| RIPK1 | cA5 | p.M101IfsX25 | c.302-303ins121bp |
| RIPK1 | bC5 | p.Y191X | c.573-574del |
| RIPK1 | bC5 | p.D180EfsX5 | c.539-687del |
| RIPK1 | bC5 | p.A183QfsX11 | c.546-615del |
