# Supplementary figures and images for: Human ZBP1 induces cell death‐independent inflammatory signaling via RIPK3 and RIPK1
Source: EMBO Rep. 2022 Oct 21;23(12):e55839. doi: 10.15252/embr.202255839 (PMC9724671; doi:10.15252/embr.202255839)

Figure EV1

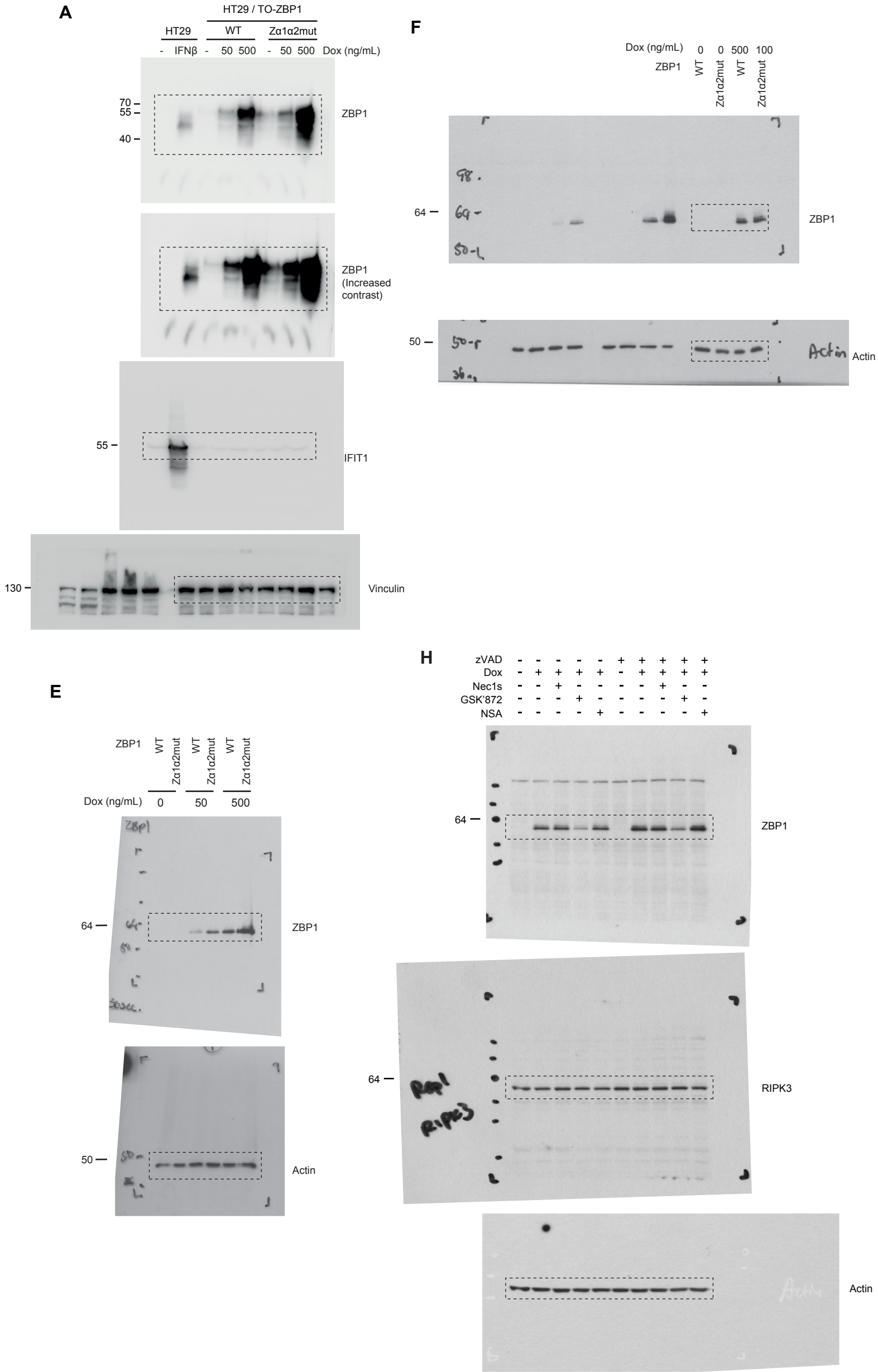

Supplement: Supplementary file 3 — Source Data for Expanded View [file EMBR-23-e55839-s011.zip › EMBOR-2022-55839V3-Figure_EV1_Source_Data-sd.pdf]

Figure EV3

B

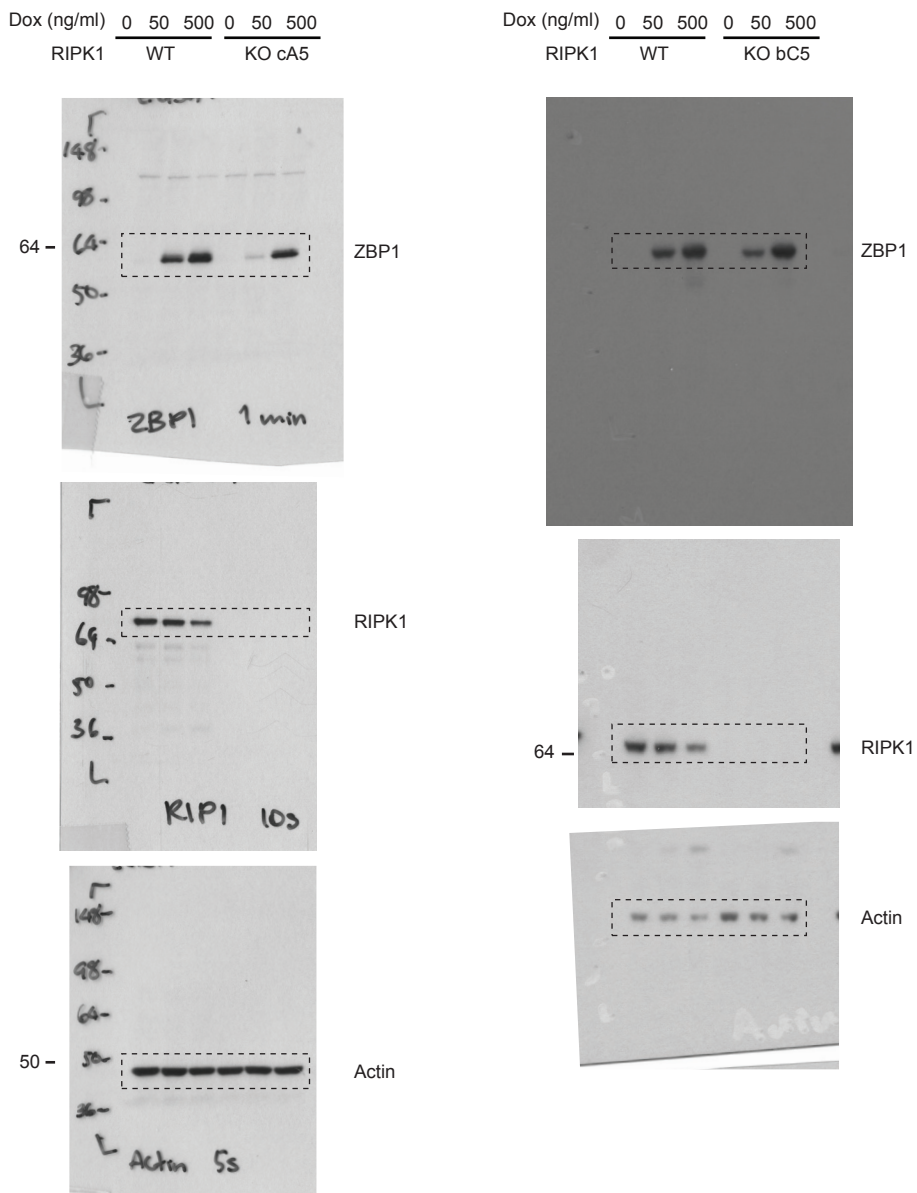

D

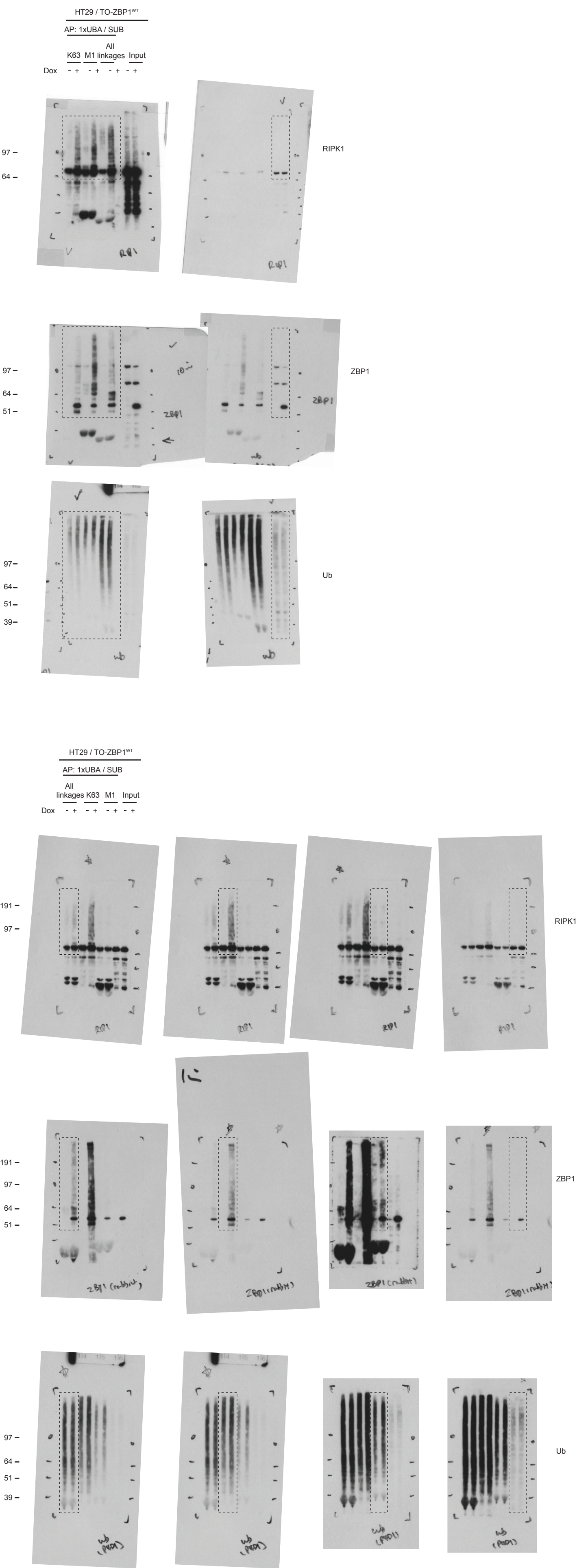

Supplement: Supplementary file 3 — Source Data for Expanded View [file EMBR-23-e55839-s011.zip › EMBOR-2022-55839V3-Figure_EV3_Source_Data-sd.pdf]

Figure 1

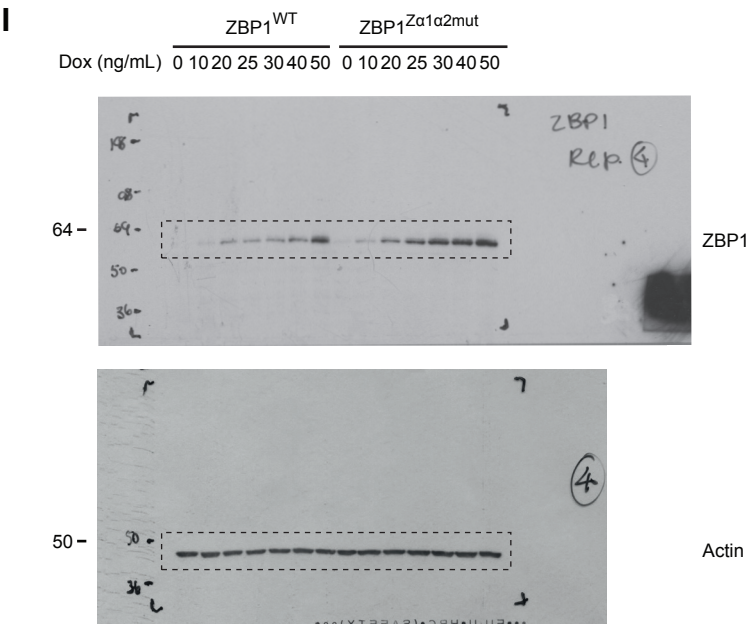

Supplement: Supplementary file 4 — Source Data for Figure 1 [file EMBR-23-e55839-s007.pdf]

Figure 2

D

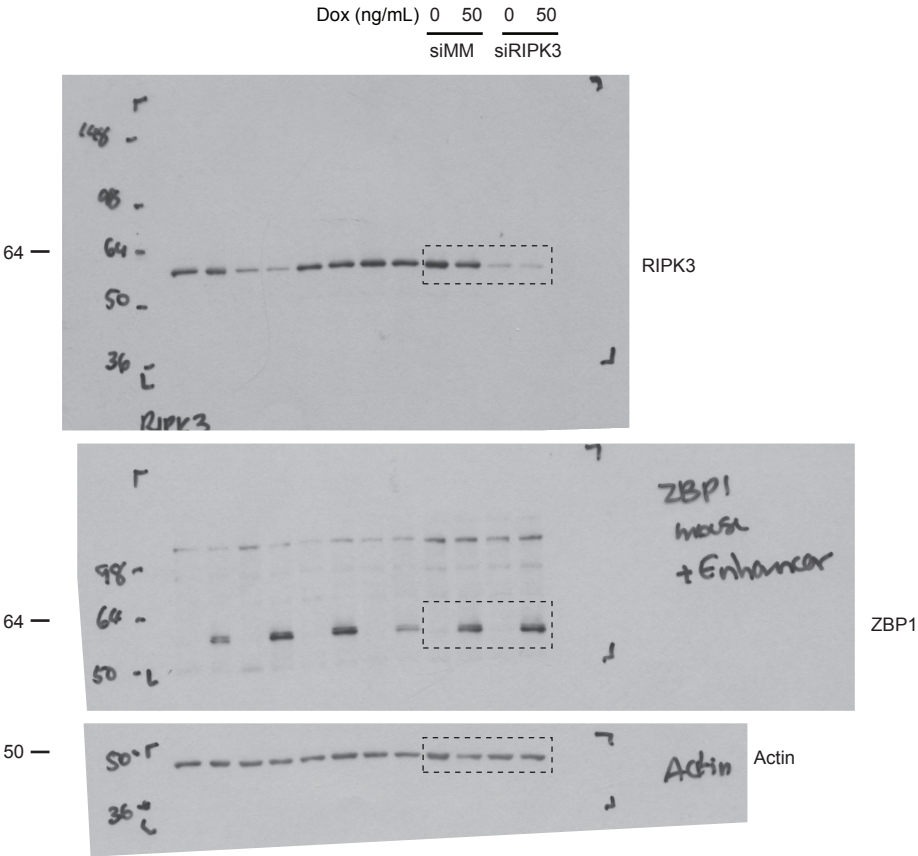

E

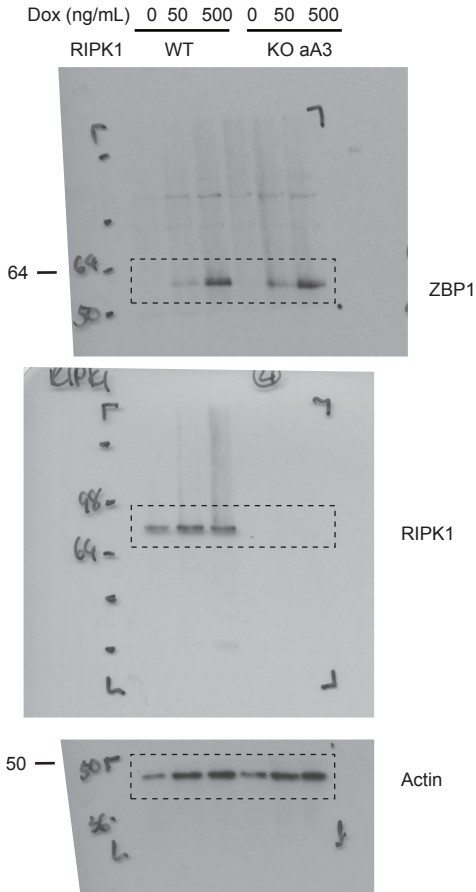

G

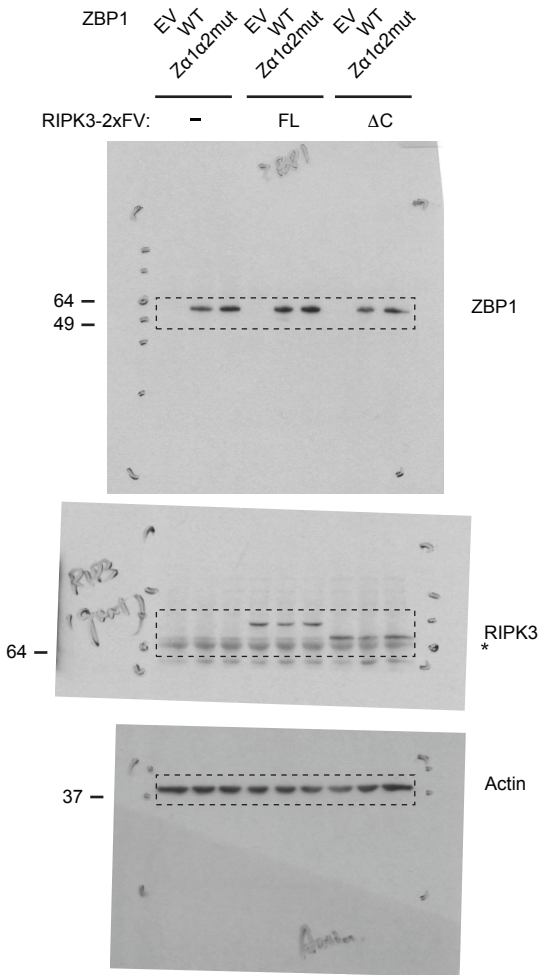

Supplement: Supplementary file 5 — Source Data for Figure 2 [file EMBR-23-e55839-s005.pdf]

Figure 3

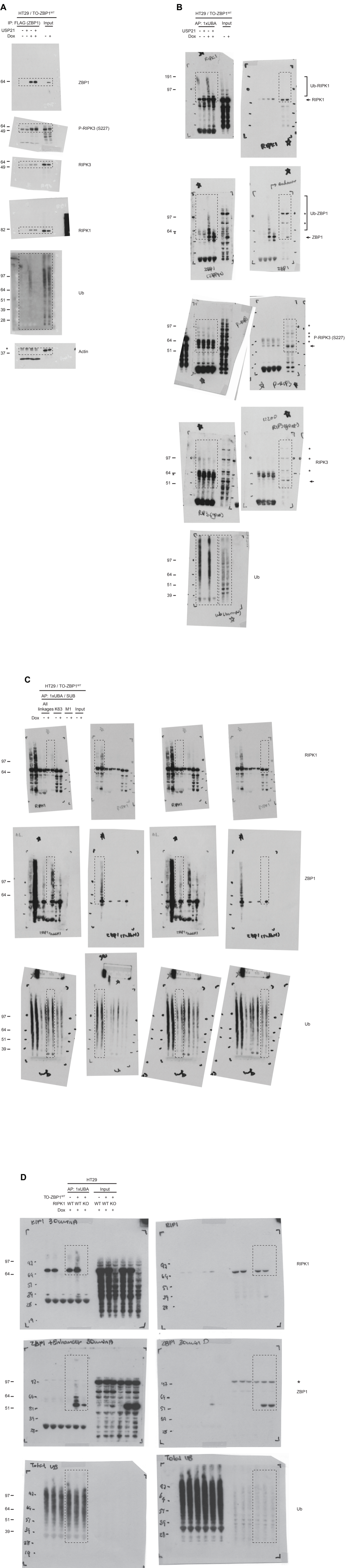

Supplement: Supplementary file 6 — Source Data for Figure 3 [file EMBR-23-e55839-s003.pdf]

Figure 4

D

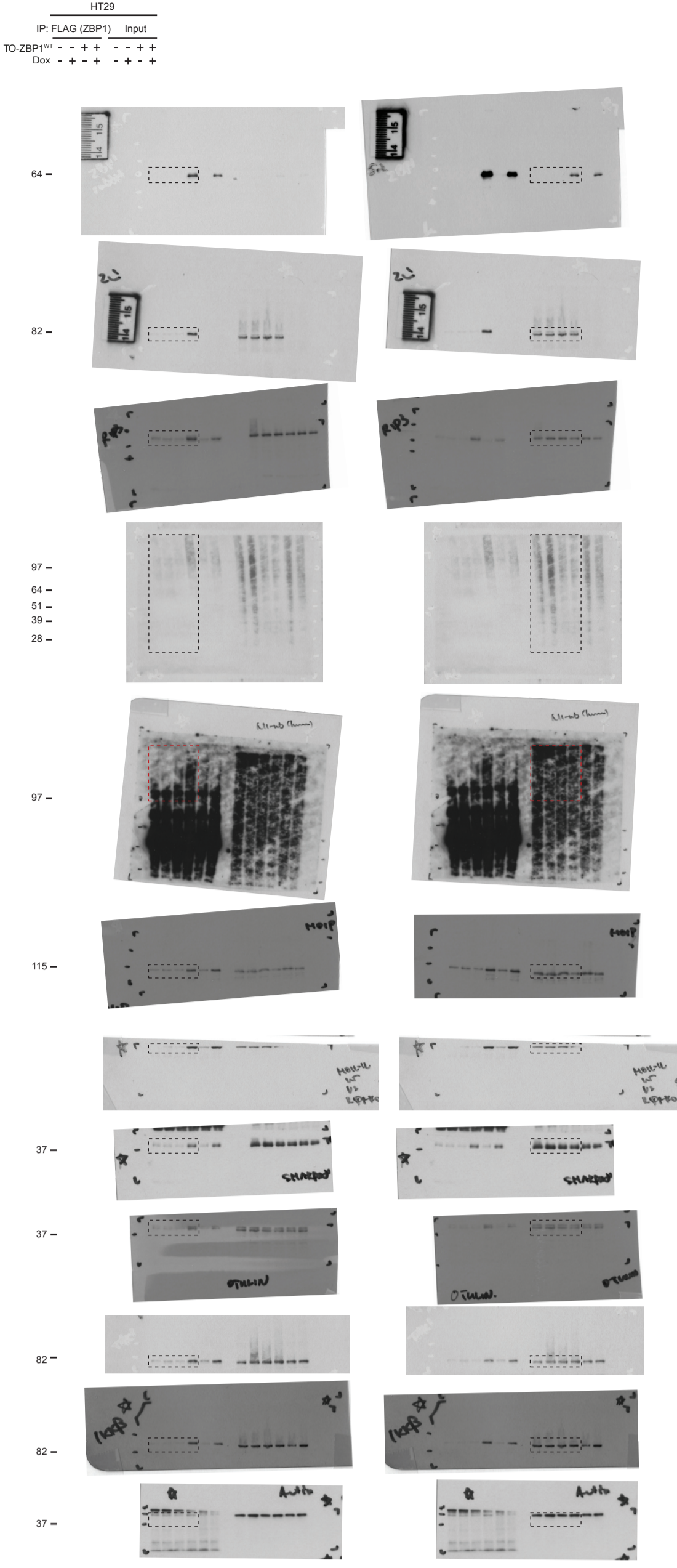

E

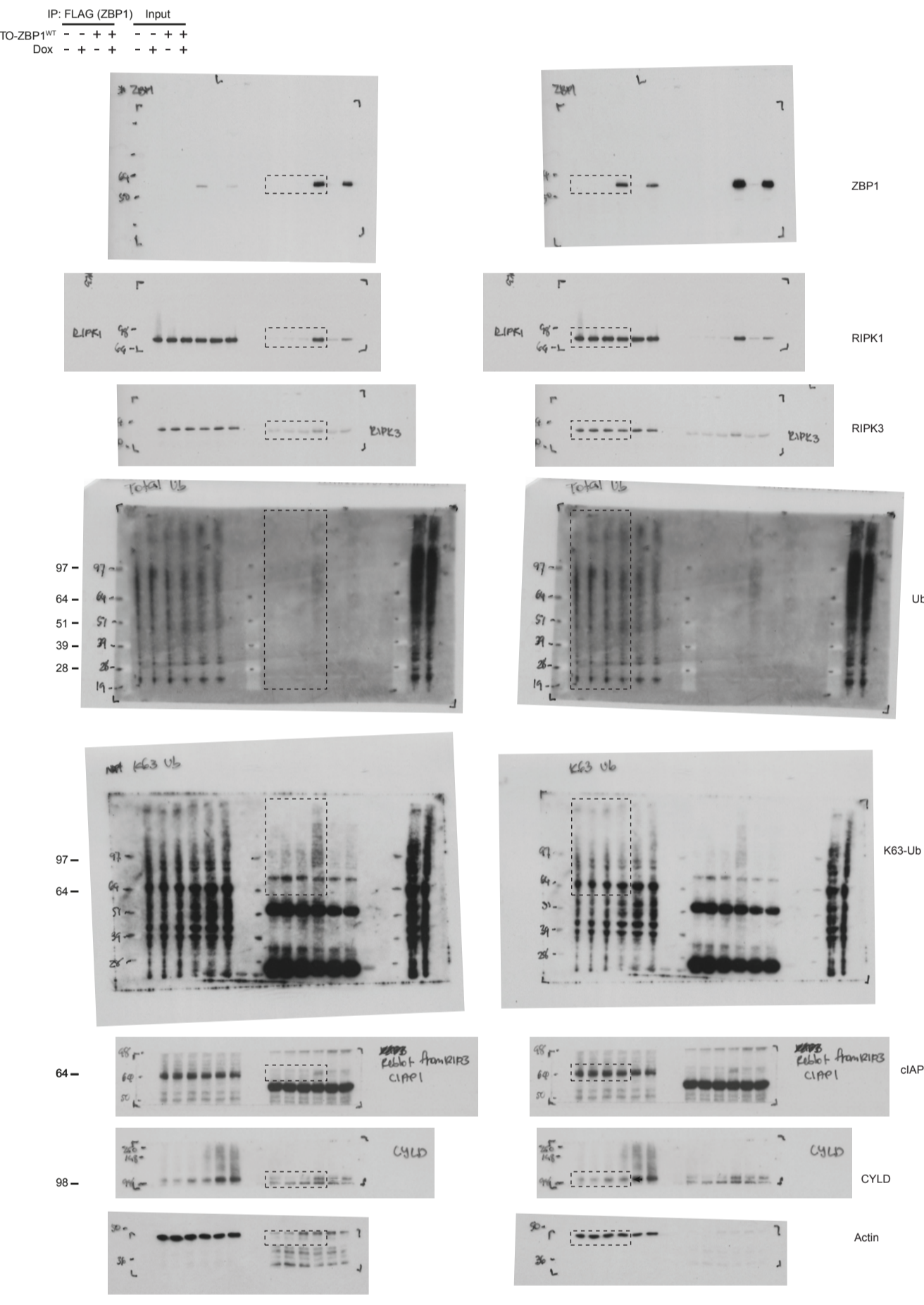

F

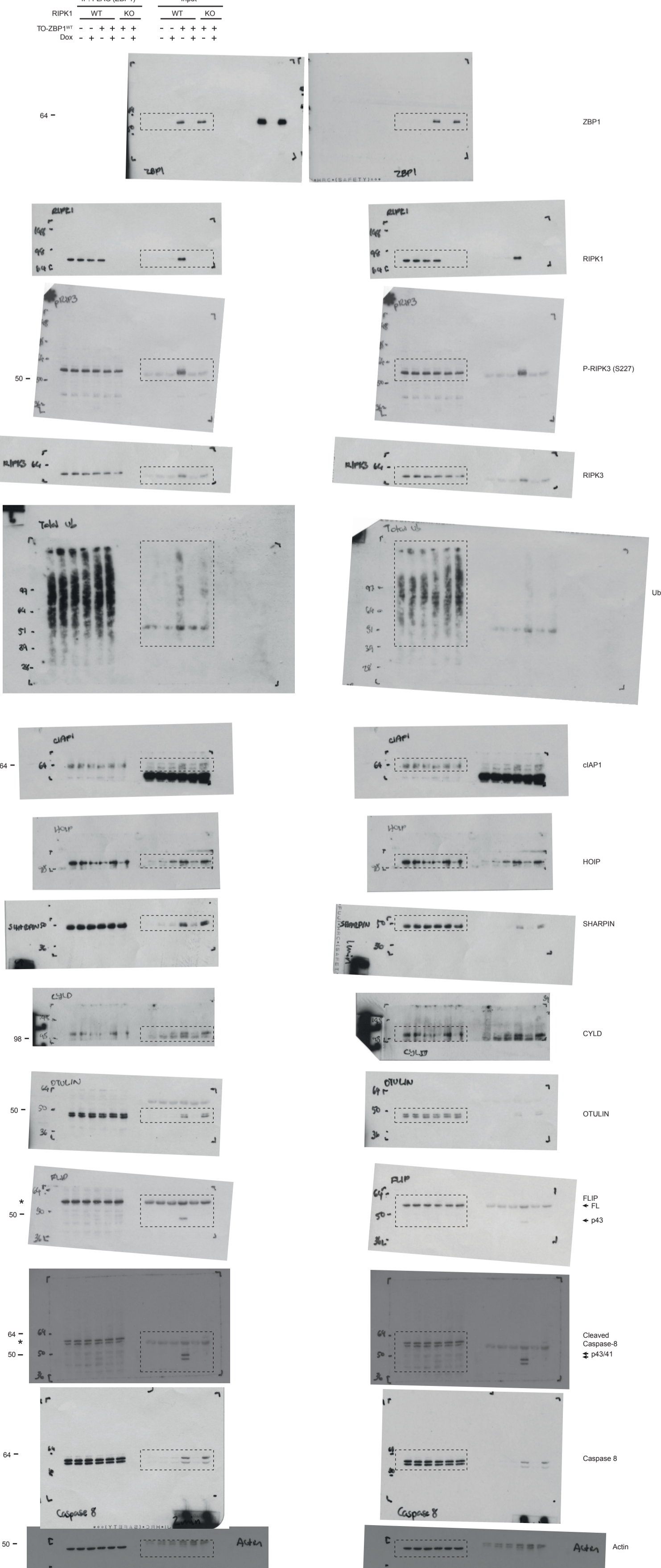

Supplement: Supplementary file 7 — Source Data for Figure 4 [file EMBR-23-e55839-s004.pdf]

Figure 5

B

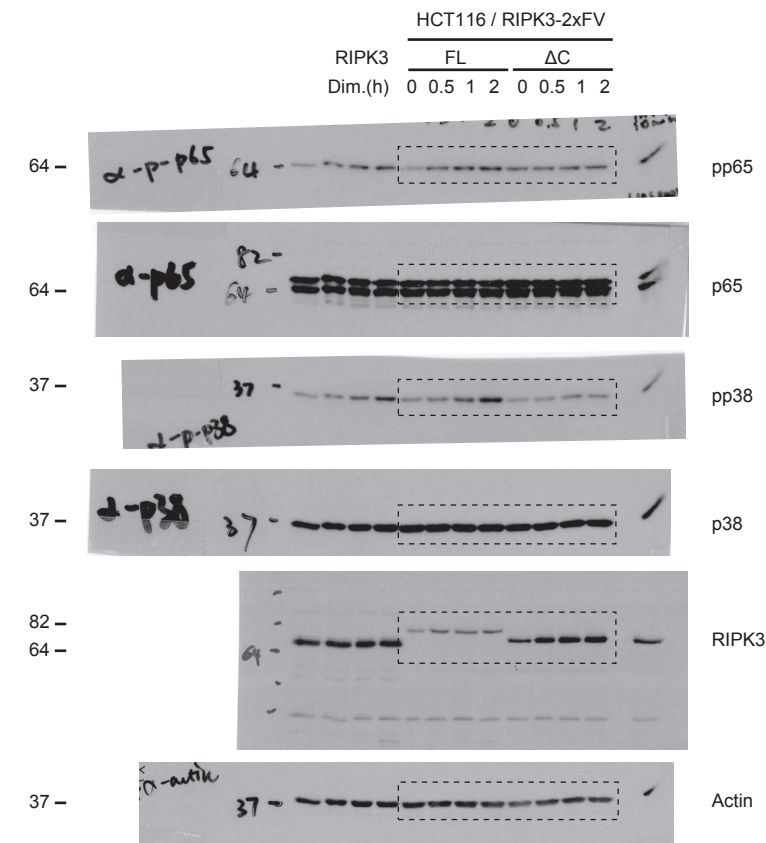

Supplement: Supplementary file 8 — Source Data for Figure 5 [file EMBR-23-e55839-s002.pdf]

Figure 6

A

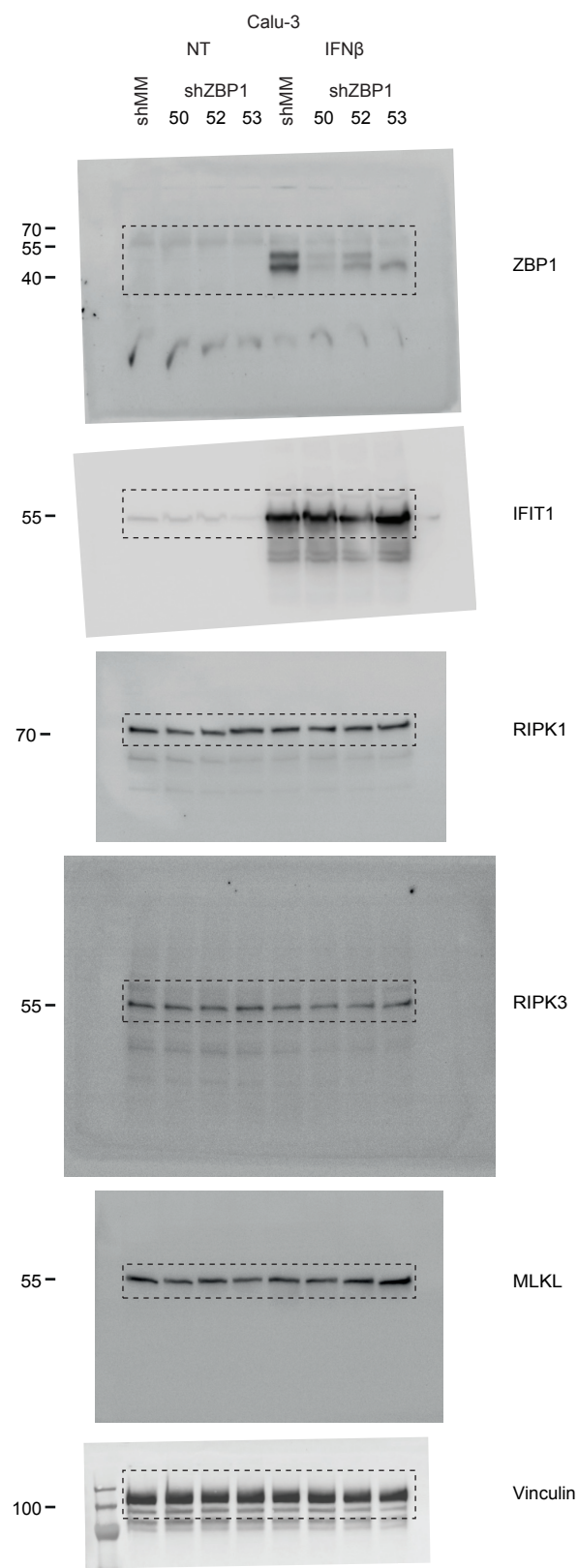

Supplement: Supplementary file 9 — Source Data for Figure 6 [file EMBR-23-e55839-s008.pdf]
