# Supplementary material for: Human ZBP1 induces cell death‐independent inflammatory signaling via RIPK3 and RIPK1
Source: EMBO Rep. 2022 Oct 21;23(12):e55839. doi: 10.15252/embr.202255839 (PMC9724671; doi:10.15252/embr.202255839)
Supplement: Supplementary file 10 — PDF+ [file EMBR-23-e55839-s006.pdf]

Article

SOURCE DATA | TRANSPARENT PROCESS | OPEN ACCESS

EMBO reports

# Human ZBP1 induces cell death-independent inflammatory signaling via RIPK3 and RIPK1

Ruoshi Peng[1], Chris Kedong Wang[2], Xuan Wang-Kan[1], Manja Idorn[3], Majken Kjær[2], Felix Y Zhou[1], Berthe Katrine Fiil[2], Frederik Timmermann[2], Susana L Orozco[4], Julia McCarthy[1], Carol S Leung[1], Xin Lu[1], Katrin Bagola[1,5], Jan Rehwinkel[6], Andrew Oberst[4], Jonathan Maelfait[7,8], Søren R Paludan[3] & Mads Gyrd-Hansen[1,2,*]

## Abstract

**ZBP1 is an interferon-induced cytosolic nucleic acid sensor that facilitates antiviral responses via RIPK3. Although ZBP1-mediated programmed cell death is widely described, whether and how it promotes inflammatory signaling is unclear. Here, we report a ZBP1-induced inflammatory signaling pathway mediated by K63- and M1-linked ubiquitin chains, which depends on RIPK1 and RIPK3 as scaffolds independently of cell death. In human HT29 cells, ZBP1 associated with RIPK1 and RIPK3 as well as ubiquitin ligases cIAP1 and LUBAC. ZBP1-induced K63- and M1-linked ubiquitination of RIPK1 and ZBP1 to promote TAK1- and IKK-mediated inflammatory signaling and cytokine production. Inhibition of caspase activity suppressed ZBP1-induced cell death but enhanced cytokine production in a RIPK1- and RIPK3 kinase activity-dependent manner. Lastly, we provide evidence that ZBP1 signaling contributes to SARS-CoV-2-induced cytokine production. Taken together, we describe a ZBP1-RIPK3-RIPK1-mediated inflammatory signaling pathway relayed by the scaffolding role of RIPKs and regulated by caspases, which may induce inflammation when ZBP1 is activated below the threshold needed to trigger a cell death response.**

**Keywords** inflammatory signaling; RIPK1; RIPK3; SARS-CoV-2; ZBP1
**Subject Categories** Immunology; Post-translational Modifications & Proteolysis; Signal Transduction
**DOI** 10.15252/embr.202255839 | Received 22 July 2022 | Revised 6 October 2022 | Accepted 7 October 2022 | Published online 21 October 2022
**EMBO Reports (2022) 23: e55839**

## Introduction

Inflammation and cell death underlie antiviral innate immune responses and contribute to pathological inflammatory conditions when deregulated. Receptor interacting protein (RIP) kinases (RIPKs), engaged downstream of immune receptors, are central regulators of cell death and inflammatory signaling pathways and contribute to host immune defenses against viruses and bacteria (He & Wang, 2018; Newton, 2020; Topal & Gyrd-Hansen, 2021).

Z-DNA-binding protein 1 (ZBP1) is a cytosolic nucleic acid sensor and an interferon-induced pattern recognition receptor (PRR) important for antiviral immune responses (Takaoka *et al*, 2007; Upton *et al*, 2012; Pham *et al*, 2013; Omoto *et al*, 2015; Kuriakose *et al*, 2016; Thapa *et al*, 2016; Kesavardhana *et al*, 2017; Maelfait *et al*, 2017; Daniels *et al*, 2018; Kuriakose & Kanneganti, 2018). After activation, ZBP1 recruits RIPK3 and RIPK1 to execute programmed cell death; necroptosis as well as apoptosis and pyroptosis depending on the cell type and caspase activity (Upton *et al*, 2012; Thapa *et al*, 2016).

RIPK3 signals for necroptosis by phosphorylating MLKL, which in turn oligomerizes and forms pores in the cell membrane (Cho *et al*, 2009; He *et al*, 2009; Sun *et al*, 2012). In addition to its kinase domain, RIPK3 contains a RIP Homotypic Interaction Motif (RHIM) that mediates its recruitment to other RHIM-containing proteins, namely RIPK1, the Toll-like receptor (TLR) adaptor TIR-domain-containing adapter-inducing interferon-β (TRIF) and ZBP1 (Kaiser *et al*, 2008, 2013; He *et al*, 2009; Rebsamen *et al*, 2009). The activation of RIPK3 is proposed to occur within a RHIM-mediated oligomer enucleated by the RHIM of RIPK1 and stabilized by phosphorylation of RIPK1 and RIPK3 molecules (Li *et al*, 2012; Wu *et al*, 2014). In addition to its necroptosis-promoting activity, RIPK3 has been suggested to promote inflammatory signaling during TNF- and TLR-induced necroptosis and downstream of ZBP1 (Kaiser

1 Nuffield Department of Medicine, Ludwig Institute for Cancer Research, University of Oxford, Oxford, UK
2 Department of Immunology and Microbiology, LEO Foundation Skin Immunology Research Center, University of Copenhagen, Copenhagen, Denmark
3 Department of Biomedicine, Aarhus University, Aarhus C, Denmark
4 Department of Immunology, University of Washington, Seattle, WA, USA
5 Division of Immunology, Federal Institute for Vaccines and Biomedicines, Paul-Ehrlich-Institut, Langen, Germany
6 MRC Human Immunology Unit, Radcliffe Department of Medicine, MRC Weatherall Institute of Molecular Medicine, University of Oxford, Oxford, UK
7 VIB-UGent Center for Inflammation Research, Ghent, Belgium
8 Department of Biomedical Molecular Biology, Ghent University, Ghent, Belgium
*Corresponding author. Tel: +45 2434 0323; E-mail: mgyrd@sund.ku.dk

© 2022 The Authors. Published under the terms of the CC BY 4.0 license.

*et al*, 2008; Rebsamen *et al*, 2009; Najjar *et al*, 2016; Zhu *et al*, 2018; Muendlein *et al*, 2020). However, the mechanism of RIPK3-mediated inflammatory signaling remains unresolved.

The formation of nondegradative ubiquitin (Ub) chains linked via lysine 63 (K63-Ub) and methionine 1 (M1-Ub) within receptor signaling complexes facilitates the activation of the kinases TAK1 and IKKα/β, which in turn activate MAP kinase signaling and NF-κB signaling to stimulate the expression of pro-inflammatory cytokines and chemokines (reviewed in Hrdinka & Gyrd-Hansen, 2017).

In this study, we identify RIPK1 and RIPK3 as scaffolding kinases that mediate ZBP1-triggered inflammatory signaling independently of cell death. ZBP1-RIPK3-RIPK1 inflammatory signaling is dependent on K63-Ub and M1-Ub assembled by Ub ligases cIAPs and LUBAC but does not require the kinase activity of RIPK1 and RIPK3. Inhibition of caspase activity exposes a RIPK3 kinase activity-mediated inflammatory signaling pathway. Finally, we provide evidence that ZBP1 contributes to the production of cytokines and chemokines during SARS-CoV-2 infection.

## Results

### ZBP1 stimulates inflammatory signaling independently of cell death

ZBP1-induced signaling is mediated by RIPK3 and is dependent on RHIM interactions (Kaiser *et al*, 2008; Rebsamen *et al*, 2009). To investigate the ability of ZBP1 to stimulate inflammatory signaling versus cell death, we generated HT29/Tet-On (TO) cells with doxycycline (Dox)-inducible expression of FLAG-tagged wild-type (WT) ZBP1 ($ZBP1^{WT}$; HT29/TO-$ZBP1^{WT}$) or a ligand-binding-deficient ZBP1 with mutations in the Z-form nucleic acid-binding (Zα) domains ($ZBP1^{Z\alpha1\alpha2mut}$; HT29/TO-$ZBP1^{Z\alpha1\alpha2mut}$; Maelfait *et al*, 2017; Fig 1A). Dox treatment induced the expression of $ZBP1^{WT}$ and $ZBP1^{Z\alpha1\alpha2mut}$ in a dose-dependent manner, albeit $ZBP1^{Z\alpha1\alpha2mut}$ expressed at higher levels than $ZBP1^{WT}$ (Fig 1B). The levels of ZBP1 induced by Dox treatment, in particularly 500 ng/ml Dox, were substantially higher than endogenous ZBP1 induced by IFNβ stimulation (Fig EV1A). Of note, IFNβ predominantly induced expression of a shorter isoform of ZBP1 in HT29 cells.

The cells were generated as puromycin-selected pools and single-cell analysis of ZBP1 expression showed that 500 ng/ml Dox induced ZBP1 expression in 36% of HT29/TO-$ZBP1^{WT}$ cells and 56% of HT29/TO-$ZBP1^{Z\alpha1\alpha2mut}$ cells (Fig 1C). Fifty nanograms per milliliter of Dox induced ZBP1 expression in 16 and 22% of HT29/TO-$ZBP1^{WT}$ and HT29/TO-$ZBP1^{Z\alpha1\alpha2mut}$ cells, respectively (Fig 1C).

In accordance with the ability of ZBP1 to stimulate ligand-dependent cell death (Maelfait *et al*, 2017; Jiao *et al*, 2020; Wang *et al*, 2020), HT29/TO-$ZBP1^{WT}$ cells were more sensitive to Dox-induced cell death than HT29/TO-$ZBP1^{Z\alpha1\alpha2mut}$ cells as determined by SytoxGreen positivity (Figs 1D and EV1B) and viability by CellTitre-Glo assay (Fig EV1C). Treatment of HT29/TO-$ZBP1^{WT}$ cells with 500 ng/ml Dox led to 16 and 22% SytoxGreen-positive cells at 24 and 48 h, respectively, whereas no increase in cell death of HT29/TO-$ZBP1^{Z\alpha1\alpha2mut}$ cells was detected at 24 h. After 48 h of Dox-treatment, 18% of HT29/TO-$ZBP1^{Z\alpha1\alpha2mut}$ were SytoxGreen-positive. Fifty nanograms per milliliter of Dox treatment did not increase cell death of HT29/TO-$ZBP1^{Z\alpha1\alpha2mut}$ cells but increased SytoxGreen-positivity of HT29/TO-$ZBP1^{WT}$ cells to 9% at 48 h. Also, HT29/TO-$ZBP1^{WT}$ cells displayed a loss of viability at 48 and 72 h of treatment with 500 ng/ml Dox while HT29/TO-$ZBP1^{Z\alpha1\alpha2mut}$ cells displayed a much reduced and nonsignificant loss of viability (Fig EV1C). Viability measurements are confounded by proliferation of the surviving cells in the culture, which likely is the reason why no significant loss of viability of HT29/TO-$ZBP1^{Z\alpha1\alpha2mut}$ cells was measured despite an increase in SytoxGreen-positive cells at 48 h of Dox treatment.

The treatment of HT29/TO-$ZBP1^{WT}$ cells with Dox induced only a modest amount of cell death (16%) as compared to treatment with the necroptosis-inducing stimulus TNF, Smac mimetic (LCL161), and zVAD-fmk (hereafter termed TSZ), which led to approx. 80% cell death after 24 h (Fig EV1D). We speculated that this, at least partly, is because 500 ng/ml Dox induced $ZBP1^{WT}$ expression only in 36% of the cells in the population (Fig 1C). In line with this, treatment of monoclonal (clone B9) HT29/TO-$ZBP1^{WT}$ cells with 500 ng/ml Dox led to substantially more cell death (43%) than was observed for the polyclonal cells (Fig 1E). Contrary to previous studies in murine systems (Upton *et al*, 2012; Jiao *et al*, 2020; Wang *et al*, 2020; Zhang *et al*, 2020), cell death induced by ZBP1 in HT29 cells was predominantly apoptosis as the pan-caspase inhibitor zVAD-fmk (zVAD) inhibited cell death and largely restored viability, whereas inhibition of RIPK3 (GSK′872 or GSK′840), RIPK1 (Nec1s), or MLKL (necrosulfonamide; NSA) did not (Fig 1E and F). The combined inhibition of caspases and RIPK3 (zVAD + GSK′872 or GSK′840) or RIPK1 (zVAD + Nec1s) was slightly more protective as compared with zVAD treatment alone, suggesting that caspase inhibition promoted ZBP1-induced necroptosis (Fig 1E and F). Inhibition of RIPK1 or RIPK3 kinase activity prevented TSZ-induced cell death of HT29/TO-$ZBP1^{WT}$ cells, validating that the inhibitors were effective in inhibiting necroptosis (Figs 1G and EV1D).

Next, we investigated the ability of ZBP1 to induce cytokine production. Treatment of HT29/TO-$ZBP1^{WT}$ cells with 50 or 100 ng/ml Dox induced expression of ZBP1 but did not result in loss of viability or cell death within 24 h (Figs 1B, D and E, and EV1C). Based on this, we treated the cells with increasing concentrations of Dox up to 50 ng/ml and measured cytokine production after 24 h. ZBP1 expression stimulated the production of chemotactic cytokines CXCL8 and CXCL1 in a Dox concentration-dependent manner, which was also dependent on the ligand-binding ability of ZBP1 (Fig 1H). Dox-induced expression of $ZBP1^{Z\alpha1\alpha2mut}$ was higher than of $ZBP1^{WT}$, yet the cytokine production was significantly lower, showing that ligand-binding mediated ZBP1 signaling under these conditions (Fig 1H and I). At higher Dox concentrations, both $ZBP1^{WT}$ and $ZBP1^{Z\alpha1\alpha2mut}$ stimulated production of cytokines, indicating that when highly overexpressed ZBP1 induces inflammatory signaling independently of ligand-binding as previously reported (Fig EV1E and F; Maelfait *et al*, 2017). Notably, the level of exogenous ZBP1 in the HT29/TO-ZBP1 cells, both after treatment with 50 ng/ml and 500 ng/ml Dox, was higher than the level of endogenous ZBP1 induced by IFNβ, which we speculate is the reason that ZBP1 signaling responses were stimulated in the absence of virus infection or conditions that upregulate endogenous Z-form nucleic acids (Thapa *et al*, 2016; Jiao *et al*, 2020, 2022; Wang *et al*, 2020; de Reuver *et al*, 2022; Hubbard *et al*, 2022; Zhang *et al*, 2022).


© 2022 The Authors

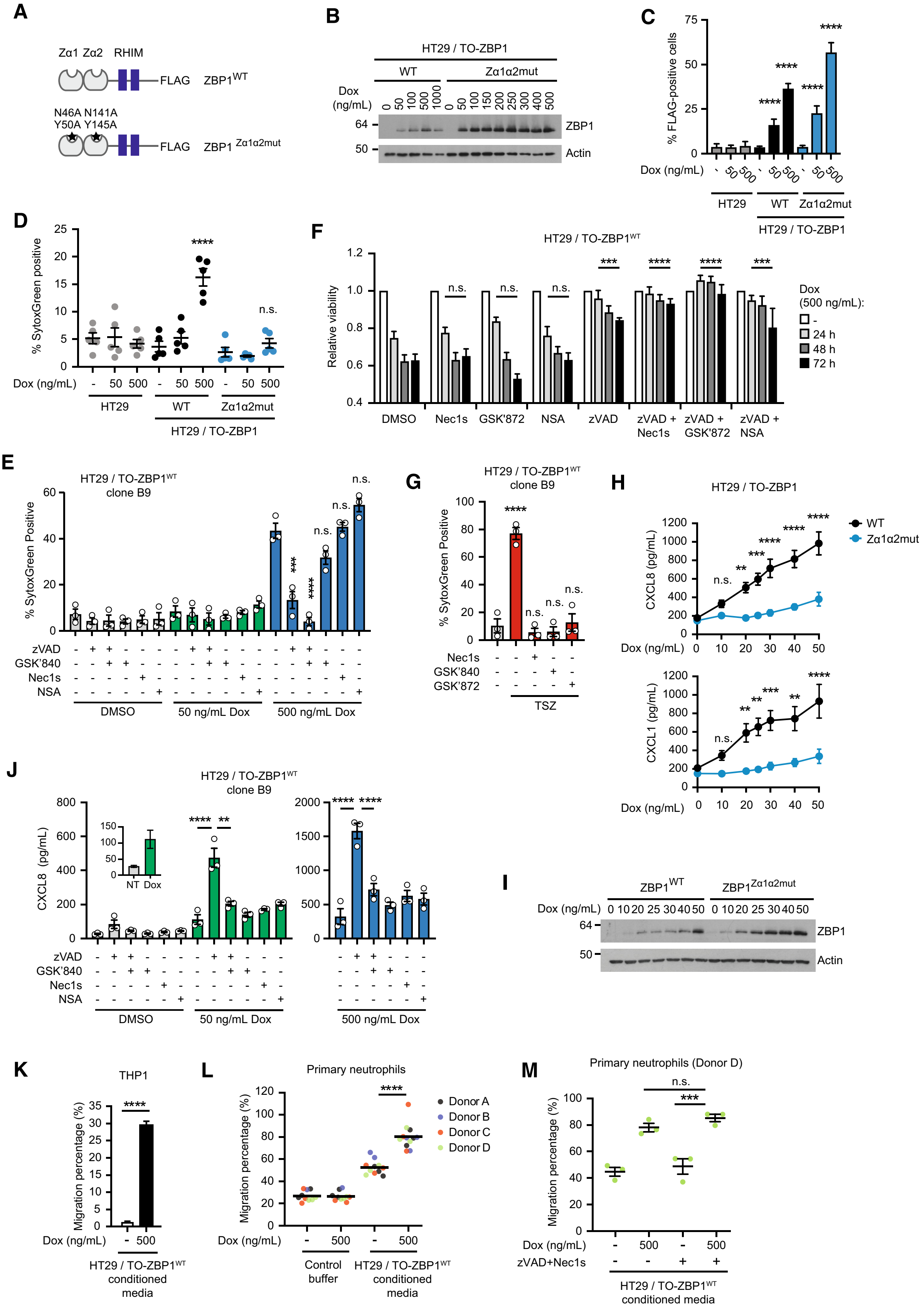
A
Zα1 Zα2 RHIM
FLAG ZBP1WT
N46A N141A
Y50A Y145A
FLAG ZBP1 Zα1α2mut
B
HT29 / TO-ZBP1
WT
Zα1α2mut
Dox (ng/mL)
ZBP1
Actin
C
% FLAG-positive cells
Dox (ng/mL)
HT29
WT
Zα1α2mut
HT29 / TO-ZBP1
D
% SytoxGreen positive
Dox (ng/mL)
HT29
WT
Zα1α2mut
HT29 / TO-ZBP1
F
HT29 / TO-ZBP1WT
Relative viability
Dox (500 ng/mL):
24 h
48 h
72 h
DMSO
Nec1s
GSK'872
NSA
zVAD
zVAD + Nec1s
zVAD + GSK'872
zVAD + NSA
E
HT29 / TO-ZBP1WT clone B9
% SytoxGreen Positive
zVAD
GSK'840
Nec1s
NSA
DMSO
50 ng/mL Dox
500 ng/mL Dox
G
HT29 / TO-ZBP1WT clone B9
% SytoxGreen Positive
Nec1s
GSK'840
GSK'872
TSZ
H
HT29 / TO-ZBP1
WT
Zα1α2mut
CXCL8 (pg/mL)
CXCL1 (pg/mL)
Dox (ng/mL)
I
ZBP1WT
ZBP1Zα1α2mut
Dox (ng/mL)
ZBP1
Actin
J
HT29 / TO-ZBP1WT clone B9
CXCL8 (pg/mL)
NT Dox
zVAD
GSK'840
Nec1s
NSA
DMSO
50 ng/mL Dox
500 ng/mL Dox
K
THP1
Migration percentage (%)
Dox (ng/mL)
HT29 / TO-ZBP1WT conditioned media
L
Primary neutrophils
Donor A
Donor B
Donor C
Donor D
Migration percentage (%)
Dox (ng/mL)
Control buffer
HT29 / TO-ZBP1WT conditioned media
M
Primary neutrophils (Donor D)
Migration percentage (%)
Dox (ng/mL)
zVAD+Nec1s
HT29 / TO-ZBP1WT conditioned media

**Figure 1.**

◀ **Figure 1. ZBP1 stimulates inflammatory signaling independently of cell death.**

A Schematic illustration of WT and Zα1α2-mutant (Zα1α2mut) human ZBP1 inducibly expressed in HT29/TO-ZBP1 cells.

B Western blot analysis of the dose-dependent expression of ZBP1 in HT29/TO-ZBP1$^{WT}$ and HT29/TO-ZBP1$^{Z\alpha1\alpha2mut}$ cells treated with indicated concentrations of Dox for 24 h. Blots are representative of three biological repeats.

C Flow cytometry analysis of ZBP1 expression in HT29/TO-ZBP1 cells treated with Dox for 24 h. FLAG antibody staining was used to determine ZBP1-expressing cells. Data are presented as mean with S.E.M ($n = 5$ biological replicates). Two-way ANOVA and Tukey's multiple comparisons tests were used to test for statistical differences between indicated condition and DMSO-treated condition within each cell line. ****$P < 0.0001$ for all conditions.

D, E Cell death analysis of HT29 and HT29/TO-ZBP1 cells as indicated following 24 h treatment with Dox using SytoxGreen to stain dead cells. Data are presented as mean ± S.E.M ((D) $n = 5$ biological replicates, (E) $n = 3$ biological replicates). Two-way ANOVA and Sidak's multiple comparisons tests were used to test for the statistical differences between indicated condition and untreated condition within each cell line. n.s., not significant ($P \geq 0.05$); ***$P = 0.0004$; ****$P < 0.0001$.

F Relative viability of HT29/TO-ZBP1$^{WT}$ cells treated with 500 ng/ml Dox for up to 3 days in combination with 20 μM zVAD, 10 μM Nec1s, 10 μM GSK′872, and/or 1 μM NSA as indicated was determined by the CellTitre-Glo assay. Values are normalized to untreated wells. Data are plotted as mean with S.E.M. ($n = 4$ biological replicates). Two-way ANOVA and Dunnet's multiple comparisons tests were used to test for statistical differences between indicated condition and DMSO-treated condition. n.s., not significant ($P > 0.99$); ***$P = 0.0006$ for zVAD, $P = 0.0007$ for zVAD+NSA; ****$P < 0.0001$.

G Cell death analysis of HT29/TO-ZBP1$^{WT}$ Clone B9 cells following treatment as indicated using SytoxGreen to stain dead cells. Cells were pretreated with 100 nM LCL161, 20 μM zVAD combined with10 μM GSK′840, 10 μM GSK′872, or 10 μM Nec1s for 1 h followed by treatment with 10 ng/ml TNF. Cell death analysis was carried out at the end of 24 h incubation with all compounds. Data are plotted as mean with S.E.M. ($n = 3$ biological replicates). Repeated measures one-way ANOVA and Sidak's multiple comparisons tests were used to test for statistical differences between indicated condition and untreated control. ****$P < 0.0001$; n.s., not significant ($P > 0.4$).

H Cytokine concentration in the culture media of HT29/TO-ZBP1 cells treated for 24 h with the indicated Dox concentrations or vehicle. Data are presented as mean with S.E.M ($n = 4$ biological replicates). Two-way ANOVA and Sidak's multiple comparisons *t*-test were used to test for the statistical differences between the two cell lines at each concentration. n.s., not significant ($P > 0.5$); **$P = 0.0035$ for CXCL8 at 20 ng/ml, $P = 0.0067$ for CXCL1 at 20 ng/ml, $P = 0.0020$ at 50 ng/ml, $P = 0.0013$ at 40 ng/ml; ***$P = 0.0004$ for CXCL8 at 25 ng/ml, $P = 0.0008$ at 30 ng/ml; ****$P < 0.0001$.

I Western blot analysis of cells from the same wells as used in (H) for ZBP1 expression levels, representative of four biological replicates.

J CXCL8 concentration in the culture media from the experiment described in (E). Data are presented as mean with S.E.M. ($n = 3$ biological replicates). Two-way ANOVA tests and Sidak's multiple comparisons test were used to test for statistical differences between indicated conditions. **$P = 0.0079$; ****$P < 0.0001$.

K Transwell migration of THP1 cells toward conditioned media from HT29/TO-ZBP1$^{WT}$ cells treated with DMSO or 500 ng/ml Dox for 24 h. Data are presented as mean with S.E.M. ($n = 3$ biological replicates of conditioned media). An unpaired *t*-test was used to test for statistical differences between indicated conditions. ****$P < 0.0001$.

L Transwell migration of primary neutrophils toward conditioned media from HT29/TO-ZBP1$^{WT}$ cells treated with DMSO or 500 ng/ml Dox for 24 h, or HT29 chemotaxis buffer (control buffer) containing equal volume and amount of DMSO or Dox as the conditioned media. Data are presented as individual values with grand mean of the migrated percentage induced by control buffer containing DMSO or Dox ($n = 10$ biological replicates), or conditioned media from DMSO-treated cells ($n = 11$ biological replicates) or Dox-treated cells ($n = 12$ biological replicates), where the number of biological replicates is defined by the total number of independent cell cultures tested on primary neutrophils from four donors. An unpaired *t*-test was used to test for statistical differences between indicated conditions. ****$P < 0.0001$.

M Transwell migration of primary neutrophils towards conditioned media from HT29/TO-ZBP1$^{WT}$ cells treated with 0 or 500 ng/ml Dox in combination with DMSO or 20 μM zVAD +10 μM Nec1s for 24 h. Data are presented as individual values with mean and S.E.M. ($n = 3$ biological replicates of conditioned media). One-way ANOVA and Sidak's multiple comparisons test were used to test for statistical differences between indicated conditions. n.s. = not significant ($P = 0.4129$); ***$P = 0.0004$.

Source data are available online for this figure.

To investigate directly if cytokine production by ZBP1 was independent of cell death or might occur as a consequence of low levels of cell death, cytokine production and cell death was measured from the same wells following treatment of HT29/TO-ZBP1$^{WT}$ B9 monoclonal cells with the indicated inhibitors and Dox for 24 h (Figs 1E and J, and EV1G). In line with the data from the polyclonal cells, the induction of ZBP1 expression by 50 ng/ml Dox stimulated cytokine production without detectable cell death, whereas 500 ng/ml Dox led to cytokine production and cell death (Figs 1E and J, and EV1G). Strikingly, inhibition of caspases by zVAD augmented cytokine production both in cells treated with 50 ng/ml Dox and in cells treated with 500 ng/ml Dox where zVAD inhibited cell death (Figs 1E and J, and EV1G). Treatment with zVAD in combination with the RIPK3 kinase inhibitor GSK′840 completely prevented Dox-induced cell death and reduced CXCL8 largely to the level induced by Dox-treatment without zVAD (Figs 1E and J, and EV1G). These data strongly suggest that cell death is not responsible for ZBP1-induced cytokine production. Notably, cells were plated at a 4-fold lower density than used for other cytokine measurements to accurately determine cell death. This resulted in approx. 10-fold lower cytokine concentrations in the media both at baseline and after Dox-treatment.

Similarly, treatment of HT29/TO-ZBP1$^{WT}$ polyclonal cells with 50 ng/ml Dox in the presence of zVAD led to enhanced CXCL8 production, which was suppressed by GSK′872, Nec1s, and NSA (Fig EV1H). Notably, GSK′872 reduced the Dox-induced ZBP expression under these conditions, which likely contributed to the suppression of CXCL8 levels. Since necroptosis inhibitors suppressed the zVAD-enhanced cytokine levels and RIPK3 inhibition inhibited the low level of cell death after treatment with Dox and zVAD, we speculate that the increased cytokine levels may originate from RIPK3 kinase activity- and MLKL-dependent inflammatory signaling in necroptotic cells as previously reported (Zhu *et al*, 2018; Orozco *et al*, 2019) and/or from secondary responses to released cellular contents from necroptotic cells. However, further experiments will be needed to delineate the mechanism by which caspases restrict ZBP1 inflammatory signaling.

In addition to CXCL8 and CXCL1, we found that ZBP1 expression in HT29 cells stimulated the secretion of the chemokines CXCL10, CCL20, CXCL7, and other proinflammatory mediators (Fig EV2A and B). This suggests that ZBP1 signaling may stimulate the chemoattraction of neutrophils and other immune cells. Indeed, conditioned media from Dox-treated HT29/TO-ZBP1$^{WT}$ cells

 © 2022 The Authors

stimulated the chemotactic migration of THP1 monocytic cells, neutrophil-like differentiated HL60 cells and primary human donor neutrophils (Figs 1K and L, and EV2C and D). HT29/TO-ZBP1$^{WT}$ cells were treated with 500 ng/ml Dox as this concentration induced higher levels of chemokines in the media than treatment with 50 ng/ml Dox. To determine if cell death of HT29/TO-ZBP1$^{WT}$ cells after Dox treatment might contribute to the release of chemotactic factors to the media, cells were treated with zVAD and Nec1s in combination with Dox. However, the chemotactic migration of primary donor neutrophils was unaffected by treatment of the HT29/TO-ZBP1$^{WT}$ cells with zVAD and Nec1s, indicating that neutrophil migration was stimulated by ZBP1-dependent inflammatory signaling and not by cell death (Fig 1M).

### ZBP1-induced inflammatory signaling is mediated by the scaffolding function of RIPK1 and RIPK3

To gain insights into the mechanism underpinning ZBP1-induced inflammatory signaling, HT29 cells were treated with kinase inhibitors in combination with Dox-induced expression of ZBP1. Inhibition of RIPK1 or RIPK3 kinase activity, or inhibition of MLKL had no or a modest effect on ZBP1-induced cytokine production (Figs 1J and 2A, and EV3A, and EV1G and H) or cell death (Figs 1E and F, and 2B). Also, inhibition of RIPK3, RIPK1 or MLKL did not prevent the ZBP1-stimulated chemotactic migration of neutrophils (Fig 2C).

Contrary to the inhibition of kinase activity, siRNA-mediated depletion of RIPK3 or genetic knockout of RIPK1 (RIPK1 KO) suppressed ZBP1-induced inflammatory signaling, indicating that RIPK3 and RIPK1 mediate ZBP1 signaling independently of their kinase activity (Fig 2D and E). Surprisingly, RIPK1 KO cells were also completely protected from ZBP1-induced cell death despite expressing ZBP1 at similar levels as RIPK1 WT cells (Fig 2E and F). The requirement of RIPK1 in ZBP1 signaling was tested in two additional RIPK1 KO clones with similar results (Fig EV3B and C). This shows that RIPK1 in HT29 cells facilitates ZBP1 signaling as opposed to previous studies in murine systems where RIPK1 is reported to suppress activation of RIPK3 by ZBP1 (Lin *et al*, 2016; Newton *et al*, 2016).

The kinases TAK1 and IKK are essential for activation of NF-κB and cytokine production by immune receptors. We therefore tested if these kinases mediate ZBP1-dependent inflammatory signaling. Indeed, ZBP1-induced cytokine production and neutrophil migration were blocked by the TAK1 inhibitor 5z-7-oxozeaenol (TAK1i; Wu *et al*, 2013) and IKK inhibitors (IKKi; Waelchli *et al*, 2006; Christopher *et al*, 2007; Fig 2A and C). Of note, TAK1i treatment resulted in reduced viability of HT29/TO-ZBP1$^{WT}$ cells, possibly due to its apoptosis-promoting activity (Singh *et al*, 2012; Fig 2B). Together, this shows that ZBP1 in HT29 cells induces TAK1 and IKK-mediated inflammatory signaling that relies on RIPK3 and RIPK1 as scaffolds rather than their kinase activity.

To investigate the role of RIPK3 in ZBP1 signaling in another cell system, ZBP1 was transiently expressed in HEK293FT cells with stable expression of RIPK3 (RIPK3$^{FL}$) or a C-terminal truncated RIPK3 lacking the RHIM (RIPK3$^{\Delta C}$). The RIPK3 variants were fused to two copies of the homodimerization domain B (DmrB) from the FK506-binding protein F36V mutant (Clackson *et al*, 1998; termed RIPK3-2xFV). The FV domains were added to enable chemical oligomerization of RIPK3, which we employed in later experiments.

In line with previous reports (Kaiser *et al*, 2008; Rebsamen *et al*, 2009), ZBP1$^{WT}$-induced NF-κB activity was increased in cells expressing RIPK3$^{FL}$ but not in cells expressing RIPK3$^{\Delta C}$, which lacks the RHIM (Fig 2G). The ability of ZBP1 to activate NF-κB in cells expressing RIPK3$^{FL}$ was mediated partly by ligand-binding since ZBP1$^{Z\alpha1\alpha2mut}$ induced less NF-κB activity than ZBP1$^{WT}$ although the expression levels similar (Fig 2G). The inhibition of RIPK3, RIPK1, or MLKL did not inhibit ZBP1$^{WT}$-induced NF-κB activity in the RIPK3$^{FL}$ -expressing HEK293FT cells, showing that ZBP1-induced inflammatory signaling also in this cell system is mediated by ligand-binding and does not require the kinase activity of RIPK1 or RIPK3, but relies on RIPK3 as a scaffold (Fig 2H).

### K63-Ub and M1-Ub facilitate ZBP1-induced inflammatory signaling

RIP kinases function as scaffolds by serving as substrates for non-degradative ubiquitination in inflammatory signaling (Ea *et al*, 2006; Hasegawa *et al*, 2008; Damgaard *et al*, 2012; Hrdinka *et al*, 2018), which prompted us to investigate ubiquitination events after ZBP1 induction. FLAG-tagged ZBP1 was immunoprecipitated from Dox-treated HT29/TO-ZBP1$^{WT}$ cells. As expected, RIPK3 and RIPK1 were both co-purified with ZBP1 (Fig 3A; Kaiser *et al*, 2008; Rebsamen *et al*, 2009). Interestingly, ZBP1 also co-purified high molecular weight (MW) Ub-conjugates, which was confirmed by treatment of the immunoprecipitated material with the deubiquitinase USP21 (Fig 3A). Enrichment of endogenous Ub-conjugates by GST-1xUBA (Fiil *et al*, 2013; Hrdinka *et al*, 2016) revealed that ZBP1 expression increased the ubiquitination of RIPK1 and of ZBP1 itself, whereas ubiquitination of RIPK3 was not detected under these conditions (Fig 3B). Further, enrichment of K63- and M1-Ub by linkage-selective Ub binders (SUBs; Fiil *et al*, 2013; Hrdinka *et al*, 2016) suggests that RIPK1 and ZBP1 were both modified by K63-Ub, whereas M1-Ub appeared to predominantly accumulate on ZBP1 albeit Ub-modified RIPK1 was enriched by the M1-SUB in one of three experiments (Figs 3C and EV3D). Interestingly, Dox-induced ubiquitination of ZBP1 was not detected in RIPK1-KO cells, which suggests that RIPK1 or the ubiquitination of RIPK1 is prerequisite for ubiquitination of ZBP1 (Fig 3D). Unmodified forms of RIPK1 and ZBP1 also co-purified with GST-1xUBA and the SUBs, which likely is a result of Ub-independent protein–protein interactions with the recombinant Ub-binding protein (GST-1xUBA/SUB) or with other Ub-modified proteins in the sample as previously observed (Fiil *et al*, 2013; Hrdinka *et al*, 2016).

Binding of SUBs to the corresponding Ub chain linkage can block the signaling capability of the Ub chain linkage in cells (Sims *et al*, 2012; van Wijk *et al*, 2012; Fiil *et al*, 2013; Hrdinka *et al*, 2016). To test if K63-Ub and M1-Ub contribute functionally to ZBP1-induced signaling, K63-SUB and M1-SUB were co-expressed with ZBP1 in HEK293FT/RIPK3-2xFV cells. Expression of either K63-SUB or M1-SUB prevented ZBP1-induced NF-κB activity (Fig 3E). Also, transient expression of OTULIN, which cleaves M1-Ub, or CYLD, which preferentially cleaves K63- and M1-Ub, inhibited ZBP1-induced NF-κB activity in HEK293FT/RIPK3-2xFV cells (Fig 3F and G). Expression of catalytically inactive OTULIN (C129A) or CYLD (C601A) did not inhibit ZBP1-induced NF-κB activity (Fig 3F and G). Collectively, this shows that K63-Ub and M1-Ub both contribute to ZBP1-RIPK3-dependent inflammatory signaling.

© 2022 The Authors 

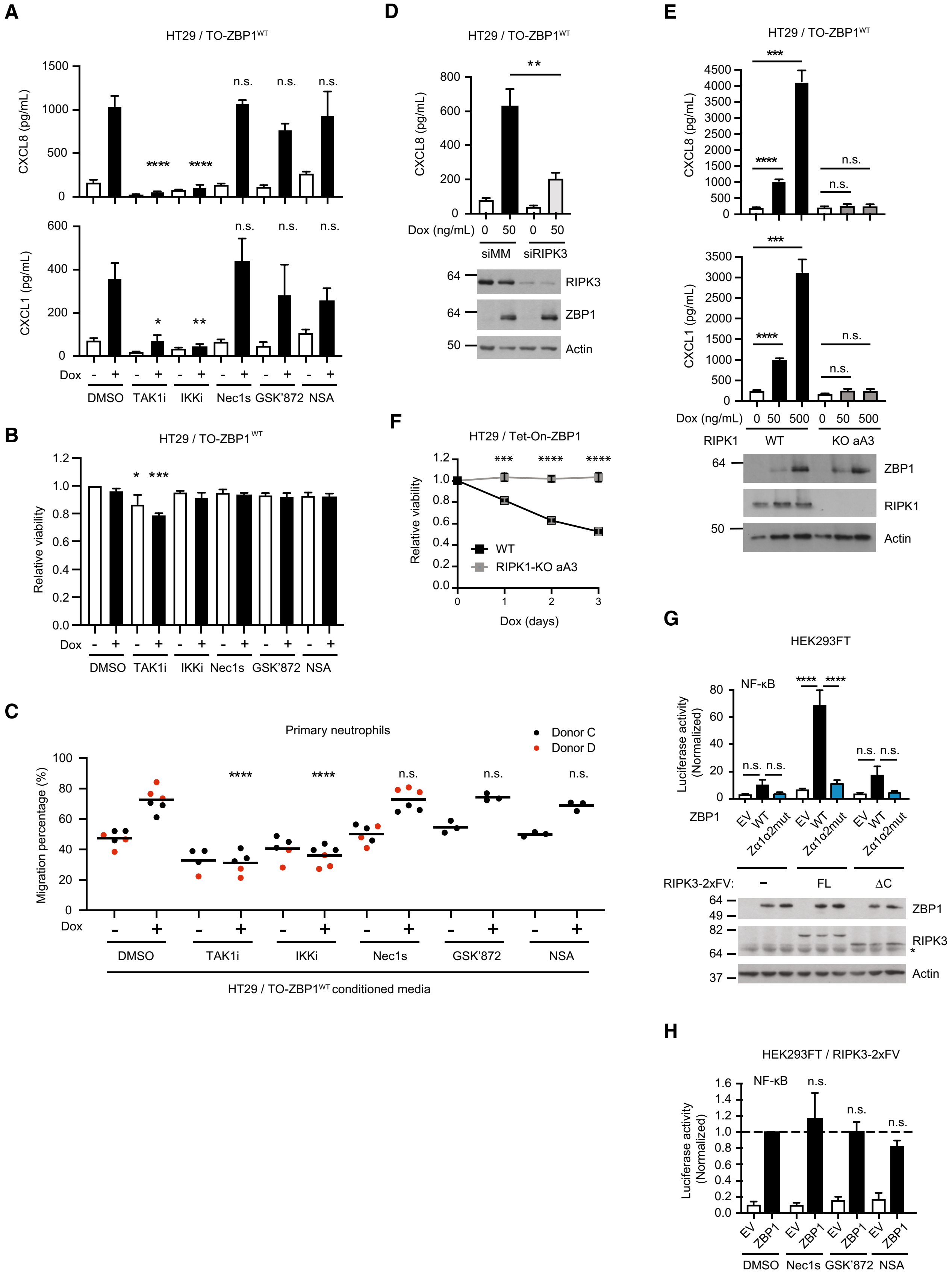


Figure 2.

 © 2022 The Authors

**Figure 2. ZBP1-induced inflammatory signaling requires RIPK3 and RIPK1 but not their kinase activity.**

A Cytokine concentration in the culture media of HT29/TO-ZBP1$^{WT}$ cells treated with 0 or 50 ng/ml Dox in combination with indicated inhibitors for 24 h. TAK1i, 1 μM 5z-7-oxozeaenol. IKKi, 1 μM IKK Inhibitor VII + 5 μM IKK Inhibitor XII. Nec1s, 10 μM. GSK′872, 10 μM. NSA, 1 μM. Data are presented as mean with S.E.M. ($n = 3$ biological replicates). One-way ANOVA and Sidak's multiple comparisons tests were used to test for statistical differences between Dox-induced samples pre-treated with DMSO and corresponding inhibitor. n.s. = not significant ($P > 0.2$); *$P = 0.0104$; **$P = 0.0041$; ****$P < 0.0001$.

B Relative viability of HT29/TO-ZBP1$^{WT}$ cells treated with the same conditions as in (A). Data are plotted as mean with S.E.M. ($n = 4$ biological replicates). One-way ANOVA and Sidak's multiple comparisons tests were used to test for statistical differences between all conditions and DMSO-treated condition, with significantly different conditions indicated. *$P = 0.023$; ***$P = 0.0002$.

C Transwell migration of primary neutrophils toward conditioned media from HT29/TO-ZBP1$^{WT}$ cells treated with 0 or 500 ng/ml Dox in combination with indicated inhibitors for 24 h. Data are presented as individual values with grand mean ($n = 3$ biological replicates of conditioned media from GSK′872 and NSA-treated wells, $n = 6$ biological replicates of conditioned media from other conditions). Two-way ANOVA and Sidak's multiple comparisons test were used to test for statistical differences between indicated conditions. ****$P < 0.0001$; n.s. = not significant ($P > 0.96$).

D CXCL8 concentration in the culture media of HT29/TO-ZBP1$^{WT}$ cells transfected with siRNA targeting mismatch sequence (MM) or RIPK3 and treated with Dox at indicated concentrations for 24 h. Data are presented as mean with S.E.M. ($n = 4$ biological replicates). Unpaired $t$-tests were used to test for statistical differences between indicated conditions. **$P = 0.0079$. Cell lysates from one biological replicate were analyzed by Western blotting.

E Cytokine concentration in the culture media of HT29/TO-ZBP1$^{WT}$ or HT29/RIPK1-KO clone aA3/TO-ZBP1$^{WT}$ cells treated with 0, 50 or 500 ng/ml Dox. data is plotted as mean with S.E.M. ($n = 6$ biological replicates). Brown-Forsythe and Welch ANOVA tests and Dunnet's T3 multiple comparisons test were used to test for statistical differences between indicated conditions. Cell lysates were analyzed by Western blotting for ZBP1 levels. Blots are representative of six biological replicates. n.s. = not significant ($P > 0.2$); ***$P = 0.0003$ for CXCL8, $P = 0.0008$ for CXCL1; ****$P < 0.0001$.

F Relative viability of HT29/TO-ZBP1$^{WT}$ and HT29/RIPK1-KO clone aA3/TO-ZBP1$^{WT}$ cells treated with 500 ng/ml Dox for up to 3 days. Values are normalized to day 0 of treatment within each cell line. Data are plotted as mean with S.E.M. ($n = 3$ biological replicates). Two-way ANOVA and Sidak's multiple comparison tests were used to test for statistical differences between WT and RIPK1-KO cells at the indicated time points after Dox treatment. ***$P = 0.0003$; ****$P < 0.0001$.

G, H NF-κB activity in HEK293FT cells with and without stable expression of full-length (FL) RIPK3-2xFV or RIPK3ΔC-2xFV (ΔC) transfected with dual luciferase reporters and ZBP1 or empty vector (EV) as indicated. In (H), inhibitors were added immediately after transfection: Nec1s, 10 μM. GSK′872, 10 μM. NSA, 1 μM. Reporter activities were measured 24 h after transfection. Data are presented as mean with S.E.M ($n = 3$ biological replicates). One-way ANOVA and Sidak's multiple comparisons test were used to test for statistical differences between indicated conditions. n.s. = not significant ($P > 0.05$); ****$P < 0.0001$. (G) Cell lysates were analyzed by Western blotting. Blots are representative of three biological replicates. Asterisk indicates background signals of the antibody.

Source data are available online for this figure.

### cIAP1 and LUBAC regulate ZBP1-induced inflammatory signaling

The involvement of M1-Ub implied that LUBAC contributes to ZBP1-RIPK3-dependent inflammatory signaling. Indeed, siRNA-mediated knockdown of HOIP, the catalytic subunit of LUBAC, in HEK293FT/RIPK3-2xFV cells substantially impaired ZBP1-induced NF-κB activity (Fig 4A). K63-Ub assembled by cIAPs facilitates the recruitment of LUBAC to the TNF receptor signaling complex I (Haas *et al*, 2009). To investigate if cIAPs are involved in ZBP1 signaling, cells were treated with the Smac mimetic Compound A (CpA) to deplete cIAPs (Vince *et al*, 2007). CpA inhibited ZBP1-induced NF-κB activity in HEK293FT/RIPK3-2xFV cells and Dox-induced cytokine production in HT29/TO-ZBP1$^{WT}$ cells (Fig 4B and C). This suggests that cIAPs and LUBAC facilitate ZBP1 signaling by conjugating K63-Ub and M1-Ub, respectively.

Next, we analyzed the ZBP1 complex from Dox-treated HT29/TO-ZBP1$^{WT}$ cells by immunoprecipitation of FLAG-ZBP1. This showed that ZBP1, in addition to RIPK1 and RIPK3, co-immunoprecipitated K63-Ub and M1-Ub, cIAP1, LUBAC (HOIP, HOIL-1, SHARPIN), the LUBAC-associated DUBs OTULIN and CYLD, TAK1, and IKKβ (Fig 4D and E). This suggests that ZBP1 forms a pro-inflammatory receptor signaling complex that, akin to other immune receptor complexes, consists of receptor-associated adaptor kinases, K63- and M1-Ub ligases and DUBs, and the ubiquitin-dependent kinases TAK1 and IKKβ.

To determine the role of RIPK1 in the ZBP1 complex, we compared the Dox-induced ZBP1 complex in WT and RIPK1-KO HT29 cells. Compared with WT cells, RIPK1-KO cells showed reduced association of RIPK3 with ZBP1, in particular phosphorylated RIPK3, suggesting that RIPK1 stabilizes the ZBP1–RIPK3 interaction (Fig 4F). There was also a reduction in Ub-conjugates co-purified with ZBP1 in RIPK1 KO cells (Fig 4F), consistent with the observation that RIPK1 itself is ubiquitinated in response to ZBP1 and that RIPK1 is needed for ZBP1 ubiquitination (Fig 3B and D). Contrary to our expectation, the association of LUBAC, cIAP1, CYLD, and OTULIN with ZBP1 was not reduced in RIPK1-KO cells as compared with WT cells (Fig 4F). The genetic mutations in *RIPK1* in the RIPK1 KO clone used in the experiment cause a frameshift and introduction of a premature stop codon within the kinase domain, eliminating the RHIM and death domain (Table EV1). This excluded the possibility of RIPK1 death domain-mediated recruitment of the ubiquitin machinery, and implies that LUBAC, cIAP1, CYLD, and OTULIN can be recruited to ZBP1 independently of RIPK1 through unresolved mechanisms. However, RIPK1 is required for cIAP1 and LUBAC to facilitate productive signaling (Fig 2E).

In addition to the ubiquitin machinery, immunoprecipitation of ZBP1 showed that both p43/41-caspase-8 and its regulatory protein p43-cFLIP$_L$ co-immunoprecipitated with ZBP1 in Dox-treated HT29/TO-ZBP1$^{WT}$ cells in a RIPK1-dependent manner (Fig 4F). p43/41-caspase-8 and p43-cFLIP$_L$ are generated through formation of a caspase-8:cFLIP$_L$ heterodimer and proteolytic processing by caspase-8. The resultant p43/41-caspase-8:p43-cFLIP$_L$ heterodimer is catalytically active toward the necroptosis-regulatory proteins RIPK1, RIPK3, and CYLD to suppress their activity (Micheau *et al*, 2002; Feng *et al*, 2007; Oberst *et al*, 2011; O'Donnell *et al*, 2011; Newton *et al*, 2019). The association of p43/41-caspase-8 and p43-cFLIP$_L$ with ZBP1 implies that RIPK3 activity is suppressed by caspase-8 in the ZBP1 complex, which may explain our observation that zVAD increased CXCL8 production in a manner dependent on RIPK3 kinase activity (Fig 1J). Curiously, full-length caspase-8 was co-purified with ZBP1 in the absence of RIPK1, which was unexpected since the mutations introduced in *RIPK1* eliminated the RHIM and death domain in RIPK1 (Table EV1). Further investigation will be needed to resolve how caspase-8 is recruited to ZBP1.

© 2022 The Authors 

### Chemical oligomerization of RIPK3 induces inflammatory signaling

RIPK3 oligomerization in murine cells induces necroptosis and NF-κB activation (Yatim *et al*, 2015). To investigate if oligomerization of human RIPK3 induces ubiquitin-dependent inflammatory signaling through a similar pathway as human ZBP1, we employed the RIPK3-2xFV oligomerization system, which allows for acute and controlled activation of RIPK3 by B/B homodimerizer AP20187 (hereafter referred to as dimerizer; Clackson *et al*, 1998; Orozco *et al*, 2014; Yatim *et al*, 2015; Rodriguez *et al*, 2016). For this, RIPK3$^{FL}$-2xFV and RIPK3$^{\Delta C}$-2xFV were stably expressed in HT29, HCT116, U2OS/NOD2, and HEK293FT cells (Fig 5A). Dimerizer-induced RIPK3 oligomerization in

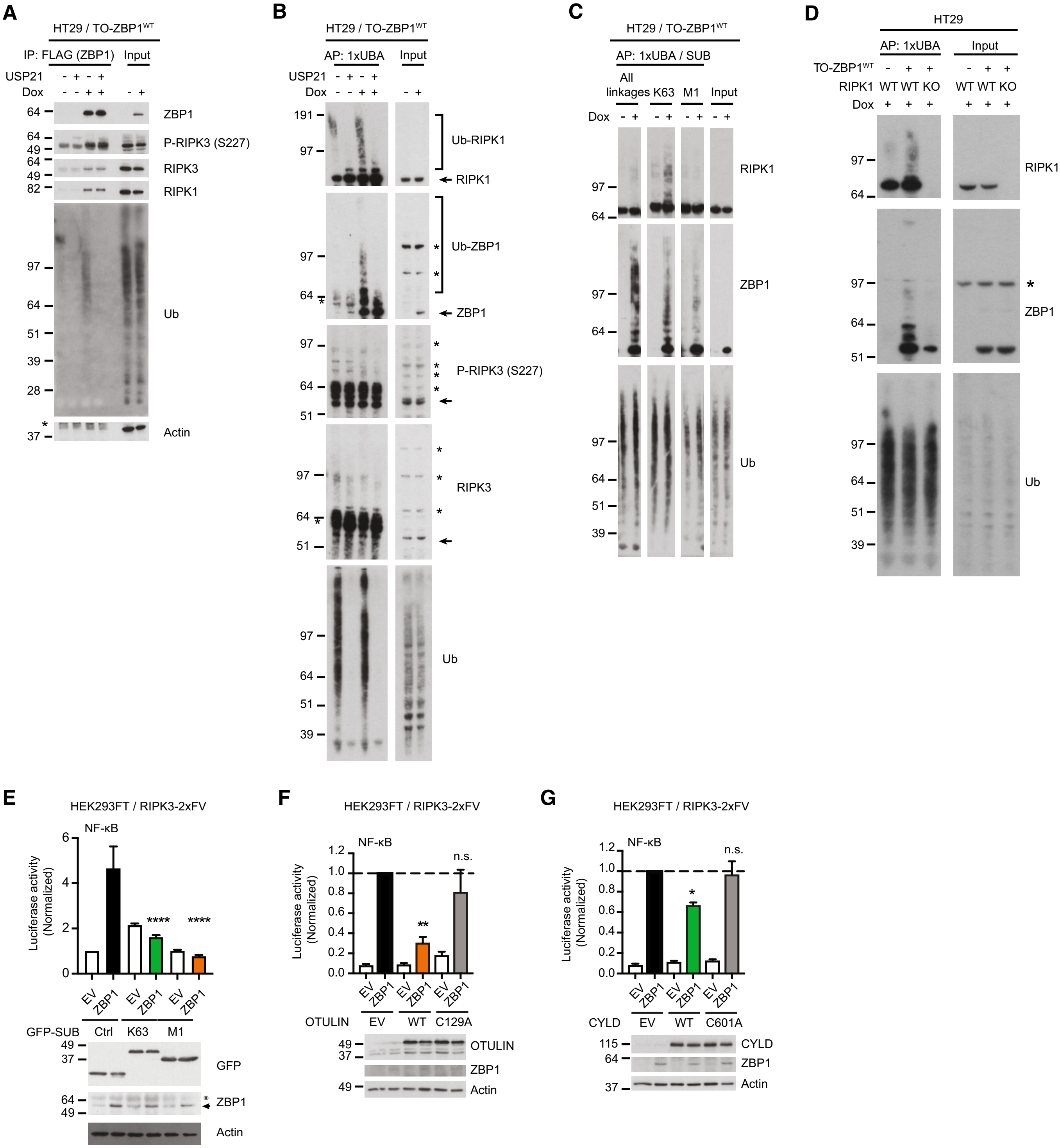


**Figure 3.**


© 2022 The Authors

**Figure 3. K63-Ub and M1-Ub facilitate ZBP1 inflammatory signaling.**

A Western blot analysis of anti-FLAG (ZBP1) immunoprecipitation from HT29/TO-ZBP1$^{WT}$ cells treated with 0 or 500 ng/ml Dox for 16 h. Blots are representative of three biological replicates. Immunoprecipitated material was treated or not with USP21 (1 μM) for deubiquitination. Input loaded was 5% for ZBP1 and 1% for co-immunoprecipitants. Asterisk indicates antibody heavy chain signal in IP samples.

B Enrichment of Ub-conjugates by GST-1xUBA for analysis of ubiquitination status of RIPK1, ZBP1, and RIPK3 in HT29/TO-ZBP1$^{WT}$ cells treated with 0 or 500 ng/ml Dox for 16 h. Blots are representative of at least three biological replicates. After enrichment, samples were treated or not with USP21 (1 μM) for deubiquitination. Asterisk indicates unspecific bands. Arrows indicate RIPK3 signal.

C Enrichment of Ub conjugates for analysis of the ubiquitination status of RIPK1 and ZBP1 using GST-1xUBA or linkage-specific SUBs in HT29/TO-ZBP1$^{WT}$ cells treated with 0 or 500 ng/ml Dox for 16 h. See Fig EV3D for two biological replicates.

D Enrichment of Ub conjugates by GST-1xUBA for analysis of ubiquitination status of RIPK1 and ZBP1 in HT29, HT29/TO-ZBP1$^{WT}$, and HT29/RIPK1-KO aA3/TO-ZBP1$^{WT}$ cells treated with 500 ng/ml Dox for 16 h. Blots are representative of four biological replicates. Asterisk indicates antibody background signal.

E NF-κB activity in HEK293FT/RIPK3-2xFV cells transfected with dual luciferase reporters, ZBP1 or EV, and GFP (Ctrl) or GFP-tagged SUBs as indicated. Luciferase reporter activities were measured 24 h after transfection and normalized to GFP + EV-transfected condition. Data are plotted as mean with S.E.M. ($n = 4$ biological replicates). One-way ANOVA and Sidak's multiple comparisons tests were used to test for statistical differences between the indicated condition and ZBP1/GFP-transfected condition. ****$P < 0.0001$. Cell lysates were analyzed by Western blotting to determine expression of ZBP1, GFP, or GFP-tagged SUBs and Actin. Blots are representative of three biological replicates.

F, G NF-κB activity in HEK293FT/RIPK3-2xFV cells transfected with dual luciferase reporters, ZBP1 or EV, and variants of CYLD or OTULIN. Luciferase reporter activities were measured 24 h after transfection and normalized to ZBP1/EV-transfected cells. Data are plotted as mean with S.E.M. Multiple Welch $t$-tests were used to test for statistical differences between the indicated condition and the ZBP1/EV-transfected condition. (F) $n = 4$ biological replicates. **$P = 0.0012$, n.s. = not significant ($P = 0.2342$). (G) $n = 3$ biological replicates. *$P = 0.0137$; n.s. = not significant ($P = 0.8374$). Cell lysates were analyzed by Western blotting to determine expression of ZBP1, CYLD or OTULIN, and Actin. Blots are representative of (F) four or (G) three biological replicates.

Source data are available online for this figure.

HCT116 cells stimulated inflammatory signaling after 30–60 min as determined by phosphorylation of the NF-κB subunit RelA/p65 and the mitogen-activated protein kinase (MAPK) p38 (Fig 5B), increased expression of cytokine genes after 2–3 h (Fig 5C), and CXCL8 secretion at 24 h (Fig 5D). The inflammatory signaling response was not detected in cells expressing RHIM-truncated or mutated variants of RIPK3 (Fig 5B–D). Contrary to murine cells where oligomerization of RIPK3 induces both necroptosis and inflammatory signaling (Orozco *et al*, 2014; Yatim *et al*, 2015), human RIPK3 oligomerization did not result in detectable loss of viability of HCT116 cells at 24 h and only slightly increased the percentage of SytoxGreen positive cells (Fig 5E and F). TSZ treatment of the HCT116/RIPK3-2xFV cells caused only very modest cell death (4%; Fig 5F), and we therefore tested the response of HT29 cells to RIPK3 oligomerization since these cells were sensitive to TNF-induced necroptosis (Fig EV4A and B). However, RIPK3 oligomerization in HT29 cells did not lead to a detectable loss of viability at 24 h although it induced prominent MAPK and NF-κB signaling and CXCL8 production (Fig EV4C–F). RIPK3 oligomerization in U2OS/NOD2 and HEK293FT cells led activation of NF-κB and MAPK pathways with similar kinetics as observed in HCT116 and HT29 cells (Fig EV4G–I). Taken together, RIPK3 oligomerization induced inflammatory signaling in the absence of detectable cell death.

Like ZBP1-induced inflammatory signaling in HT29 cells, RIPK3 oligomerization-induced inflammatory signaling was attenuated by chemical inhibition of TAK1 and IKK, by CpA-induced depletion of cIAPs and by deletion of HOIP (Figs 5G–J and EV4J and K). Also, the expression of K63-SUB and M1-SUB to functionally inhibit K63-Ub and M1-Ub, respectively, suppressed the dimerizer-induced expression of *CXCL8* (Fig 5K). This suggests that cIAPs and LUBAC facilitate inflammatory signaling downstream of RIPK3 oligomerization by conjugating K63-Ub and M1-Ub. Since CpA antagonizes the function also of the IAP family member X-linked IAP (XIAP) in NOD2-RIPK2 signaling when used at high concentrations (1 μM and above; Damgaard *et al*, 2013), we tested if XIAP might contribute to RIPK3 signaling. However, RIPK3 oligomerization-induced inflammatory signaling was comparable in WT and XIAP-deficient HCT116 cells (Fig EV4L).

zVAD treatment and shRNA-mediated knockdown of caspase-8 in HCT116/RIPK3-2xFV cells enhanced the expression of chemokines following dimerizer treatment, which was suppressed by co-treatment with GSK′872, Nec1s or NSA (Fig 5L and M). This shows that caspase-8 negatively regulates RIPK1/3 kinase activity- and MLKL-mediated inflammatory signaling after RIPK3 oligomerization. Notably, inhibition of RIPK1 or RIPK3 kinase activity, or MLKL also partially inhibited dimerizer-induced expression of cytokines without inhibition of caspase-8 activity (Fig 5L), suggesting that enforced RIPK3 oligomerization stimulates both RIPK3 kinase activity-dependent and -independent signaling. While dimerizer treatment of HCT116/RIPK3-2xFV cells induced only a marginal increase in cell death at 24 h (2%) and gene expression was measured after 3 h of treatment, our data does not exclude that the suppression of cytokine expression by GSK′872, Nec1s, and NSA is a result of inhibition of necroptosis of a small percentage of cells (Orozco *et al*, 2019).

In summary, oligomerization of human RIPK3 shows that RIPK3 can stimulate inflammatory signaling mediated by K63- and M1-Ub, cIAPs, LUBAC, and the kinases TAK1 and IKK. This supplements our data showing that human ZBP1 can induce RIPK3-mediated inflammatory responses independently of cell death and suggests that RIPK3 may promote inflammatory signaling in the context of other immune receptors.

## ZBP1 contributes to SARS-CoV-2-induced cytokine production

Publicly available RNA-sequencing datasets (Blanco-Melo *et al*, 2020a, Data ref: Blanco-Melo *et al*, 2020b) show a substantial increase in *ZBP1* expression in postmortem patient lung biopsies compared to healthy controls (Fig EV5A). Analysis of a single-cell RNA-sequencing dataset (Ren *et al*, 2021a, Data ref: Ren *et al*, 2021b) showed that ZBP1 expression was significantly higher

© 2022 The Authors 

in COVID-19 patients in the progressive disease stage than those in the convalescent stage or healthy controls and a positive correlation between virus load and the expression of ZBP1 and various cytokines in virus-positive cells from bronchoalveolar lavage fluid (BALF) and sputum samples (Fig EV5B and C). This prompted us to investigate if ZBP1 has a role in cytokine production in response to SARS-CoV-2 virus infection.

Calu-3 human lung epithelial cell line was used as they are readily infected by SARS-CoV-2 and are widely used in studies of SARS-CoV-2-induced intracellular signaling (Li *et al*, 2020, 2021; Wyler *et al*, 2021). Also, Calu-3 cells had readily detectable levels of RIPK1, RIPK3, and MLKL, and can be induced to express ZBP1 by IFNβ (Fig 6A). Consistent with ZBP1 as an interferon-stimulated gene, SARS-CoV-2 infection caused the upregulation of *ZBP1* mRNA levels

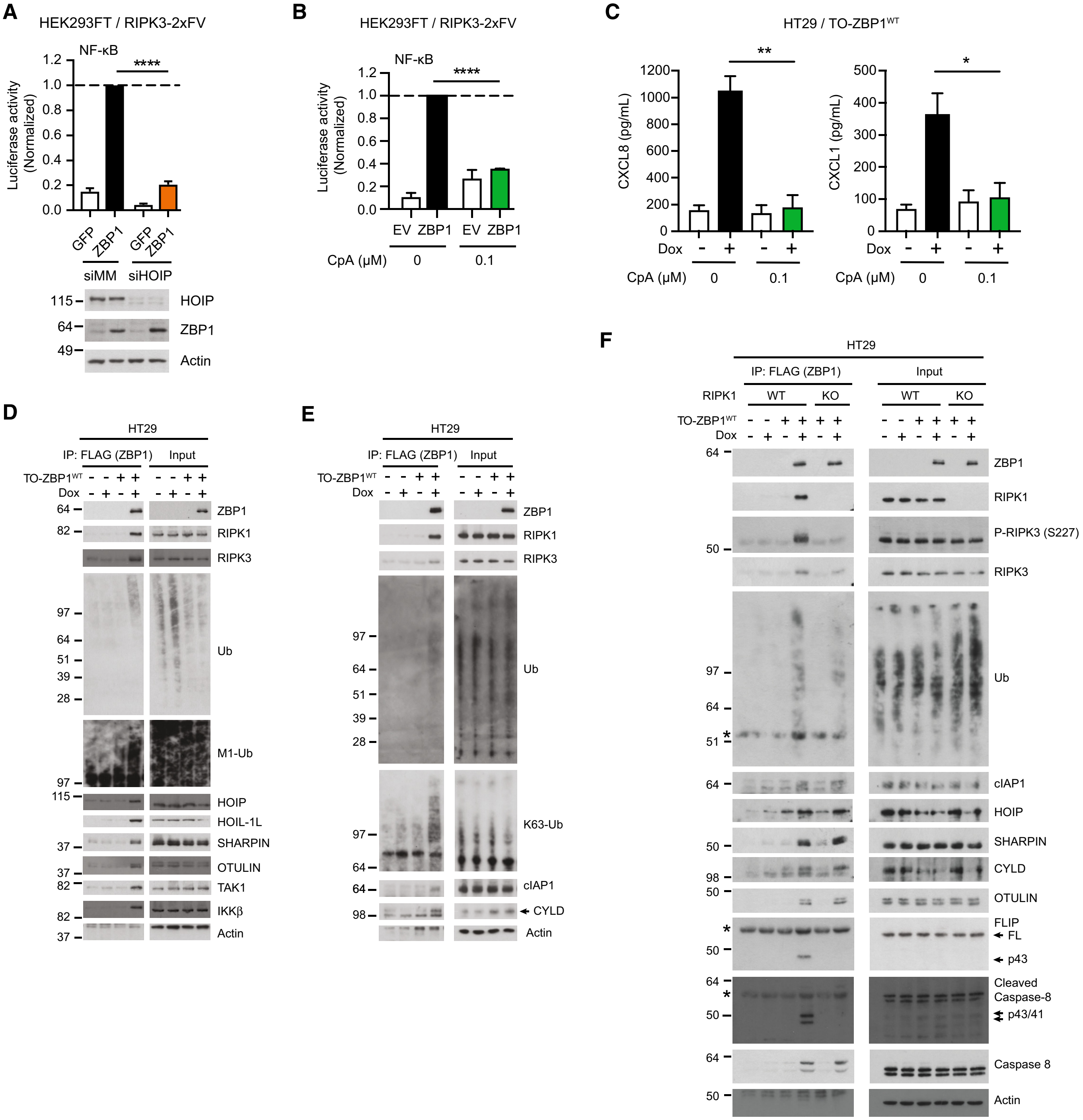


**Figure 4.**


© 2022 The Authors

**Figure 4. cIAP1 and LUBAC facilitate ZBP1-induced inflammatory signaling.**

A NF-κB activity in HEK293FT/RIPK3-2xFV cells transfected with siRNA targeting HOIP (siHOIP) or a mismatch sequence (siMM), and 48 h later transfected with dual luciferase reporters and ZBP1 or GFP (control). Luciferase reporter activities were measured 24 h after transfection and normalized to the siMM+ZBP1 samples. Data are plotted as mean with S.E.M. ($n = 4$ biological replicates). A Welch's $t$-test was used to test for the statistical difference between indicated conditions. ****$P < 0.0001$. HOIP knockdown and ZBP1 expression levels were analyzed by Western blotting. Blots are representative of three biological replicates.

B NF-κB activity in HEK293FT/RIPK3-2xFV cells treated with 0 or 100 nM CpA and transfected with dual luciferase reporters and ZBP1 or EV. Luciferase reporter activities were measured 24 h after transfection and normalized to the 0 nM CpA + ZBP1 sample. Data are plotted as mean with S.E.M. ($n = 3$ biological replicates). A Welch's $t$-test was used to test for the statistical difference between indicated conditions. ****$P < 0.0001$.

C Cytokine concentration in the culture media of HT29/TO-ZBP1$^{WT}$ cells treated with 0 or 100 nM CpA and 0 or 50 ng/ml Dox as indicated for 24 h. Data are plotted as mean with S.E.M. ($n = 3$ biological replicates). Unpaired $t$-tests were used to test for the statistical difference between indicated conditions. **$P = 0.0049$; *$P = 0.0394$.

D–F Western blot analysis of anti-FLAG (ZBP1) immunoprecipitation from HT29 or HT29/TO-ZBP1$^{WT}$ or HT29/RIPK1-KO clone aA3/TO-ZBP1$^{WT}$ cells treated with 0 or 500 ng/ml Dox for 16 h. Asterisk indicates antibody heavy chain signal in IP samples. Blots are representative of at least (D) two and (E–F) three biological replicates.

Source data are available online for this figure.

in Calu-3 cells between 48 and 72 h after infection in a dose-dependent manner (Fig 6B). This correlated when mRNA levels of *IL-6*, *TNF*, *CXCL10*, *CXCL8*, and *CXCL1* were upregulated and IL-6 and CXCL10 were secreted (Fig 6B and C). The timing of the inflammatory response also correlated with the increase in intracellular SARS-CoV-2 levels, as determined by an increase in viral transcripts and accumulation of Spike protein in the host cells (Fig EV5D and E).

To investigate the contribution of ZBP1 to SARS-CoV-2-induced cytokine production, we generated three independent Calu-3 cell lines with stable knockdown of *ZBP1* (shZBP1) and control cells expressing a nontargeting shRNA (shMM). IFNβ-induced ZBP1 expression showed that all three *ZBP1*-targeting shRNAs reduced ZBP1 levels relative to the shMM on protein expression levels, and that shZBP1-50 was the most effective (Fig 6A). Infection with SARS-CoV-2 (MOI = 2) showed that knockdown of *ZBP1* attenuated the expression of the measured cytokine genes, as well as the production of CXCL10 and to a lesser degree of IL-6 (Fig 6D and E). The attenuation of the pro-inflammatory response after infection correlated with the efficacy of the individual shRNA to silence *ZBP1* expression; shZBP1-50 resulted in a substantially stronger attenuation than did shZBP1-52 or shZBP1-53. Compared to changes in inflammatory cytokine levels, *ZBP1* knockdown only mildly attenuated the infection-induced expression of *IFNB* and the interferon-induced gene IFIT1 (Fig EV5F). The silencing of *ZBP1* did not affect the intracellular virus amount 3 days after infection as the virus level was similar in all cell lines (Fig EV5G). Together, these data suggest that ZBP1, subsequent to its transcriptional upregulation by SARS-CoV-2 infection, stimulates the production of inflammatory cytokines. It remains to be determined if ZBP1-dependent cell death contributes to the cytokine production induced by SARS-CoV-2 infection.

In summary, we show that ZBP1 promotes inflammatory signaling in HT29 cells through the recruitment of RIPK3 and RIPK1 and the formation of a proinflammatory complex containing ubiquitin ligases and deubiquitinases of the K63- and M1-linked polyubiquitin machinery, where ZBP1 and RIPK1 are ubiquitinated to promote MAPK- and NF-κB signaling. Our data further suggest that caspase-8 is recruited to restrain the kinase activity of RIPK3, which otherwise promotes inflammatory signaling and possibly necroptosis dependent on the kinase activity of RIPK1 and RIPK3 (Fig 6F). At higher expression levels, ZBP1 induces predominantly caspase-8-mediated apoptosis in HT29 cells.

## Discussion

### RIPK3 as a scaffolding kinase for inflammatory signaling

RIPK3 was initially reported to stimulate both inflammatory signaling and cell death, but RIPK3-deficient cells showed normal NF-κB signaling in response to the stimulation of TNFR1, B and T cell receptors, and Toll-like receptors (TLRs) 2 and 4, excluding the role of RIPK3-mediated inflammatory signaling in those contexts (Sun *et al*, 1999; Yu *et al*, 1999; Newton *et al*, 2004). Here, we demonstrate that RIPK3 is a *bona fide* inflammatory mediator in ZBP1-induced inflammatory signaling in human cells. Interestingly, the role of RIPK3 in ZBP1-induced inflammatory signaling depends on its RHIM domain and does not require its kinase activity, which is in contrast to its role in necroptosis where kinase activity is essential. This indicates that RIPK3 functions as a scaffold and not as a kinase in ZBP1-induced inflammatory signaling.

This is reminiscent of the scaffolding role of other receptor-associated kinases in mediating inflammatory signaling, including RIPK1 in TNFR1 signaling, RIPK2 in NOD2 signaling, and IL-1R-associated kinases (IRAKs) in IL-1R signaling (Ea *et al*, 2006; Koziczak-Holbro *et al*, 2008; Ordureau *et al*, 2008; Hrdinka *et al*, 2018). While these kinases serve as scaffolds for the formation of K63- and/or M1-Ub, ubiquitination of RIPK3 was not consistently detected in response to ZBP1 expression. Instead, ZBP1 and RIPK1 were modified with K63- and/or M1-Ub. Together with the requirement of RIPK1 and the RHIM of RIPK3 for ZBP1 inflammatory signaling, this suggests that RIPK3 may mediate signaling by RHIM-mediated recruitment and/or stabilization of RIPK1 in the ZBP1 complex, whereas RIPK1 and ZBP1 are the primary ubiquitination targets to facilitate NF-κB activation. Since the deletion of RIPK1 reduced the association of RIPK3, in particular its phosphorylated form, with ZBP1, RIPK1 may also contribute to the stabilization of a RHIM-mediated ZBP1-RIPK3-RIPK1 complex. This would be concordant with previous reports showing that a RIPK1-RIPK3 interaction precedes formation of RIPK3 oligomers during the activation of RIPK3 (Li *et al*, 2012; Wu *et al*, 2014). Our investigation of the ZBP1 signaling complex relied on Dox-induced overexpression of ZBP1, which precludes a detailed time-resolved study of the assembly of the signaling complex and of ubiquitination dynamics of complex components in response to ligand binding. Such investigations will be warranted when cognate ZBP1 ligands are better defined in order to gain detailed insights into the assembly of the ZBP1 signaling complex.

© 2022 The Authors

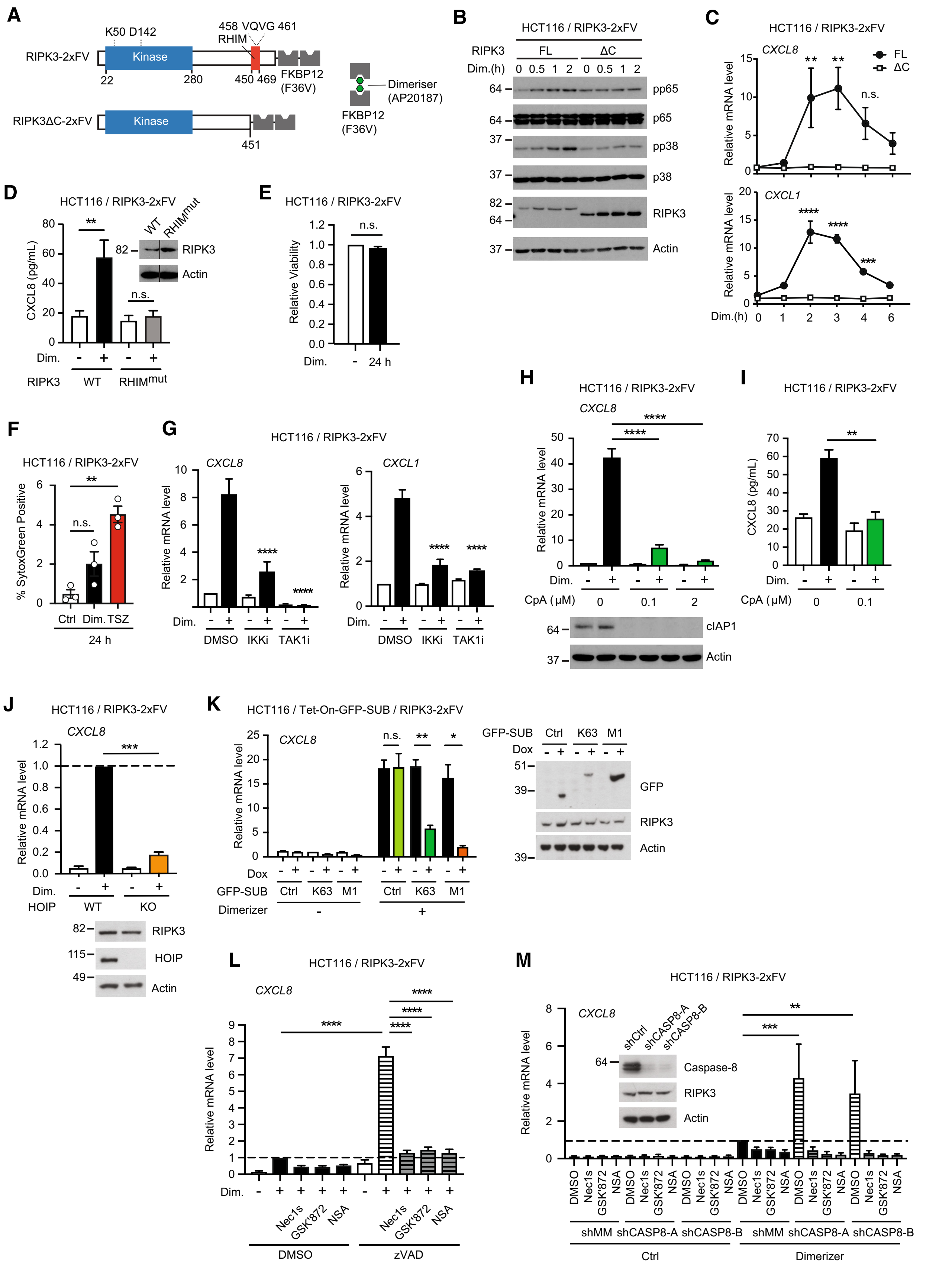

Figure 5.

 © 2022 The Authors

**Figure 5. RIPK3 oligomerization induces ubiquitination-mediated inflammatory signaling.**

A Schematic illustration of the chemical-inducible RIPK3 oligomerization system.

B Western blot analysis of HCT116/RIPK3-2xFV cells (FL and ΔC) treated with 100 nM dimerizer. Blots are representative of two biological replicates.

C Time courses of mRNA levels of *CXCL8* and *CXCL1* in HCT116/RIPK3-2xFV cells (FL and ΔC) in response to treatment with 100 nM dimerizer. Data are plotted as mean with S.E.M. ($n = 3$ biological replicates). Two-way ANOVA and Tukey's multiple comparisons test were used to test for statistical differences between 0 h and indicated time points in FL cells. n.s. = not significant ($P = 0.1360$); $**P = 0.0046$ for FL 2 h, $P = 0.0012$ for FL 3 h; $****P < 0.0001$; $***P = 0.0008$.

D CXCL8 concentration in the culture media of HCT116/RIPK3-2xFV cells (WT and RHIM$^{mut}$) treated with 100 nM dimerizer for 24 h. In RIPK3-RHIM$^{mut}$ three key residues, VQV, of the RHIM region was changed to AAAA. Data are plotted as mean with S.E.M. ($n = 5$ biological replicates). One-way ANOVA and Sidak's multiple comparisons tests were used to test for statistical differences between indicated time points. $**P = 0.0011$; n.s. = not significant ($P = 0.9301$). Inset: Western blot analysis to determine RIPK3 expression levels in one biological replicate. Line indicates that image was cut and spliced to remove nonrelevant lanes from the scanned blot.

E Relative viability of HCT116/RIPK3-2xFV cells treated with 0 or 100 nM dimerizer for 24 h. Data are presented as mean with S.E.M. ($n = 3$ biological replicates). A Welch's *t*-test was used to test for statistical differences between indicated conditions. n.s. = not significant ($P = 0.2294$).

F Cell death analysis of HCT116/RIPK3-2xFV cells treated with 0 or 100 nM dimerizer or TSZ (100 nM CpA, 20 μM zVAD and 1 ng/ml TNF) for 24 h using SytoxGreen to stain dead cells. Data are plotted as mean with S.E.M. ($n = 3$ biological replicates). One-way ANOVA and Sidak's multiple comparisons test were used to test for statistical difference between indicated conditions. n.s., not significant ($P = 0.1032$); $**P = 0.0014$.

G, H Relative mRNA levels of *CXCL8* and *CXCL1* in HCT116/RIPK3-2xFV cells pretreated with DMSO, TAK1 inhibitor, or IKK inhibitors for 1 h or 100 nM CpA for 0.5 h before treatment with 0 or 100 nM dimerizer for 3 h. Data are presented as mean with S.E.M. ($n = 3$ biological replicates). One-way ANOVA and Sidak's multiple comparisons tests were used to test for statistical differences between indicated conditions and DMSO+dimerizer–treated condition. $****P < 0.0001$. (H) Cell lysates were analyzed by Western blotting for cIAP1 levels. Blots are representative of two biological replicates.

I CXCL8 concentration in the culture media of HCT116/RIPK3-2xFV cells pretreated with 100 nM CpA for 1 h before treatment with 0 or 100 nM dimerizer for 24 h. Data are presented as mean with S.E.M. ($n = 3$ biological replicates). An unpaired *t*-test was used to test for the statistical difference between indicated conditions. $**P = 0.0042$.

J Relative CXCL8 mRNA levels in WT or HOIP-knockout HCT116/RIPK3-2xFV cells treated with 0 or 100 nM dimerizer for 3 h. Data are plotted as mean with S.E.M. ($n = 3$ biological replicates). A Welch's *t*-test was used to test for the statistical difference as indicated. $***P = 0.0010$. Cell lysates were loaded for Western blot analysis.

K Relative *CXCL8* mRNA levels in HCT116/Tet-On-GFP-K63-SUB/RIPK3-2xFV, HCT116/Tet-On-GFP-M1-SUB/RIPK3-2xFV and HCT116/Tet-On-GFP/RIPK3-2xFV cells treated with 0 or 100 ng/ml Dox for 48 h before stimulated with 0 or 100 nM dimerizer for 3 h. Data are presented as mean with S.E.M. ($n = 4$ biological replicates). Brown-Forsythe and Welch ANOVA tests and Dunnet's T3 multiple comparisons test were used to test for statistical differences between indicated conditions. n.s. = not significant ($P = 0.9997$); $**P = 0.0030$; $*P = 0.0366$. Cells were analyzed by Western blotting for the inducible-expression levels of GFP (control) and GFP-SUBs. Blots are representative of three biological replicates.

L Relative chemokine mRNA levels in HCT116/RIPK3-2xFV cells pretreated with 0 or 20 μM zVAD in combination with DMSO, 10 μM Nec1s, 10 μM GSK′872, or 1 μM NSA for 1 h, followed by treatment with 0 or 100 nM dimerizer for 3 h. Data are plotted as mean with S.E.M. ($n = 3$ biological replicates). $****P < 0.0001$. One-way ANOVA and Sidak's multiple comparison test were used to test for statistical differences between indicated conditions.

M Relative *CXCL8* mRNA levels in HCT116/RIPK3-2xFV cells stably knocked down against mismatch (shMM) or two different sites of caspase-8 (shCASP8-A and shCASP8-B), pretreated with DMSO, 10 μM Nec1s, 10 μM GSK′872, or 1 μM NSA for 1 h followed by treatment with 0 or 100 nM dimerizer for 3 h. Data are plotted as mean with S.E.M. ($n = 3$ biological replicates). Two-way ANOVA and Tukey's multiple comparison tests were used to test for statistical differences between indicated conditions. $***P = 0.0001$; $**P = 0.0035$. Cell lysates from one biological replicate were analyzed by Western blotting to determine caspase-8 levels.

Source data are available online for this figure.

It was intriguing that RIPK3, in addition to its scaffolding role, also promoted inflammatory signaling by ZBP1 in a kinase activity-dependent manner when caspase-8 activity was inhibited. This is concordant with the reported inflammatory signaling pathway mediated by RIPK3 and RIPK1 kinase activity and by MLKL in response to TSZ treatment (Zhu *et al*, 2018). This suggests that caspase-8 represents a checkpoint switch for RIPK3 kinase activity-mediated signaling also in the context of ZBP1 by suppressing the kinase activity-dependent inflammatory signaling pathway after the engagement of RIPK3. It remains to be defined how RIPK3 kinase activity- and MLKL-dependent signaling leads to inflammatory gene activation, and if this is associated with necroptotic cell death. Nonetheless, our observations suggest that the default role of RIPK3 in ZBP1 inflammatory signaling in human cells is as a scaffolding kinase and that the kinase activity-dependent pathway is activated when caspase-8 activity is antagonized, such as during infection by viruses encoding caspase inhibitors.

## ZBP1-mediated inflammatory signaling and cell death responses

Since the discovery that ZBP1, via RIPK3, induces necroptosis during murine cytomegalovirus (MCMV) infection, its role in cell death during infection and embryonic development has been well established (Upton *et al*, 2012; Lin *et al*, 2016; Newton *et al*, 2016; Thapa *et al*, 2016; Kuriakose & Kanneganti, 2018; Jiao *et al*, 2020; Wang *et al*, 2020). Our study expands the understanding of ZBP1's function as we uncover that human ZBP1, in a ligand binding-dependent manner, triggered RIPK3-mediated inflammatory signaling at a lower expression threshold and at earlier time points than needed for stimulation of cell death. A key question arising from this observation and previous studies on RIPK3-mediated cytokine production (Yatim *et al*, 2015; Najjar *et al*, 2016; Zhu *et al*, 2018) was whether the inflammatory signaling and cytokine production are dependent on cell death or is an independent process. By measuring ZBP1-induced cell death and cytokine levels in parallel and in conjunction with chemical inhibitors of caspase-8 and RIPK1/3, we demonstrate that ZBP1 stimulates the production of cytokines independently of cell death in HT29 cells. This does not exclude that cell death signaling contributes to ZBP1-induced inflammatory responses in other contexts.

It is tempting to speculate that ZBP1, akin to other innate immune receptors, induces ubiquitin-dependent NF-κB signaling as a first line of defense to recruit innate immune cells (e.g. neutrophils and monocytes), and that activation of programmed cell death is a

© 2022 The Authors

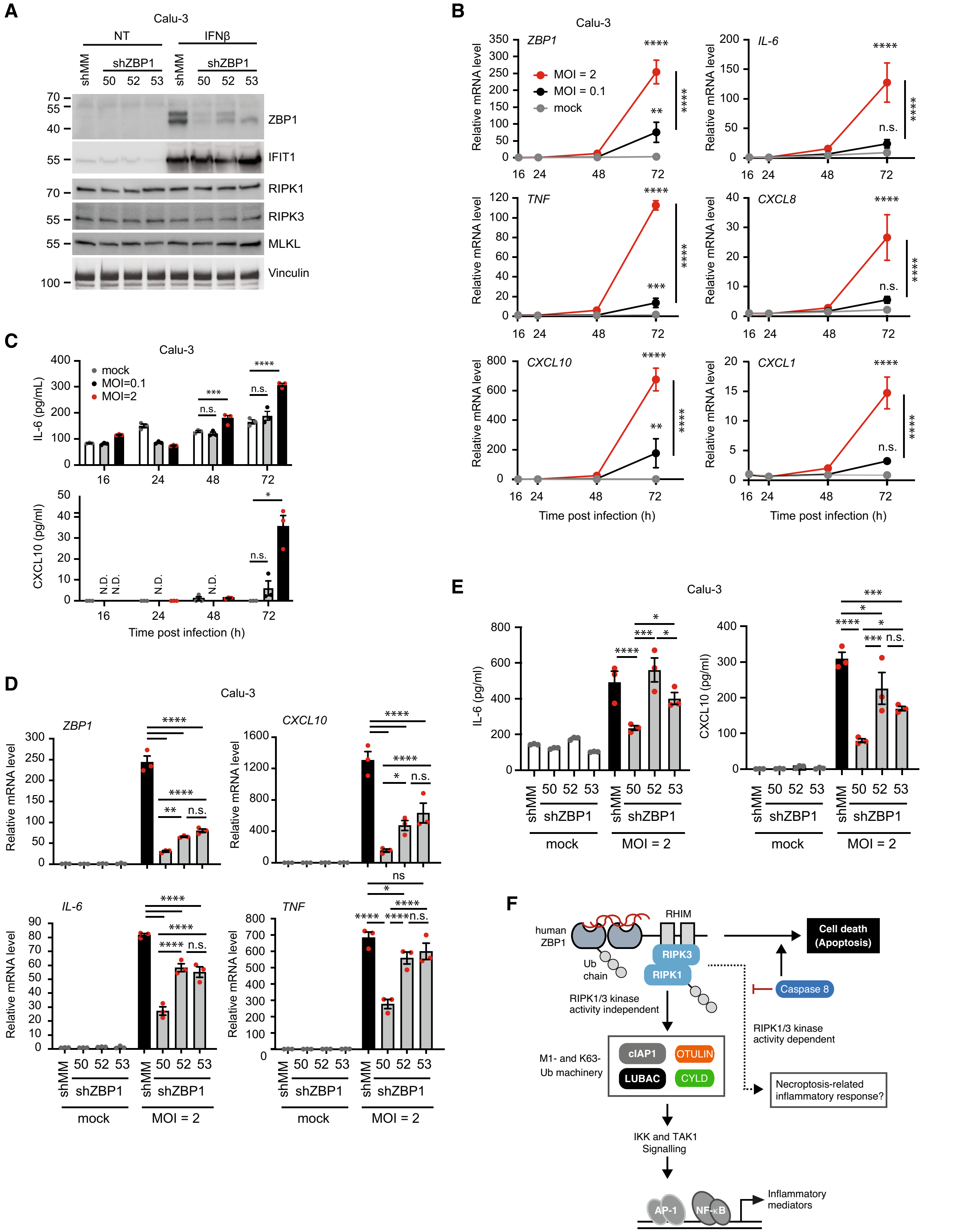


Figure 3.


© 2022 The Authors

**Figure 6. ZBP1 mediates SARS-CoV-2-induced cytokine production.**

A Western blot analysis of ZBP1, RIPK1, RIPK3, and MLKL levels in Calu-3 cell lines with stable expression of shRNAs targeting ZBP1 or a mismatch sequence (shMM), untreated (NT) or treated with 10 ng/ml IFNβ for 48 h. Blots are representative of two biological replicates.

B, C Relative mRNA levels (B) or cytokine levels in the culture media (C) of indicated genes in Calu-3 cells infected with mock or SARS-CoV-2 viruses at indicated MOIs for 16, 24, 48 or 72 h. Data are plotted as mean with S.E.M. ($n = 3$ biological replicates). Two-way ANOVA and Tukey's (B) or Dunnet's (C) multiple comparison test were used to test for statistical differences between each infected sample and mock-treated sample and between the two different MOIs at 72 h time point. For (B): ****$P < 0.0001$; ***$P = 0.0006$, **$P = 0.0022$ for *ZBP1*, $P = 0.0072$ for *CXCL10*; n.s. = not significant ($P > 0.05$). For (C): ****$P < 0.0001$; ***$P = 0.0002$; *$P = 0.0244$; n.s. = not significant ($P > 0.05$).

D, E Relative mRNA levels (D) or secreted cytokine levels (E) of indicated genes in Calu-3 cells knocked down against mismatch sequence (shMM) and ZBP1 at three different sites (shZBP1-50, shZBP1-52 and shZBP1-53) infected with mock or SARS-CoV-2 virus at MOI = 2 for 72 h. Data are presented as mean with S.E.M. ($n = 3$ biological replicates). One-way ANOVA and Sidak's multiple comparisons test were used to test for statistical differences between indicated conditions. n.s. = not significant, *$P < 0.05$; **$P < 0.01$; ***$P < 0.001$; ****$P < 0.0001$.

F Schematic model of the proposed ZBP1-induced signaling pathways for inflammatory responses and cell death in human cells.

Source data are available online for this figure.

mechanism invoked during pathological conditions where ZBP1 expression is highly induced by interferons. Intriguingly, the major mode of cell death in ZBP1-expressing HT29 cells appeared to be RIPK1- and caspase-mediated apoptosis, whereas treatment of the cells with TSZ induced necroptosis.

Intriguingly, RIPK1 was essential not only for inflammatory signaling but also for ZBP1-induced cell death in HT29 cells, which is contrary to previous *in vivo* studies in murine systems where RIPK1 restricts ZBP1-RIPK3-induced necroptosis during development and in skin inflammation (Lin *et al*, 2016; Newton *et al*, 2016; Devos *et al*, 2020). Whether this represents a difference between mice and human or is specific to the experimental systems is interesting and should be addressed in future studies. Irrespective, our study indicates that ZBP1-induced inflammatory signaling may contribute to pathological inflammatory conditions where ZBP1-mediated cell death has been reported (Upton *et al*, 2012; Kuriakose *et al*, 2016; Lin *et al*, 2016; Newton *et al*, 2016; Thapa *et al*, 2016; Jiao *et al*, 2020).

In line with this, ZBP1 was recently found to contribute to TLR3/4-induced inflammatory signaling in mice by mediating RIPK1 recruitment to TRIF (Muendlein *et al*, 2022). Moreover, several studies published during the revision of this manuscript show that ZBP1 mediates fatal autoinflammation and immune pathology in mice caused by mutations in the RNA deaminase ADAR1 (de Reuver *et al*, 2022; Hubbard *et al*, 2022; Jiao *et al*, 2022). While ZBP1 was found to contribute to cell death, inhibition of caspase-8-mediated apoptosis and RIPK3-MLKL-mediated necroptosis did not rescue the pathology and death of the animals caused by ADAR1 mutation. Instead, ZBP1 was proposed to mediate pathogenic type-I IFN responses *in vivo*. Moreover, oligomerization of ectopic ZBP1 in murine cells was found to stimulate the expression of pro-inflammatory cytokines in a manner that was dependent on RIPK1 and suppressed by caspase-8 (Hubbard *et al*, 2022). Our study complements these reports by showing a proinflammatory signaling complex associated with ZBP1. Together with previous studies of ZBP1, this points to ZBP1 as a multifunctional pattern recognition receptor that, depending on the context, can induce cell death or promote inflammatory signaling and/or IFN responses.

ZBP1 is upregulated by type-I IFNs as part of the antiviral host defense. In line with this, RNAseq datasets from COVID-19 patients show that ZBP1 is upregulated by SARS-CoV-2 infection and that its expression correlates with the expression of pro-inflammatory chemokines and cytokines. Functionally, we find that ZBP1, subsequent to being upregulated by SARS-CoV-2 infection, contributes to the late-onset production of cytokines and chemokines in Calu-3 cells. Although the involvement of ZBP1-induced cell death in SARS-CoV-2-induced inflammation remains to be investigated, our data suggest a role for ZBP1 in the host response to SARS-CoV-2.

## Materials and Methods

### Cell lines

Cell lines used in this study: HT29 human colorectal adenocarcinoma (ATCC #HTB-38), HCT116 human colorectal carcinoma (ATCC #CCL-247), U2OS human osteocarcoma (ATCC #HTB-96), HEK293T human embryonal kidney transformed with SV40 large T antigen (ATCC #CRL-3216), HEK293FT human embryonal kidney transformed with SV40 large T antigen (Thermo Fisher Scientific #R70007), HL60 human acute promyelocytic leukemia (ATCC #CCL-240), THP1 human acute monocytic leukemia (ATCC #TIB-202), Calu-3 human lung adenocarcinoma (ATCC #HTB-55), Phoenix-Ampho human kidney epithelial (ATCC #CRL-3213). HT29, HCT116, U2OS, Calu-3, HEK293FT, THP1 cells were authenticated by STR profile analysis (Eurofins Genomics).

All cell lines were cultured at 37°C and 5% $CO_2$ in growth medium supplemented with 10% v/v fetal bovine serum (FBS, Labtech FCS-SA), 60 µg/ml penicillin and 100 µg/ml streptomycin (PS, Thermo Fisher 15070). The growth medium for HT29 and HCT116 cells was McCoy's 5A (Modified; Thermo Fisher 26600), for U2OS/NOD2 (Fiil *et al*, 2013), HEK293FT and Phoenix-Ampho was DMEM (Thermo Fisher 31966–021), for THP1 cells was RPMI (Thermo Fisher 42401042) supplemented with GlutaMAX (Thermo Fisher 35050061), Sodium Pyruvate (Gibco 11360-039) and 50 µM 2-mercaptoethanol (Gibco 31350-010), for HL60 cells was RPMI supplemented with GlutaMAX, and for Calu-3 cells was MEM (Thermo Fisher 11090081) supplemented with GlutaMAX, Sodium Pyruvate and nonessential amino acids (Thermo Fisher 11140035). Differentiated HL60 cells were obtained by culturing in complete growth media supplemented with 1.3 %DMSO (v/v; Sigma D2650) for 7 days in culture. Cells were routinely checked for *Mycoplasma* Spp. contamination with the MycoAlert Mycoplasma Detection kit (Lonza LT-07). HT29, HCT116, U2OS, Calu-3, HEK293FT, THP1 cells were authenticated by STR profile analysis (Eurofins Genomics).

© 2022 The Authors

### Isolation of primary neutrophils

Primary human neutrophils were obtained from healthy donors with their written informed consent by The Oxford Radcliffe Biobank with project number ORB 20/A136. The study is authorized by South Central—Oxford C Research Ethics Committee (Ref# 19/SC/0173). Neutrophils were isolated from 50-ml Ficoll-layered blood cone using EasySep Human Neutrophil Isolation Kit (StemCell 17957) following manufacturer's instructions.

### Generation of HT29 cells with Dox-inducible expression of ZBP1

For lentivirus production, HEK293T cells were transfected with C-terminally FLAG-tagged wild-type or Zα1α2-mutant human ZBP1-expressing transducing vectors in the doxycycline-inducible Tet-On pDG2 backbone (De Groote *et al*, 2016) together with the pCMV delta R8.91 gag-pol–expressing packaging plasmids and pMD2.G VSV-G-expressing envelope plasmid. HT29 and HT29/RIPK1-KO clones were transduced using 100 µl 0.45 µm syringe-filtered lentivirus-containing supernatant in 12-well plate. 72 h after transduction, cells were selected with 1 µg/ml puromycin (Invitrogen ant-pr).

To determine the percentage of cells expressing FLAG-ZBP1, $2.5 \times 10^4$ cells/well were plated in a 24-well plate and treated with 50 or 500 ng/ml Dox for 24 h. Cells were trypsinized and fixed with 4% PFA for 20 min, washed with PBS, then permeabilized and blocked using 0.1% saponin with 3% BSA in PBS for 1 h. Fixed cells were then incubated with anti-FLAG antibody (rabbit, Cell Signaling 14793) for 1 h at RT, washed three times with blocking buffer, then incubated with anti-Rabbit-488 (donkey, Thermo Fisher A-21206) for 1 h at RT. Cells were then pelleted by centrifugation, washed three times with PBS, and analyzed on a BD LSRFortessa cell analyzer.

### Production of lentiviral particles

For the production of lentiviral particles, HEK293FT cells were plated at a density of $3.5 \times 10^6$ cells in 10 cm dishes in 15 ml complete growth media. The next day, they were transfected with a mixture of 1.5 ml OptiMEM (Gibco 31985), 36 µl FuGENE HD (Promega E2311), 6 µg psPAX2 vector, 1.5 µg pMD.G (VSVG) and 4.5 µg lentiviral vector. A 24 h after transfection, transfection reagent-containing media were replaced with 10 ml complete growth media to allow the secretion of lentiviral particles for 72 h. Virus-containing supernatant was filtered, and lentiviral particles were precipitated as described for retroviral particles or directly frozen at −80°C for preservation.

### Generation of cell lines stably expressing RIPK3 variants

To generate RIPK3-expressing HT29, HCT116, and HEK293FT cell lines, LZRS-zeo-based retroviral vectors were used (Rodriguez *et al*, 2016). To produce retroviral particles, Phoenix-Ampho cells were plated at a density of $3.5 \times 10^6$ cells in 10 cm dishes in 15 ml complete growth media. The next day, transfection was carried out with a mixture of 1.5 ml OptiMEM, 36 µl FuGENE HD, 1.2 µg pMD.G plasmid and 10.8 µg of the retroviral vector, and media was replaced 24 h after transfection. Two batches of retroviral particles were collected, respectively at 48 and 72 h posttransfection. Retroviral particles were precipitated by incubating 0.45 µm syringe-filtered culture media in 150 mM NaCl and 5% PEG-8000 at 4°C overnight, followed by centrifugation at 3,500 *g* for 15 min. Pellets were resuspended in 150 µl sterile PBS and stored at −80°C until use.

For retroviral transduction, between 1 and $3 \times 10^5$ cells were seeded into 6-well plates. The next day, cells were transduced using 1 ml virus-containing supernatant or 25 µl precipitated viral particles in the presence of 10 µg/ml polybrene in a total of 2 ml complete growth medium. Cells were incubated with virus-containing supernatant for 24 h before replaced with complete growth medium to rest. 48–72 h after transduction, cells were selected with 250 ng/µl zeocin (Invitrogen R250) until two passages after the complete elimination of nontransduced cells in the control well. After selection, HEK293FT/RIPK3-2xFV cells were subjected to FACS for sorting of a low-mCherry RIPK3-expressing HEK293FT/RIPK3-2xFV population.

To generate U2OS/NOD2 cells with stable RIPK3 expression, RIPK3, RIPK3-2xFV, and RIPK3ΔC-2xFV were subcloned into pBABE-puro plasmids using primers 5′-ACGCGTATGTCGTGCGTC AAGTTATG-3′ and 5′-GTCGACTTACTTATCGTCGTCATCCTTGTAA TCTTTCCCGCTATGATTATACCAAC-3′ (matching the C-terminus of RIPK3, adding FLAG tag sequence) or 5′-GTCGACTTACTTATCG TCGTCATCCTTGTAATCTTCCAGTTTTAGAAGCTCCAC-3′ (matching the C-terminus of FV, adding FLAG tag sequence) to amplify the corresponding sequences from LZRS-zeo plasmids. U2OS/NOD2 cells were transduced with retroviral particles generated in Phoenix-Ampho cells transfected with the pBabe-puro plasmids and were selected with 1 µg/ml puromycin.

### Generation of HCT116 cells with inducible GFP-SUB expression

To generate HCT116/Tet-On-GFP-SUB/RIPK3-2xFV cells, HCT116 cells were first transduced with lentiviral particles generated from pLenti-CMV-Blast plasmids carrying the Tet Repressor gene. During selection with 5 µg/ml blasticidin (Thermo Fisher A1113903), single clones were isolated, and clone C4 with high levels of Tet-Repressor expression was used for the next steps.

HCT116/Tet-On clone C4 was plated at a density of $5 \times 10^4$ cells per well in 12-well plates, and transduced with 3 µl precipitated lentiviral particles generated with pLVX-tight-puro plasmids encoding GFP, GFP-K63-SUB, or GFP-M1-SUB sequences (Hrdinka *et al*, 2016). After selection with 1 µg/ml puromycin (Invitrogen ant-pr) in the presence of 5 µg/ml blasticidin, HCT116/Tet-On-GFP, HCT116/Tet-On-GFP-K63-SUB and HCT116/Tet-On-GFP-M1-SUB cells were transduced with retroviral particles produced from LZRS-zeo-RIPK3-2xFV plasmids and selected with the combination of 5 µg/ml blasticidin, 1 µg/ml puromycin, and 250 ng/µl zeocin.

### Construction of RIPK1 knockout cell lines by CRISPR/Cas9

Three different RIPK1 CRISPR/Cas9 knockout plasmids (Santa Cruz sc-400377) encoding the guide RNA targeting sequences GG CTTTGCGTTGACGTCATTC (gRNAa), GCTCGGGCGCCATGTAGT AG (gRNAb) and CGGCTTTCAGCACGTGCATC (gRNAc) were transfected separately into HT29 cells using a mixture of 500 µl OptiMEM, 20 µl Lipofectamine LTX (Invitrogen 15338100),

 © 2022 The Authors

3.75 μg of each plasmid, and 3.75 μl PLUS reagent (Invitrogen 15338100) following the manufacturer's instructions. Media was replaced at 24 h post-transfection and cells were sorted at 36 h post-transfection using FACS for the top 10% GFP-positive cells. After sorting, cells were seeded to obtain single clones in complete growth media containing 50 μg/ml gentamicin. Knockout clones were validated by Western blotting and genotyping. HT29/RIPK1-KO clone aA3 was generated using gRNAa, HT29/RIPK1-KO clone bC5 with gRNAb, HT29/RIPK1-KO clone cA5 with gRNAc.

### Generation of shRNA-mediated stable knockdown cell lines using lentiviral particles

Lentiviral particles were used to construct stable cell lines knocked down against mismatch control sequence (shMM, Sigma SHC002) or target genes. shRNA plasmids used for this study are pLKO.1-based targeting the following sequences: shCASP8-A (Sigma SHCLND, TRCN0000377309): CACCAGGCAGGGCTCAAATTT; shCASP8-B (Sigma SHCLND, TRCN0000376481): GGAGCTGCTCT TCCGAATTAA; shZBP1-50 (Sigma SHCLNG TRCN0000123050): GCACAATCCAATCAACATGAT; shZBP1-52 (Sigma SHCLNG TRCN 0000123052): CCACATGAAATCGTGCTTTCT; shZBP1-53 (Sigma SHCLNG TRCN0000123053): CCAAGTCCTCTACCGAATGAA.

For the transduction of lentiviral particles, Calu-3 cells were seeded in 10-cm dishes at a density of 7.5 × $10^5$ cells per dish, and transduced by incubation with 750 μl virus-containing supernatant in 10 ml complete growth medium containing 4 μg/ml polybrene for 24 h. 72 h after transduction, cells were selected with 1 μg/ml puromycin.

### siRNA-mediated gene knockdown

Knockdown of RIPK3 was achieved using Accell siRNA SMARTpool (Horizon Discovery E-003534-00-0005, Gene ID 11035), with Accell siRNA nontargeting pool (Horizon Discovery D-001910-10-05) as control targeting mismatch sequence (siMM). Cells were plated at 40% confluency. The next day, siRNA was transfected using Dharmafect transfection reagent (Horizon Discovery T-2001-03) in a mixture of 200 μl OptiMEM, 3 μl Dharmafect and 0.03 nmol siRNA per 200,000 cells. Twenty-four hours after transfection, cells were split into desired number and format of wells in complete growth medium for treatment.

Knockdown of HOIP was achieved using custom-synthesized siRNA from Sigma (sense strand: GGCGUGGUGUCAAGUUUAA[dT][dT]; antisense strand: UUAAACUUGACACCACGCC[dT][dT]; Haas *et al*, 2009). siRNA against mismatch sequence (Sigma SIC001) was used as nontargeting control. Cells were plated in 12-well plates at 0.8–1.6 × $10^5$ per well, and transfected with 0.04 nmol siRNA in a mixture with 100 μl OptiMEM and 1 μl Lipofectamine RNAiMAX reagent (Invitrogen 100014472). Twenty-four hours after siRNA transfection, cells were trypsinized and reseeded for indicated applications.

### SARS-CoV-2 infection of Calu-3 cells

The Wuhan-like early European SARS-CoV-2 B.1, Freiburg isolate (FR4286, kindly provided by Professor Georg Kochs, University of Freiburg; Hoffmann *et al*, 2020), was propagated in Vero cells expressing human TMPRSS2 (Olagnier *et al*, 2020) and virus titer determined by $TCID_{50\%}$ as previously described (Fougeroux *et al*, 2021). Stocks were validated by sequencing before use in experiments.

Calu-3 epithelial lung cancer cells were cultured in DMEM (Lonza) supplemented with 10% heat-inactivated fetal calf serum, 200 IU/ml penicillin, 100 μg/ml streptomycin and 600 μg/ml L-glutamine prior to infection experiments. Calu-3 cells were seeded in flat-bottom 12-well plates (2 × $10^5$ cells/well in 1 ml media) or flat-bottom 6-well plates (5 × $10^5$ cells/well in 2 ml media). Upon reaching confluency of 50–70% 24–48 h after seeding, the cells were infected with SARS-CoV2 B.1 at MOIs of 0.5 or 2.0. The plates were incubated at 37°C and tilted every 15 min for 1 h to allow for virus adsorption. After 1 h, the media was replaced with fresh media. Supernatant and cell lysates were harvested after 16, 24, 48, and 72 h, respectively.

To inactivate any live virus prior to biochemical analyses, cell-free supernatant was incubated in 0.5% Triton-X (Sigma Aldrich 11332481001) for 30 min at room temperature. Cells for qPCR were lyzed in RNeasy lysis buffer (Qiagen 79254) containing 1% β-Mercaptoethanol and incubated for 10 min at room temperature. Cells for Western blotting were lyzed in RIPA buffer (Thermo Fisher 89901), supplemented with 4× XT sample buffer (BioRad 161–0791) and XT Reducing Agent (BioRad 161–0792), and heated at 95°C for 5 min. Virus-inactivated samples were used for subsequent analysis by ELISA, qPCR and Western blotting.

### Cell viability assay

The CellTitre-Glo® 2.0 reagent (Promega G9242) was used to determine cell viability according to the manufacturer's protocol. Cells were seeded in opaque 96-well plates in 100 μl complete growth media at a density of 3.3 × $10^3$ cells/well for 72-h time courses or 1.3 × $10^4$ cells/well for 24-h treatment. The next day, cells were stimulated with the appropriate chemicals (doxycycline, Sigma, D3072) diluted in 5 μl OptiMEM. Relative viability is calculated by dividing the average of measurement luminescence values of technical replicates for each treatment condition by one reference condition as specified for each figure.

### SytoxGreen exclusion assays

Cell death measurements was determined by SytoxGreen (Thermo Fisher 10768273) uptake using flow cytometry. HT29 cells were seeded in 24-well plates at a density of 2.5 × $10^4$ cells/well. 48 h post seeding, the cells were stimulated with the appropriate chemicals (doxycycline, Sigma D3072; zVAD, Fischer Scientific 17215270; Nec1s, Fisher Scientific 17215270; GSK′840, Fisher Scientific 16446647; GSK′872, Fisher Scientific 17261010; NSA, R&D 5025/10; TNF, PeproTech 300-01A-100uG; LCL161, Fisher Scientific 16426598). Following incubation, cells were stained with 1 μM SytoxGreen for 30 min. Media and subsequent PBS washes were collected and adherent cells were detached using TrypLE express (Fisher Scientific 11558856). Cells and debris were pelleted by centrifugation at 500 *g* for 5 min. The supernatant was collected and later used for ELISA assays. The cell pellet was then washed twice and resuspended in ice cold FACS buffer (PBS + 3% FBS).

© 2022 The Authors

SytoxGreen positive cells were analyzed using a BD LSRFortessa flow cytometer.

SytoxGreen-positive percentage of HCT116/RIPK3-2xFV cells were determined by SytoxGreen/Hoechst 33342 double-staining and confocal microscopy. Cells were seeded at 2000 per well in 96-well plates, treated with vehicle, 100 nM AP20187 (Clontech 635059) or combination of 100 nM CpA (SMAC mimetic compound, kindly provided by Tetralogic Pharmaceuticals), 20 μM zVAD and 1 ng/ml TNF in technical triplicates for 24 h before staining with 6 μM SytoxGreen (Thermo Fisher S7020) and 150 μM Hoechst 33342 (Thermo Fisher 62249) for 10 min under room temperature. Cells were imaged with Confocal 710MP. The number of SytoxGreen-positive cells and Hoechst 33342-positive cells were counted automatically on ImageJ setting Otsu threshold (1,600, max) and diameter between 20 and 200 μm. The average of technical triplicates was reported as one biological replicate.

### Enzyme-linked immunosorbent assay (ELISA)

For ELISA, cells were plated at a density of $1 \times 10^5$ cells/well in 24-well plates or $2 \times 10^5$ cells/well in 12-well plates, and stimulated the next day for 24 h with the appropriate chemicals (doxycycline, Sigma D3072; dimerizer, Clontech 635059; (5Z)-7-Oxozeaenol/TAK1i, Tocris 3604; IKK inhibitor VII, Merck Millipore 401486; IKK inhibitor XII, Merck Millipore 401491; zVAD, Santa Cruz sc-311560; Nec1s, Enzo BV-2535-1; GSK′872, Merck Millipore 5.30389.001; NSA, R&D 5025/10). Cell culture supernatants were centrifuged at 300 *g* for 5 min to remove debris, diluted as appropriate, and loaded in technical duplicates or triplicates. Measurement of cytokine concentrations was carried out with R&D DuoSet ELISA kits (CXCL8, DY208-05, CXCL1, DY275-05, CXCL10, DY266-05, IL-6, DY206-05) according to manufacturer's instructions. For data analysis, the absorbance at 540 nm of each well was subtracted from the 450 nm value. Generation of standard curves and interpolation of data were performed in GraphPad Prism. Where indicated, cells were collected after stimulation for Western blotting to assess the protein expression levels.

### Chemokine array

The chemokine array was performed following the manufacturer's protocol of Proteome Profiler Human Chemokine Array Kit (R&D ARY017). Pixel intensity from each chemokine dot area were quantified using Fiji. Of each detectable chemokine, fold change of Dox-treated condition over DMSO-treated condition was divided by the fold change of pixel intensities of positive control dots for normalization.

### Cytokine array

The cytokine array was performed following the manufacturer's protocol of Proteome Profiler Human XL Cytokine Array Kit (R&D ARY022B). Individual mean pixel intensity of each dot for each exposure time was quantified using Python. Raw pixel intensities were inverted (subtraction from 255 for 8-bit image) and background normalized by subtracting the mean intensity of each blot for fold-change analysis. The fold change of Dox-treated condition over DMSO-treated condition was normalized by dividing by the fold change of pixel intensities of positive control dots. The normalized fold change at different exposure times were plotted as a heatmap.

### RNA isolation, cDNA synthesis, and qPCR

RNeasy Mini kit (Qiagen 79254) was used for RNA isolation according to the manufacturer's instructions, and on-column DNA digestion was performed with RNase-free DNase Set (Qiagen 74106). For cDNA synthesis, around 1 μg RNA (10 μl) was incubated with 1 μl 10 μM random pentadecamers (IDT 169190224) and 0.5 μl 100 μM anchored oligo$(dT)_{20}$ primer (Sigma, custom synthesized) at 65°C for 5 min, before supplemented with 0.5 μl RevertAid reverse transcriptase (Thermo Scientific EP0441) and 0.5 μl RiboLock RNase inhibitor (Thermo Scientific EO0381) in a total volume of 20 μl RevertAid Reverse Transcriptase buffer (Thermo Scientific LT-02241). Samples were then subject to reverse transcription program of 10 min at 25°C, followed by 60 min at 42°C and 10 min at 70°C. The resulting cDNA was diluted as appropriate before quantitative PCR.

Quantitative PCR from the cDNA was performed using the SYBR Select qPCR mastermix (Applied Biosystems 4472908) using 2 μl cDNA, 1 μM forward primer and 1 μM reverse primer per reaction in a 10 μl reaction volume in technical duplicates. Primer pairs used were as following: Hypoxanthine phosphoribosyltransferase (*HPRT*; used as reference for normalization), 5′-AGCCAGACTTTGTTGGA TTTG-3′ and 5′-TTTACTGGCGATGTCAATAGG-3′; *CXCL1*, 5′-TCCT GCATCCCCCATAGTTA-3′ and 5′-CTTCAGGAACAGCCACCAGT-3′; *CXCL8*, 5′-TCTGGCAACCCTAGTCTGCT-3′ and 5′-AAACCAAGGC ACAGTGGAAC-3′; *CXCL10*, 5′-GTGGATGTTCTGACCCTGCT-3′ and 5′-GAGGATGGCAGTGGAAGTCC-3′; *ZBP1*, 5′-GAAGCAAGAATTCCC AGTCCAG-3′ and 5′-TCGAGAAAGCACGATTTCATGT-3′. *TNF*, 5′-TG CTGCAGGACTTGAGAAGA-3′ and 5′-GAGGAAGGCCTAAGGTCCAC-3′; *IL6*, 5′-TGCTGCAGGACTTGAGAAGA-3′ and 5′-GAGGAAGGCCTAA GGTCCAC-3′; *IFIT1*, 5′-GCGCTGGGTATGCGATCTC-3′ and 5′-CAGC CTGCCTTAGGGGAAG-3′; *IFNB1*, 5′-ATGACCAACAAGTGTCTCCT CC-3′ and 5′-GGAATCCAAGCAAGTTGTAGCTC-3′.

Interpolation of CT values was performed with the built-in software of the qPCR machine (Roche LightCycler 480 or Applied Biosystems 7500). Relative mRNA expression levels were calculated with the comparative CT method (Schmittgen & Livak, 2008).

### One-step qPCR

One-step RNA-to-Ct qPCR was used to determine the amount of intracellular SARS-CoV-2 following manufacturer's protocol (Thermo Fisher 4392938). A 100 ng RNA was loaded into each reaction with primers and probes for SARS-CoV-2 (IDT 10006713) or β-Actin (Thermo Fisher 4331182, Assay ID Hs00357333_g1) at manufacturer's recommended concentrations.

### Site-directed mutagenesis

RIPK3 RHIM-mutant was generated using a Q5 site-directed mutagenesis kit (NEB) on the LZRS-zeo-RIPK3-2xFV plasmid according to the manufacturer's protocol using primers. 5′-GC TGCAGGAGACAACAACTACTTG-3′ and 5′-TGCCGCCCCAGAGCAG TTGTATATG-3′.


© 2022 The Authors

### NF-κB dual-luciferase reporter assay

NF-κB dual luciferase assays were performed with the Dual Luciferase Reporter Assay System kit (Promega E1960). For ZBP1 expression NF-κB reporter assays, cells were plated in 24-well plates at a density of $4 \times 10^5$ cells/well. The next day, they were transfected using per well 125 ng pBIIX-Luc (NF-κB reporter plasmid; Saksela & Baltimore, 1993), 25 ng SV40-Renilla luciferase plasmid, 0.5 ng ZBP1-expressing pLenti6.3 plasmid (with pBabe-puro or pLenti6.3-GFP as control), and, where indicated, 100 ng pBabe-CYLD plasmids (with pBabe-puro as EV control), 100 ng pcDNA3-OTULIN plasmids (with pcDNA3 as EV control), or 10 ng pcDNA3-GFP-SUB plasmids, in a mixture with 20 μl OptiMEM and 1 μl FuGENE HD or FuGENE 6 (Promega E2691) per well. Stimulations were applied immediately after transfection where indicated. Following 24 h incubation, cells were lysed in 75 μl 1× Passive Lysis Buffer (provided in Promega E1960). Luminescence intensity was measured from 10 μl aliquots in technical duplicates using 50 μl of each luciferase assay reagent from the kit.

NF-κB induction levels were calculated by dividing the value of NF-κB luciferase activity by the value of control Renilla luciferase activity. Where indicated, NF-κB induction levels were further normalized to one reference condition.

Expression levels of proteins of interest in the passive lysis buffer lysates were determined by Western blotting as indicated.

### Transwell migration assays

For transwell migration assays, HT29/Tet-On-ZBP1 cells were stimulated in FBS-free McCoy's 5A media supplemented with 0.5% BSA (Sigma A9647, HT29 chemotaxis buffer). Supernatant was collected by centrifuging at 300 *g* for 5 min or by filtering through 0.2 μm CA syringe filter. THP1 or primary neutrophils were resuspended in FBS-free RPMI media supplemented with 0.5% BSA to an approximate of 1 million/ml, and 0.1 ml were plated into each upper chamber of a 24-well plate (Corning 2421). A 0.5 ml conditioned media or HT29 chemotaxis buffer were plated into the lower chamber. The chambers were incubated at 37°C, 5% $CO_2$ for 3 h or as indicated in the figure. Migrated cells were collected from the lower chambers and mixed with Countbright Absolute counting beads (Thermo Fisher C36950) to determine cell density together with the plating population to obtain an accurate plating number. The volume of collected cells was determined by the weight difference of the collection tube before and after collection, assuming density of 1 g/ml. The migration percentage is calculated by dividing migrated cell numbers by plated cell numbers.

### Western blotting

Cells for Western blotting were lysed in 100 μl RIPA buffer (50 mM Tris, pH 7.5, 150 mM NaCl, 1.0% NP-40, 0.5% sodium deoxycholate, 0.1% SDS) supplemented with protease inhibitor (Roche 04693124001) and phosphatase inhibitor cocktails (Roche 4906837001) per well from 6-well plates.

Protein samples were resolved using Bis-Tris gels (10 or 12% acrylamide-bis-acrylamide, 375 mM Tris pH 8.8, 0.1% SDS, 0.1% ammonium persulfate, 0.004% TEMED) in an SDS running buffer (192 mM glycine, 25 mM Tris base, 0.1% SDS) or using NuPAGE™ 4–12% gradient gels (Invitrogen WG1403BX10) in NuPAGE™ MOPS-SDS running buffer (Invitrogen NP0001-02). Separated proteins were transferred to a nitrocellulose membrane (GE 10600002) or PVDF membrane (Millipore IPVH00010). Membranes were blocked for a minimum of 0.5 h at room temperature in a 5% (w/v) skimmed milk solution in PBST (1.47 mM $KH_2PO_4$, 2.68 mM KCl, 136.9 mM NaCl, 7.97 mM $Na_2HPO_4$, 0.1% Tween-20) with 0.2% sodium azide before incubated in the respective primary antibody overnight at 4°C. Membranes were then washed 3 times for 10 min/time with PBST, incubated with secondary antibody diluted 1:5,000–1:10,000 in PBST for 1 h, and washed another 3 times for 10 min/time with PBST. Membranes were developed with Amersham™ ECL Western blotting reagent (GE Healthcare RPN2209) with 0–20% Amersham™ ECL Select™ reagent depending on the signal intensity (GE Healthcare RPN2235). Where necessary, membranes were stripped in 1× ReBlot Plus Strong Antibody Stripping Buffer (Millipore 2504) for 15 min before blocked again for reblotting.

Primary antibodies used for Western blotting in this study: anti-Actin, clone C4 (Millipore MAB1501), anti-Cleaved caspase-8 (Cell Signaling 9496), anti-Caspase-8 (D35G2; Cell Signaling 4790), anti-Caspase-8 (C15; Adipogen, AG-20B-0057), anti-cIAP1 (Enzo ALX-803-335), anti-CYLD (Cell Signaling 8462), anti-ERK1/2 (Cell Signaling 4695), anti-phospho-ERK1/2 (Cell Signaling 4370), anti-FLIP (Cell Signaling 3210), anti-GFP (Cell Signaling 2555), anti-HOIL-1L (Novus NBP1-88301), anti-HOIP (R&D AF8039), anti-IFIT1 (Cell Signaling 14769), anti-IκBα (Cell Signaling 9242), anti-phospho-IκBα (Cell Signaling 2859), anti-IKKβ (Cell Signaling 8943), anti-JNK (Cell Signaling 9258), anti-phospho-JNK (BD 612540), anti-MLKL (Cell Signaling 14993), anti-phospho-MLKL (Cell Signaling 91689), anti-OTULIN (Cell Signaling 14127), anti-p38 (Cell Signaling 9212), anti-phospho-p38 (Cell Signaling 4511), anti-p65 (Cell Signaling 8242), anti-phospho-p65 (Cell Signaling 3033), anti-RIPK1 (Cell Signaling 3493S), anti-RIPK3 (Cell Signaling 13526), anti-RIPK3 (Santa Cruz, sc-47368), anti-phosphoS227-RIPK3 (Abcam ab209384), anti-SHARPIN (Protein tech 14626-1-AP), anti-SARS-CoV-2 Spike protein (GeneTex GTX632604), anti-TAK1 (Cell Signaling 4505), anti-Ubiquitin (Cell Signaling 3936), anti-Ubiquitin (Cell Signaling, 43124), anti-M1-Ub (kindly provided by David Komander and Rune Busk Damgaard), anti-K63-Ub (Cell signaling 5621), anti-Vinculin (Millipore Sigma V9131-100UL), anti-XIAP (BD 610762), anti-ZBP1 (Cell Signaling 60968). Secondary antibodies used for Western blotting in this study include: anti-Mouse IgG-HRP (Dako P0447), anti-Rabbit IgG-HRP (Bio-Rad 1706515), anti-Sheep IgG-HRP (R&D HAF016), anti-goat IgG-HRP (Santa Cruz sc-2354), anti-human IgG-HRP (Bio-Rad 172-1033) and anti-Rat IgG-HRP (Thermo Fisher 31470).

### Anti-FLAG immunoprecipitation

To perform anti-FLAG immunoprecipitation for ZBP1-associated proteins, cells were plated in 10 cm dishes at a density of 3–4 × $10^6$ cells/dish, 3–6 dishes/condition, and stimulated with 500 ng/ml Dox the next day for 16 h. After stimulation, cells were washed twice with PBS and lysed in 500 μl/dish TBSN buffer (50 mM Tris–HCl, pH 7.5, 150 mM NaCl, 0.5% NP-40) supplemented with protease inhibitor cocktail, phosphatase inhibitor cocktail and 50 mM N-Ethylmaleimide (NEM; Sigma Aldrich E1271) by ice incubation

© 2022 The Authors

for a minimum of 15 min. Lysates were centrifuged at maximum speed for 10 min at 4°C to remove debris pellets. After taking input samples, the lysate supernatant was precleared with 10 µl/dish mouse IgG-Agarose beads (Sigma A0919) for a minimum of 30 min at 4°C with end-over-end rotation, before incubated with 10 µl/dish anti-FLAG M2 agarose beads (Sigma A2220) for a minimum of 2 h with end-over-end rotation at 4°C. After enrichment, beads were washed five times with 1 ml TBSN buffer and eluted in 13.3 µl/dish 2× LSB by shaking at 95°C, 750 rpm, for 10 min.

Western blotting was used to probe for co-immunoprecipitated proteins. Equivalent of eluate materials from one 10 cm dish was loaded on each pull-down lane, and 1% input was loaded for reference except for ZBP1, for which 5% input was loaded.

### Enrichment of Ub-conjugates

To enrich for ubiquitinated proteins, cells were seeded in 10 cm dishes and stimulated as described for FLAG immunoprecipitation. For GST-1xUBA and SUB pulldown, beads were prepared by incubation with recombinant GST-1xUBA or SUB for at least 1 h at 4°C with rotation and washed three times with TUBE lysis buffer (20 mM sodium phosphate buffer, pH 7.4, 1% NP-40, 2 mM EDTA). For GST-1xUBA, 10 µl/dish Glutathione Magnetic Agarose beads (Thermo Fisher 78602) were incubated with 30 µg/dish GST-1xUBA. K63-SUB-bound beads were prepared by incubating 20 µl/dish Streptavidin Magnetic Agarose beads (Thermo Fisher 88817) with 5 µg/dish recombinant biotinylated K63-SUB. For M1-SUB pull-down, 10 µl/dish Glutathione Magnetic Agarose beads were incubated with 50 µg M1-SUB (Fiil *et al*, 2013).

Cells were lysed using 500 µl TUBE lysis buffer supplemented with protease inhibitor cocktail, phosphatase inhibitor cocktail, 50 mM NEM and 1 mM DTT following the same protocol as for FLAG immunoprecipitation. After taking input samples, centrifuged lysate supernatant was incubated with 10 µl/dish GST-1xUBA- or SUB-bound beads overnight at 4°C with end-over-end rotation. After enrichment, beads were washed three times with TUBE lysis buffer and heated in 2xLSB for 10 min at 95°C for elution.

Equivalent of pull-down samples from one 10 cm dish and 1% input (except for ubiquitin blot, for which 5% input was loaded) were resolved on 4–12% NuPAGE gradient gels in NuPAGE MOPS-SDS running buffer and transferred onto PVDF membrane for Western blotting.

### On-bead deubiquitinase treatment

On-bead deubiquitinase digestion was performed to probe for the existence and composition of ubiquitin chains in FLAG-immunoprecipitated or TUBE-enriched sections. After enrichment, beads were washed three times with lysis buffer and incubated shaking with or without 1 µM USP21 (R&D E-622-050) in 30 µl DUB buffer (20 mM HEPES, pH 7.5, 100 mM NaCl, 1 mM $MnCl_2$, 0.01% w/v Brij-35, 5 mM DTT) for 1 h at 30°C before LSB buffer was added to end the reaction.

### Biosafety

All experiments with SARS-CoV2 live virus were performed at the Biosafety level 2+ (BSL2+) laboratory (Lab ID: 230465) at Aarhus University, Denmark. The laboratory and work with live virus SARS-CoV2 was approved by the Danish workplace environment authority (Arbejdstilsynet, file number: 2020039772/2). All samples were handled and inactivated according to the approved SOPs.

### Quantification and statistical analysis

#### *SARS-CoV-2 single cell RNA-seq expression analysis*

Processed single cell RNA TPM expression associated with GSE158055 were downloaded and analyzed from http://covid19.cancer-pku.cn/#/summary (Ren *et al*, 2021a, Data ref: Ren *et al*, 2021b). The patient TPM expression of a gene of interest was computed as the mean TPM expression of the patients' individual constituent single cells.

#### *Statistical analysis for quantitative experiments*

Statistical analyses were carried out in GraphPad Prism. The statistical details are described in figure legends, with $n$ representing the number of biological replicates. Outliers were identified by ROUT method (Q = 1%) and excluded from statistical analyses.

For the comparison between two conditions, an F-test was used to test for the statistical differences between the standard deviations (SDs) of the two conditions. A two-tailed unpaired $t$-test was used when SDs from the two conditions were not significantly different. A Welch's $t$-test was used if SDs from the two samples are significantly different.

For comparison of more than two conditions, a Brown-Forsythe test was used to test for statistical differences between the SDs of all conditions. When the Brown-Forsythe test found no significant differences between group variances, one-way ANOVA and Sidak's multiple comparisons test were used to test for statistical differences between indicated conditions. When the Brown-Forsythe test found a significant difference between group variances, Brown-Forsythe and Welch ANOVA tests and Dunnet's T3 multiple comparisons test were used. Multiple $t$-tests were used when SDs from conditions were found to be significantly different and Brown-Forsythe and Welch ANOVA tests were not possible.

For grouped data or data from multifactor experiment designs, two-way ANOVA and the following multiple comparison tests were used following Prism's recommendation on the choice of multiple comparison tests.

The distribution of the single-cell RNA sequencing datasets were found significantly different from Gaussian distribution, and therefore they were analyzed using Kruskai-Wallis test and Dunn's multiple comparisons test.

## Data availability

No primary datasets have been generated and deposited. Plasmids and cell lines generated in this study are available from lead contact with a completed Materials Transfer Agreement.

**Expanded View** for this article is available online.

### Acknowledgments

This work was supported by the Ludwig Institute for Cancer Research Ltd. Work in the M.G-H. lab was supported by a Wellcome Trust Fellowship


© 2022 The Authors

(102894/Z/13/Z and 215612/Z/19/Z), the LEO foundation (from October 2020), and The Novo Nordisk Foundation (NNF20OC0059392, from December 2021). R.P. was supported by a China Scholarship Council-University of Oxford DPhil Scholarship (2015-2019; GAF1516_CSCUO_841725). S.R.P. was funded by The Independent Research Fund Denmark (0214-00001B) the European Research Council (ERC-AdG ENVISION; 786602), and the Novo Nordisk Foundation (NNF18OC0030274). We acknowledge the contribution to this study made by the Oxford Centre for Histopathology Research and the Oxford Radcliffe Biobank, which are supported by the University of Oxford, the Oxford CRUK Cancer Centre and the NIHR Oxford Biomedical Research Centre (Molecular Diagnostics Theme/Multimodal Pathology Subtheme), and the NIHR CRN Thames Valley network. We thank Prof. David Komander and Prof. Rune Busk Damgaard for providing Met1-Ubiquitin antibody, Dr. Norbert Volkmar and Prof. John Christianson for providing shRNA plasmids targeting mismatch sequences, Tetralogic Pharmaceuticals for Compound A, Professor Georg Kochs, for providing SARS-CoV-2 virus stock, Dr. Amit Shrestha and Prof. Nick La Thangue for providing HL60 cells, and members of the M.G-H. group for helpful suggestions and reading the manuscript.

## Author contributions

**Ruoshi Peng:** Conceptualization; formal analysis; investigation; visualization; writing – original draft; writing – review and editing. **Chris Kedong Wang:** Validation; investigation; visualization; writing – review and editing. **Xuan Wang-Kan:** Investigation; writing – review and editing. **Manja Idorn:** Investigation; writing – review and editing. **Majken Kjær:** Investigation. **Felix Y Zhou:** Formal analysis; writing – review and editing. **Berthe K Fiil:** Investigation. **Frederik Timmermann:** Investigation. **Susana L Orozco:** Resources. **Julia McCarthy:** Resources; writing – review and editing. **Carol S Leung:** Resources; supervision. **Xin Lu:** Supervision; funding acquisition. **Katrin Bagola:** Supervision; writing – review and editing. **Jan Rehwinkel:** Resources. **Andrew Oberst:** Resources. **Jonathan Maelfait:** Resources; writing – review and editing. **Søren R Paludan:** Resources; supervision; funding acquisition; writing – review and editing. **Mads Gyrd-Hansen:** Conceptualization; resources; supervision; funding acquisition; visualization; writing – original draft; project administration; writing – review and editing.

## Disclosure and competing interests statement

All authors declare they have no conflict of interest relating to this study.

# References

Blanco-Melo D, Nilsson-Payant BE, Liu WC, Uhl S, Hoagland D, Møller R, Jordan TX, Oishi K, Panis M, Sachs D *et al* (2020a) Imbalanced host response to SARS-CoV-2 drives development of COVID-19. *Cell* 181: 1036 – 1045

Blanco-Melo D, Nilsson-Payant BE, Liu WC, Uhl S, Hoagland D, Møller R, Jordan TX, Oishi K, Panis M, Sachs D *et al* (2020b) Gene Expression Omnibus GSE147507. (https://www.ncbi.nlm.nih.gov/geo/query/acc.cgi?acc=GSE147507) [DATASET]

Cho YS, Challa S, Moquin D, Genga R, Ray TD, Guildford M, Chan FKM (2009) Phosphorylation-driven assembly of the RIP1-RIP3 complex regulates programmed necrosis and virus-induced inflammation. *Cell* 137: 1112 – 1123

Christopher JA, Avitabile BG, Bamborough P, Champigny AC, Cutler GJ, Dyos SL, Grace KG, Kerns JK, Kitson JD, Mellor GW *et al* (2007) The discovery of 2-amino-3,5-diarylbenzamide inhibitors of IKK-α and IKK-β kinases. *Bioorg Med Chem Lett* 17: 3972 – 3977

Clackson T, Yang W, Rozamus LW, Hatada M, Amara JF, Rollins CT, Stevenson LF, Magari SR, Wood SA, Courage NL *et al* (1998) Redesigning an FKBP-ligand interface to generate chemical dimerizers with novel specificity. *Proc Natl Acad Sci U S A* 95: 10437 – 10442

Damgaard RB, Nachbur U, Yabal M, Wong WWL, Fiil BK, Kastirr M, Rieser E, Rickard JA, Bankovacki A, Peschel C *et al* (2012) The ubiquitin ligase XIAP recruits LUBAC for NOD2 signaling in inflammation and innate immunity. *Mol Cell* 46: 746 – 758

Damgaard RB, Fiil BK, Speckmann C, Yabal M, zur Stadt U, Bekker-Jensen S, Jost PJ, Ehl S, Mailand N, Gyrd-Hansen M (2013) Disease-causing mutations in the XIAP BIR2 domain impair NOD2-dependent immune signalling. *EMBO Mol Med* 5: 1278 – 1295

Daniels BP, Kofman SB, Smith JR, Gale M, Loo Y-M, Oberst A (2018) The nucleotide sensor ZBP1 and kinase RIPK3 induce the enzyme IRG1 to promote an antiviral metabolic state in neurons. *Immunity* 50: 64 – 76

De Groote P, Grootjans S, Lippens S, Eichperger C, Leurs K, Kahr I, Tanghe G, Bruggeman I, De Schamphelaire W, Urwyler C *et al* (2016) Generation of a new gateway-compatible inducible lentiviral vector platform allowing easy derivation of co-transduced cells. *Biotechniques* 60: 252 – 259

Devos M, Tanghe G, Gilbert B, Dierick E, Verheirstraeten M, Nemegeer J, de Reuver R, Lefebvre S, De Munck J, Rehwinkel J *et al* (2020) Sensing of endogenous nucleic acids by ZBP1 induces keratinocyte necroptosis and skin inflammation. *J Exp Med* 217: e20191913

Ea CK, Deng L, Xia ZP, Pineda G, Chen ZJ (2006) Activation of IKK by TNFα requires site-specific ubiquitination of RIP1 and polyubiquitin binding by NEMO. *Mol Cell* 22: 245 – 257

Feng S, Yang Y, Mei Y, Ma L, Zhu D e, Hoti N, Castanares M, Wu M (2007) Cleavage of RIP3 inactivates its caspase-independent apoptosis pathway by removal of kinase domain. *Cell Signal* 19: 2056 – 2067

Fiil BK, Damgaard RB, Wagner SA, Keusekotten K, Fritsch M, Bekker-Jensen S, Mailand N, Choudhary C, Komander D, Gyrd-Hansen M (2013) OTULIN restricts Met1-linked ubiquitination to control innate immune signaling. *Mol Cell* 50: 818 – 830

Fougeroux C, Goksøyr L, Idorn M, Soroka V, Myeni SK, Dagil R, Janitzek CM, Søgaard M, Aves KL, Horsted EW *et al* (2021) Capsid-like particles decorated with the SARS-CoV-2 receptor-binding domain elicit strong virus neutralization activity. *Nat Commun* 12: 324

Haas TL, Emmerich CH, Gerlach B, Schmukle AC, Cordier SM, Rieser E, Feltham R, Vince J, Warnken U, Wenger T *et al* (2009) Recruitment of the linear ubiquitin chain assembly complex stabilizes the TNF-R1 signaling complex and is required for TNF-mediated gene induction. *Mol Cell* 36: 831 – 844

Hasegawa M, Fujimoto Y, Lucas PC, Nakano H, Fukase K, Núñez G, Inohara N (2008) A critical role of RICK/RIP2 polyubiquitination in nod-induced NF-κB activation. *EMBO J* 27: 373 – 383

He S, Wang X (2018) RIP kinases as modulators of inflammation and immunity. *Nat Immunol* 19: 912 – 922

He S, Wang L, Miao L, Wang T, Du F, Zhao L, Wang X (2009) Receptor interacting protein Kinase-3 determines cellular necrotic response to TNF-α. *Cell* 137: 1100 – 1111

Hoffmann M, Kleine-Weber H, Schroeder S, Krüger N, Herrler T, Erichsen S, Schiergens TS, Herrler G, Wu NH, Nitsche A *et al* (2020) SARS-CoV-2 cell entry depends on ACE2 and TMPRSS2 and is blocked by a clinically proven protease inhibitor. *Cell* 181: 271 – 280

Hrdinka M, Gyrd-Hansen M (2017) The Met1-linked ubiquitin machinery: emerging themes of (De)regulation. *Mol Cell* 68: 265 – 280

Hrdinka M, Fiil BK, Zucca M, Leske D, Bagola K, Yabal M, Elliott PR, Damgaard RB, Komander D, Jost PJ *et al* (2016) CYLD limits Lys63- and Met1-linked

© 2022 The Authors 

ubiquitin at receptor complexes to regulate innate immune signaling. *Cell Rep* 14: 2846 – 2858

Hrdinka M, Schlicher L, Dai B, Pinkas DM, Bufton JC, Picaud S, Ward JA, Rogers C, Suebsuwong C, Nikhar S *et al* (2018) Small molecule inhibitors reveal an indispensable scaffolding role of RIPK2 in NOD2 signaling. *EMBO J* 37: e99372

Hubbard NW, Ames JM, Maurano M, Chu LH, Somfleth KY, Gokhale NS, Werner M, Snyder JM, Lichauco K, Savan R *et al* (2022) ADAR1 mutation causes ZBP1-dependent immunopathology. *Nature* 607: 769 – 775

Jiao H, Wachsmuth L, Kumari S, Schwarzer R, Lin J, Eren RO, Fisher A, Lane R, Young GR, Kassiotis G *et al* (2020) Z-nucleic-acid sensing triggers ZBP1-dependent necroptosis and inflammation. *Nature* 580: 391 – 395

Jiao H, Wachsmuth L, Wolf S, Lohmann J, Nagata M, Kaya GG, Oikonomou N, Kondylis V, Rogg M, Diebold M *et al* (2022) ADAR1 averts fatal type I interferon induction by ZBP1. *Nature* 607: 776 – 783

Kaiser WJ, Upton JW, Mocarski ES (2008) Receptor-interacting protein homotypic interaction motif-dependent control of NF-κB activation via the DNA-dependent activator of IFN regulatory factors. *J Immunol* 181: 6427 – 6434

Kaiser WJ, Sridharan H, Huang C, Mandal P, Upton JW, Gough PJ, Sehon CA, Marquis RW, Bertin J, Mocarski ES (2013) Toll-like receptor 3-mediated necrosis via TRIF, RIP3, and MLKL. *J Biol Chem* 288: 31268 – 31279

Kesavardhana S, Kuriakose T, Guy CS, Samir P, Malireddi RKS, Mishra A, Kanneganti T-D (2017) ZBP1/DAI ubiquitination and sensing of influenza vRNPs activate programmed cell death. *J Exp Med* 214: 2217 – 2229

Koziczak-Holbro M, Glück A, Tschopp C, Mathison JC, Gram H (2008) Irak-4 kinase activity-dependent and -independent regulation of lipopolysaccharide-inducible genes. *Eur J Immunol* 38: 788 – 796

Kuriakose T, Kanneganti TD (2018) ZBP1: Innate sensor regulating cell death and inflammation. *Trends Immunol* 39: 123 – 134

Kuriakose T, Man SM, Malireddi RKS, Karki R, Kesavardhana S, Place DE, Neale G, Vogel P, Kanneganti T-D (2016) ZBP1/DAI is an innate sensor of influenza virus triggering the NLRP3 inflammasome and programmed cell death pathways. *Sci Immunol* 1: aag2045

Li J, McQuade T, Siemer AB, Napetschnig J, Moriwaki K, Hsiao YS, Damko E, Moquin D, Walz T, McDermott A *et al* (2012) The RIP1/RIP3 necrosome forms a functional amyloid signaling complex required for programmed necrosis. *Cell* 150: 339 – 350

Li S, Zhang Y, Guan Z, Li H, Ye M, Chen X, Shen J, Zhou Y, Shi ZL, Zhou P *et al* (2020) SARS-CoV-2 triggers inflammatory responses and cell death through caspase-8 activation. *Signal Transduct Target Ther* 5: 1 – 10

Li M, Ferretti M, Ying B, Descamps H, Lee E, Dittmar M, Lee JS, Whig K, Kamalia B, Dohnalová L *et al* (2021) Pharmacological activation of STING blocks SARS-CoV-2 infection. *Sci Immunol* 6: eabi9007

Lin J, Kumari S, Kim C, Van TM, Wachsmuth L, Polykratis A, Pasparakis M (2016) RIPK1 counteracts ZBP1-mediated necroptosis to inhibit inflammation. *Nature* 540: 124 – 128

Maelfait J, Liverpool L, Bridgeman A, Ragan KB, Upton JW, Rehwinkel J (2017) Sensing of viral and endogenous RNA by ZBP1/DAI induces necroptosis. *EMBO J* 36: 2529 – 2543

Micheau O, Thome M, Schneider P, Holler N, Tschopp J, Nicholson DW, Briand C, Grütter MG (2002) The long form of FLIP is an activator of caspase-8 at the Fas death-inducing signaling complex. *J Biol Chem* 277: 45162 – 45171

Muendlein HI, Sarhan J, Liu BC, Connolly WM, Schworer SA, Smirnova I, Tang AY, Ilyukha V, Pietruska J, Tahmasebi S *et al* (2020) Constitutive interferon attenuates RIPK1/3-mediated cytokine translation. *Cell Rep* 30: 699 – 713

Muendlein HI, Connolly WM, Zoie M, David J, Irina S, Alexei D, Siddharth B, Alexander P (2022) ZBP1 promotes inflammatory responses downstream of TLR3/TLR4 via timely delivery of RIPK1 to TRIF. *Proc Natl Acad Sci U S A* 119: e2113872119

Najjar M, Saleh D, Zelic M, Nogusa S, Shah S, Tai A, Finger JN, Polykratis A, Gough PJ, Bertin J *et al* (2016) RIPK1 and RIPK3 kinases promote cell-death-independent inflammation by toll-like receptor 4. *Immunity* 45: 46 – 59

Newton K (2020) Multitasking kinase RIPK1 regulates cell death and inflammation. *Cold Spring Harb Perspect Med* 12: a036368

Newton K, Sun X, Dixit VM (2004) Kinase RIP3 is dispensable for Normal NF-κBs, signaling by the B-cell and T-cell receptors, tumor necrosis factor receptor 1, and toll-like receptors 2 and 4. *Mol Cell Biol* 24: 1464 – 1469

Newton K, Wickliffe KE, Maltzman A, Dugger DL, Strasser A, Pham VC, Lill JR, Roose-Girma M, Warming S, Solon M *et al* (2016) RIPK1 inhibits ZBP1-driven necroptosis during development. *Nature* 540: 129 – 133

Newton K, Wickliffe KE, Dugger DL, Maltzman A, Roose-Girma M, Dohse M, Kőműves L, Webster JD, Dixit VM (2019) Cleavage of RIPK1 by caspase-8 is crucial for limiting apoptosis and necroptosis. *Nature* 574: 428 – 431

Oberst A, Dillon CP, Weinlich R, McCormick LL, Fitzgerald P, Pop C, Hakem R, Salvesen GS, Green DR (2011) Catalytic activity of the caspase-8-FLIP L complex inhibits RIPK3-dependent necrosis. *Nature* 471: 363 – 368

O'Donnell MA, Perez-Jimenez E, Oberst A, Ng A, Massoumi R, Xavier R, Green DR, Ting AT (2011) Caspase 8 inhibits programmed necrosis by processing CYLD. *Nat Cell Biol* 13: 1437 – 1442

Olagnier D, Farahani E, Thyrsted J, Blay-Cadanet J, Herengt A, Idorn M, Hait A, Hernaez B, Knudsen A, Iversen MB *et al* (2020) SARS-CoV2-mediated suppression of NRF2-signaling reveals potent antiviral and anti-inflammatory activity of 4-octyl-itaconate and dimethyl fumarate. *Nat Commun* 11: 4938

Omoto S, Guo H, Talekar GR, Roback L, Kaiser WJ, Mocarski ES (2015) Suppression of RIP3-dependent necroptosis by human cytomegalovirus. *J Biol Chem* 290: 11635 – 11648

Ordureau A, Smith H, Windheim M, Peggie M, Carrick E, Morrice N, Cohen P (2008) The Irak-catalysed activation of the E3 ligase function of Pellino isoforms induces the Lys63-linked polyubiquitination of IRAK1. *Biochem J* 409: 43 – 52

Orozco S, Yatim N, Werner MR, Tran H, Gunja SY, Tait SWG, Albert ML, Green DR, Oberst A (2014) RIPK1 both positively and negatively regulates RIPK3 oligomerization and necroptosis. *Cell Death Differ* 21: 1511 – 1521

Orozco SL, Daniels BP, Yatim N, Messmer MN, Quarato G, Chen-Harris H, Cullen SP, Snyder AG, Ralli-Jain P, Frase S *et al* (2019) RIPK3 activation leads to cytokine synthesis that continues after loss of cell membrane integrity. *Cell Rep* 28: 2275 – 2287.e5

Pham TH, Kwon KM, Kim Y-E, Kim KK, Ahn J-H (2013) DNA sensing-independent inhibition of herpes simplex virus 1 replication by DAI/ZBP1. *J Virol* 87: 3076 – 3086

Rebsamen M, Heinz LX, Meylan E, Michallet MC, Schroder K, Hofmann K, Vazquez J, Benedict CA, Tschopp J (2009) DAI/ZBP1 recruits RIP1 and RIP3 through RIP homotypic interaction motifs to activate NF-κB. *EMBO Rep* 10: 916 – 922

Ren X, Wen W, Fan X, Hou W, Su B, Cai P, Li J, Liu Y, Tang F, Zhang F *et al* (2021a) COVID-19 immune features revealed by a large-scale single cell transcriptome atlas. *Cell* 184: 1815 – 1913

Ren X, Wen W, Fan X, Hou W, Su B, Cai P, Li J, Liu Y, Tang F, Zhang F *et al* (2021b) Gene Expression Omnibus GSE158055. (http://covid19.cancer-pku.cn/#/summary). [DATASET]


 © 2022 The Authors

de Reuver R, Verdonck S, Dierick E, Nemegeer J, Hessmann E, Ahmad S, Jans M, Blancke G, Van Nieuwerburgh F, Botzki A *et al* (2022) ADAR1 prevents autoinflammation by suppressing spontaneous ZBP1 activation. *Nature* 607: 784 – 789

Rodriguez DA, Weinlich R, Brown S, Guy C, Fitzgerald P, Dillon CP, Oberst A, Quarato G, Low J, Cripps JG *et al* (2016) Characterization of RIPK3-mediated phosphorylation of the activation loop of MLKL during necroptosis. *Cell Death Differ* 23: 76 – 88

Saksela K, Baltimore D (1993) Negative regulation of immunoglobulin kappa light-chain gene transcription by a short sequence homologous to the murine B1 repetitive element. *Mol Cell Biol* 13: 3698 – 3705

Schmittgen TD, Livak KJ (2008) Analyzing real-time PCR data by the comparative CT method. *Nat Protoc* 3: 1101 – 1108

Sims JJ, Scavone F, Cooper EM, Kane LA, Youle RJ, Boeke JD, Cohen RE (2012) Polyubiquitin-sensor proteins reveal localization and linkage-type dependence of cellular ubiquitin signaling. *Nat Methods* 9: 303 – 309

Singh A, Sweeney MF, Yu M, Burger A, Greninger P, Benes C, Haber DA, Settleman J (2012) TAK1 inhibition promotes apoptosis in KRAS-dependent colon cancers. *Cell* 148: 639 – 650

Sun X, Lee J, Navas T, Baldwin DT, Stewart TA, Dixit VM (1999) RIP3, a novel apoptosis-inducing kinase. *J Biol Chem* 274: 16871 – 16875

Sun L, Wang H, Wang Z, He S, Chen S, Liao D, Wang L, Yan J, Liu W, Lei X *et al* (2012) Mixed lineage kinase domain-like protein mediates necrosis signaling downstream of RIP3 kinase. *Cell* 148: 213 – 227

Takaoka A, Wang Z, Choi MK, Yanai H, Negishi H, Ban T, Lu Y, Miyagishi M, Kodama T, Honda K *et al* (2007) DAI (DLM-1/ZBP1) is a cytosolic DNA sensor and an activator of innate immune response. *Nature* 448: 501 – 505

Thapa RJ, Ingram JP, Ragan KB, Nogusa S, Boyd DF, Benitez AA, Sridharan H, Kosoff R, Shubina M, Landsteiner VJ *et al* (2016) DAI senses influenza a virus genomic RNA and activates RIPK3-dependent cell death. *Cell Host Microbe* 20: 674 – 681

Topal Y, Gyrd-Hansen M (2021) RIPK2 NODs to XIAP and IBD. *Semin Cell Dev Biol* 109: 144 – 150

Upton JW, Kaiser WJ, Mocarski ES (2012) DAI/ZBP1/DLM-1 complexes with RIP3 to mediate virus-induced programmed necrosis that is targeted by murine cytomegalovirus vIRA. *Cell Host Microbe* 11: 290 – 297

Vince JE, Wong WWL, Khan N, Feltham R, Chau D, Ahmed AU, Benetatos CA, Chunduru SK, Condon SM, McKinlay M *et al* (2007) IAP antagonists target cIAP1 to induce TNFα-dependent apoptosis. *Cell* 131: 682 – 693

Waelchli R, Bollbuck B, Bruns C, Buhl T, Eder J, Feifel R, Hersperger R, Janser P, Revesz L, Zerwes HG *et al* (2006) Design and preparation of 2-benzamido-pyrimidines as inhibitors of IKK. *Bioorg Med Chem Lett* 16: 108 – 112

Wang R, Li H, Wu J, Cai Z-Y, Li B, Ni H, Qiu X, Chen H, Liu W, Yang Z-H *et al* (2020) Gut stem cell necroptosis by genome instability triggers bowel inflammation. *Nature* 580: 386 – 390

van Wijk SJL, Fiskin E, Putyrski M, Pampaloni F, Hou J, Wild P, Kensche T, Grecco HE, Bastiaens P, Dikic I (2012) Fluorescence-based sensors to monitor localization and functions of linear and K63-linked ubiquitin chains in cells. *Mol Cell* 47: 797 – 809

Wu J, Powell F, Larsen NA, Lai Z, Byth KF, Read J, Gu RF, Roth M, Toader D, Saeh JC *et al* (2013) Mechanism and *in vitro* pharmacology of TAK1 inhibition by (5Z)-7-oxozeaenol. *ACS Chem Biol* 8: 643 – 650

Wu XN, Yang ZH, Wang XK, Zhang Y, Wan H, Song Y, Chen X, Shao J, Han J (2014) Distinct roles of RIP1-RIP3 hetero-and RIP3-RIP3 homo-interaction in mediating necroptosis. *Cell Death Differ* 21: 1709 – 1720

Wyler E, Mösbauer K, Franke V, Diag A, Gottula LT, Arsiè R, Klironomos F, Koppstein D, Hönzke K, Ayoub S *et al* (2021) Transcriptomic profiling of SARS-CoV-2 infected human cell lines identifies HSP90 as target for COVID-19 therapy. *iScience* 24: 102151

Yatim N, Jusforgues-Saklani H, Orozco S, Schulz O, Da Silva RB, Reis E Sousa C, Green DR, Oberst A, Albert ML (2015) RIPK1 and NF-κB signaling in dying cells determines cross-priming of CD8+ T cells. *Science* 350: 328 – 334

Yu PW, Huang BCB, Shen M, Quast J, Chan E, Xu X, Nolan GP, Payan DG, Luo Y (1999) Identification of RIP3, a RIP-like kinase that activates apoptosis and NFκB. *Curr Biol* 9: 539 – 542

Zhang T, Yin C, Boyd DF, Quarato G, Ingram JP, Shubina M, Ragan KB, Ishizuka T, Crawford JC, Tummers B *et al* (2020) Influenza virus Z-RNAs induce ZBP1-mediated necroptosis. *Cell* 180: 1115 – 1129

Zhang T, Yin C, Fedorov A, Qiao L, Bao H, Beknazarov N, Wang S, Gautam A, Williams RM, Crawford JC *et al* (2022) ADAR1 masks the cancer immunotherapeutic promise of ZBP1-driven necroptosis. *Nature* 606: 594 – 602

Zhu K, Liang W, Ma Z, Xu D, Cao S, Lu X, Liu N, Shan B, Qian L, Yuan J (2018) Necroptosis promotes cell-autonomous activation of proinflammatory cytokine gene expression. *Cell Death Dis* 9: 500

**License:** This is an open access article under the terms of the Creative Commons Attribution License, which permits use, distribution and reproduction in any medium, provided the original work is properly cited.

# Expanded View Figures

**Figure EV1. ZBP1 induces inflammatory signaling independently of cell death.**

A Western blot analysis of ZBP1 levels in HT29, HT29/TO-ZBP1$^{WT}$, and HT29/TO-ZBP1$^{Z\alpha1\alpha2mut}$ cells treated or not with Dox or IFNβ (10 ng/ml) for 24 h. Blots are representative of two biological replicates.

B Cell death analysis of HT29 and HT29/TO-ZBP1 cells as indicated following 48 h treatment with Dox using SytoxGreen to stain dead cells. Data are presented as mean ± S.E.M ($n = 5$ biological replicates). One-way ANOVA and Sidak's multiple comparisons tests were used to test for the statistical differences between indicated conditions and untreated condition of the same cell line. *$P = 0.0283$; ****$P < 0.0001$.

C Relative viability of HT29/TO-ZBP1$^{WT}$ and HT29/TO-ZBP1$^{Z\alpha1\alpha2mut}$ cells treated with Dox for up to 72 h was determined by the CellTitre-Glo assay. Values were normalized to that of 0 h for each cell line. Data are presented as mean with S.E.M ($n = 3$ biological replicates). Two-way ANOVA and Tukey's multiple comparisons tests were used to test for the statistical differences between different cell lines. n.s. = not significant ($P = 0.9510$); ****$P < 0.0001$.

D Cell death analysis of HT29/TO-ZBP1$^{WT}$ cells by SytoxGreen staining following treatment with TSZ in combination with the indicated inhibitors. Cells were pre-treated with 100 nM LCL161, 20 μM zVAD combined with10 μM GSK′840, 10 μM GSK′872, or 10 μM Nec1s for 1 h followed by treatment with 10 ng/ml TNF. Cell death analysis was carried out at the end of 24 h incubation with all compounds. Data are presented as mean ($n = 2$ biological replicates) with individual data points indicated.

E, F Cytokine concentrations in the culture media of HT29/TO-ZBP1 cells treated with Dox for 24 h. Cells from the same wells were lysed for Western blot to determine ZBP1 expression levels. (E) Data are presented as mean with S.E.M ($n = 6$ biological replicates). Brown-Forsythe and Welch ANOVA tests and Dunnet's T3 multiple comparisons test were used to test for statistical significances between indicated conditions. ***$P = 0.0002$; *$P = 0.0447$; ****$P < 0.0001$; n.s., not significant ($P = 0.2153$). Western blots are representative of six biological replicates. (F) Data are presented as mean with S.E.M ($n = 4$ biological replicates). Unpaired *t*-tests were used to test for the statistical differences between the indicated conditions. *$P = 0.0257$; **$P = 0.0034$. Cell lysates from one biological replicate were analyzed by Western blotting.

G CXCL1 concentration in the culture media from the experiment described in (Fig 1E). Data are presented as mean with S.E.M. ($n = 3$ biological replicates). Two-way ANOVA tests and Sidak's multiple comparisons test were used to test for statistical differences between indicated conditions. n.s., not significant ($P > 0.09$); ****$P < 0.0001$.

H CXCL8 concentration in the culture media of HT29/TO-ZBP1$^{WT}$ cells treated with 0 or 50 ng/ml Dox in combination with DMSO, 10 μM Nec1s, 10 μM GSK′872, or 1 μM NSA for 24 h. Data are plotted as mean with S.E.M. ($n = 4$ biological replicates). One-way ANOVA and Sidak's multiple comparisons tests were used to test for statistical differences between indicated conditions. n.s. = not significant ($P > 0.39$); **$P = 0.0012$; ****$P < 0.0001$; *$P = 0.0297$. Cells from the same wells were analyzed by Western blotting for ZBP1 and RIPK3 levels. Blots are representative of three biological replicates.


© 2022 The Authors

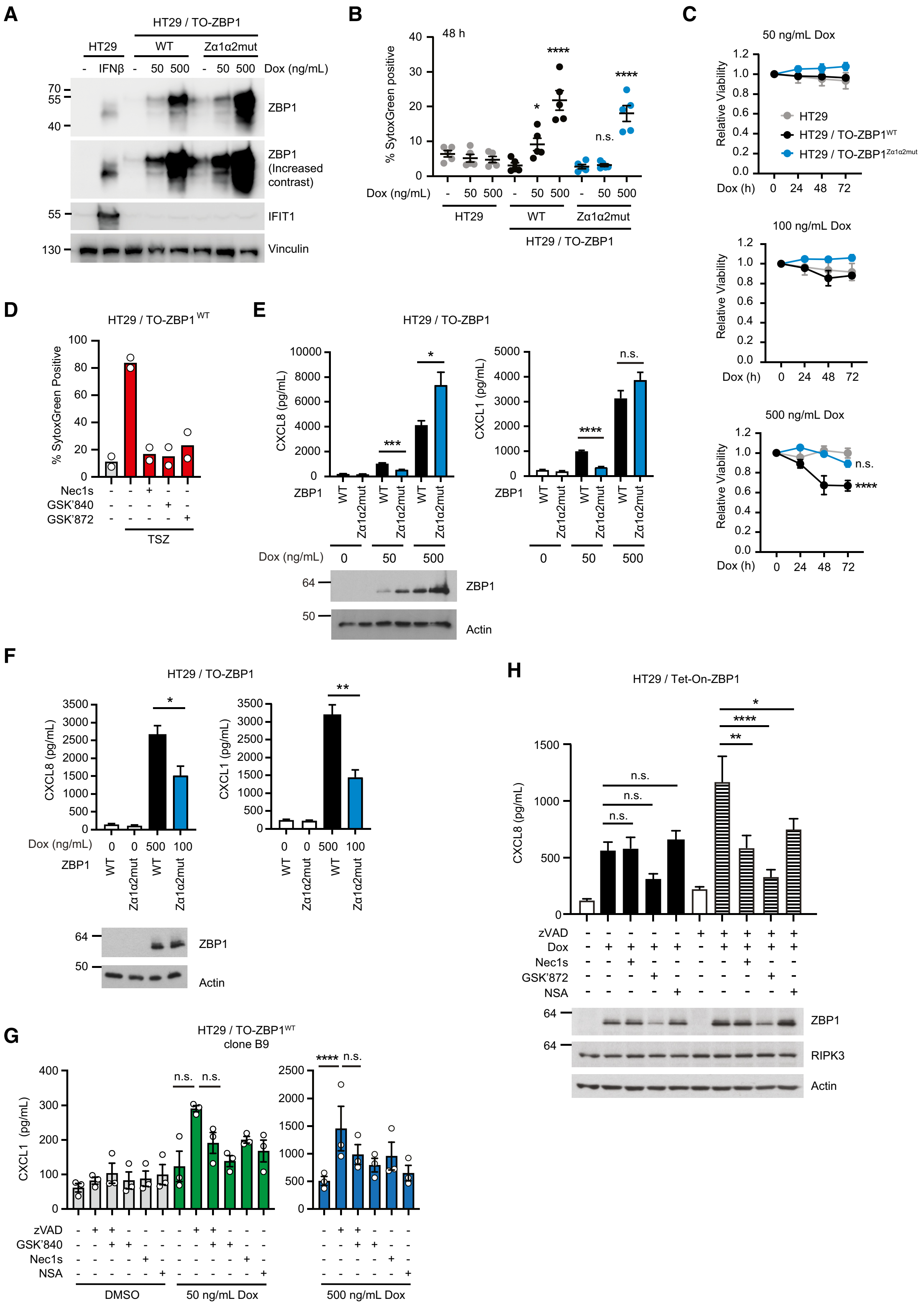
A
HT29 / TO-ZBP1
HT29
WT
Zα1α2mut
- IFNβ - 50 500 - 50 500 Dox (ng/mL)
ZBP1
ZBP1 (Increased contrast)
IFIT1
Vinculin
B
48 h
% SytoxGreen positive
Dox (ng/mL)
HT29
WT
Zα1α2mut
HT29 / TO-ZBP1
n.s.
C
50 ng/mL Dox
100 ng/mL Dox
500 ng/mL Dox
Relative Viability
Dox (h)
HT29
HT29 / TO-ZBP1WT
HT29 / TO-ZBP1Zα1α2mut
D
HT29 / TO-ZBP1WT
% SytoxGreen Positive
Nec1s
GSK'840
GSK'872
TSZ
E
HT29 / TO-ZBP1
CXCL8 (pg/mL)
CXCL1 (pg/mL)
ZBP1
Dox (ng/mL)
Actin
F
HT29 / TO-ZBP1
G
HT29 / TO-ZBP1WT clone B9
CXCL1 (pg/mL)
zVAD
GSK'840
Nec1s
NSA
DMSO
50 ng/mL Dox
500 ng/mL Dox
H
HT29 / Tet-On-ZBP1
CXCL8 (pg/mL)
zVAD
Dox
Nec1s
GSK'872
NSA
ZBP1
RIPK3
Actin

**Figure EV1.**

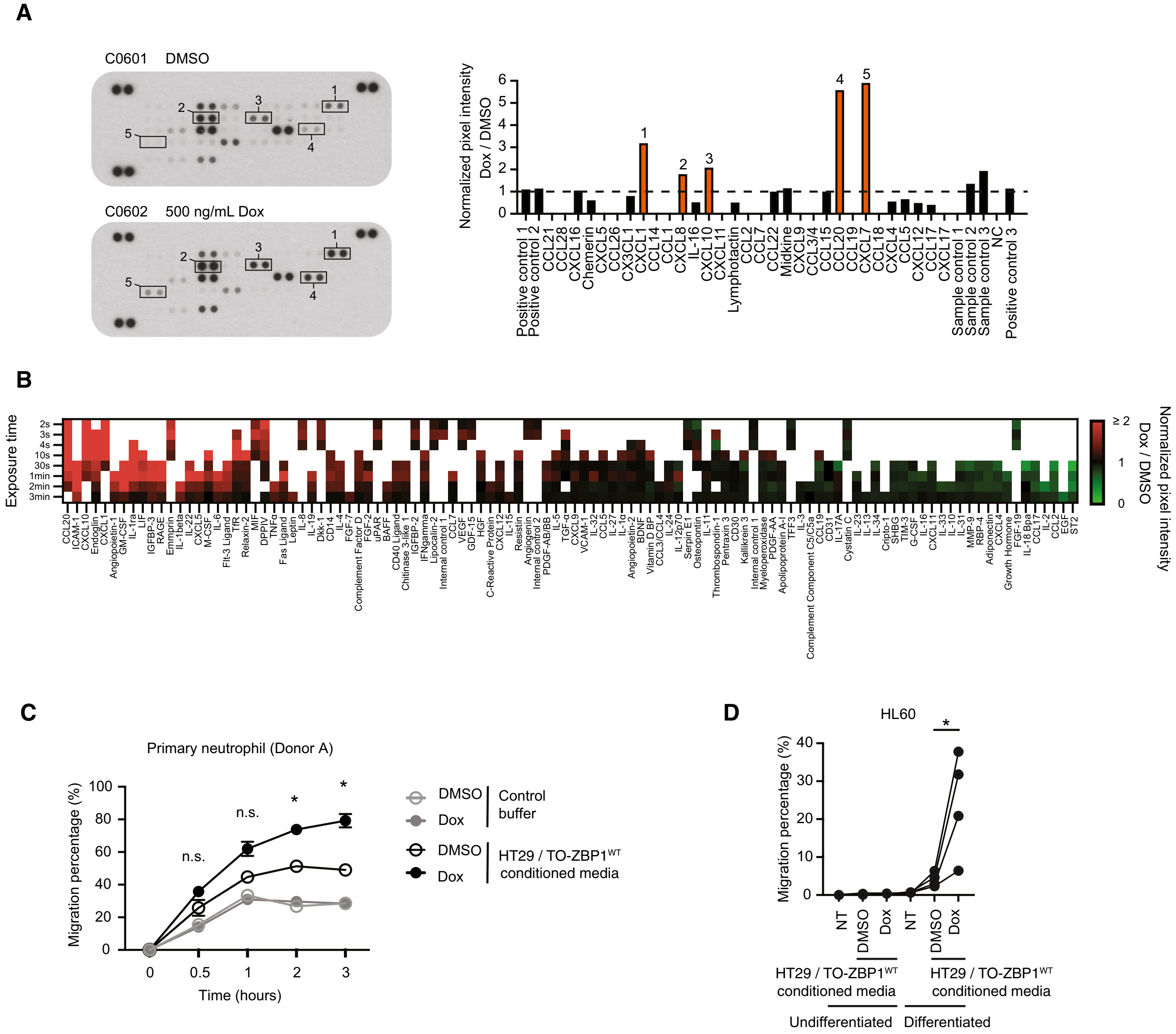


**Figure EV2. ZBP1 expression-induced cytokine secretome promotes the chemotaxis of neutrophils.**

A, B Chemokine (A) and cytokine (B) arrays of the conditioned media from HT29/TO-ZBP1$^{WT}$ cells treated with DMSO or 500 ng/ml Dox for 24 h. Normalized pixel intensity values are presented relative to values from DMSO-treated samples ($n = 1$ biological replicate).

C Transwell migration of primary neutrophils from Donor A towards conditioned media from HT29/TO-ZBP1$^{WT}$ cells treated with DMSO or 500 ng/ml Dox for 24 h, or control buffer containing equal volume and amount of DMSO or Dox as the conditioned media. Data are presented as mean with S.E.M. ($n = 3$ biological replicates). Two-way ANOVA and Dunnet's multiple comparison tests were used to test for statistical differences between the migration of neutrophils toward conditioned media from DMSO-treated cells and that from Dox-treated cells at each time point. n.s. = not significant ($P > 0.05$); *$P = 0.0113$ for the 2 h time point, $P = 0.0133$ for the 3 h time point.

D Transwell migration of differentiated or undifferentiated HL60 cells toward control buffer (NT) or conditioned media from HT29/TO-ZBP1$^{WT}$ cells treated with DMSO or 500 ng/ml Dox for 24 h. Data from each biological replicate of differentiated HL60 cells and conditioned media are connected with solid lines ($n = 4$ biological replicates). A paired $t$-test were used to determine the statistical difference between indicated conditions. *$P = 0.0447$.

 © 2022 The Authors

**Figure EV3. RIPK1 mediates ZBP1-induced inflammatory signaling and cell death.**

A CXCL8 and CXCL1 concentration in the culture media of HT29/TO-ZBP1$^{WT}$ Clone B9 cells following 24 h treatment with 0 or 500 ng/ml Dox in combination with 10 μM GSK'840 or 10 μM GSK'872 as indicated. Data are presented as mean with S.E.M. ($n = 3$ biological replicates). Two-way ANOVA tests and Tukey's multiple comparisons test were used to test for statistical differences between indicated conditions. n.s., not significant ($P \geq 0.05$); *$P = 0.0174$; ***$P = 0.0006$; ****$P < 0.0001$.

B Cytokine concentration in the culture media of HT29/TO-ZBP1$^{WT}$ or two independent clones of HT29/RIPK1-KO/TO-ZBP1$^{WT}$ cells treated with Dox as indicated. N.D., not detected. Data are presented as mean with S.E.M. ($n = 3$ biological replicates). One-way ANOVA and Sidak's multiple comparisons test were used to test for statistical differences between indicated conditions. n.s. = not significant ($P > 0.05$); **$P = 0.0079$ for CXCL1, $P = 0.0012$ for CXCL8; ***$P = 0.0008$; ****$P < 0.0001$. Cell lysates from the same wells were analyzed by Western blotting for ZBP1 and RIPK1 levels. Blots are representative of three biological replicates.

C Relative viability of HT29/TO-ZBP1$^{WT}$ cells or two independent clones of HT29/RIPK1-KO/TO-ZBP1$^{WT}$ cells after treatment with or without 500 ng/ml Dox for up to 3 days as indicated. Viability is normalized to no Dox-treatment within the same cell line. Data are presented as mean with S.E.M. ($n = 3$ biological replicates). Two-way ANOVA and Sidak's multiple comparison tests were used to test for statistical differences between WT and RIPK1-KO cells after Dox treatment. ****$P < 0.0001$.

D Two biological replicates of Ub-conjugate enrichment analysis shown in Fig 3C.

© 2022 The Authors

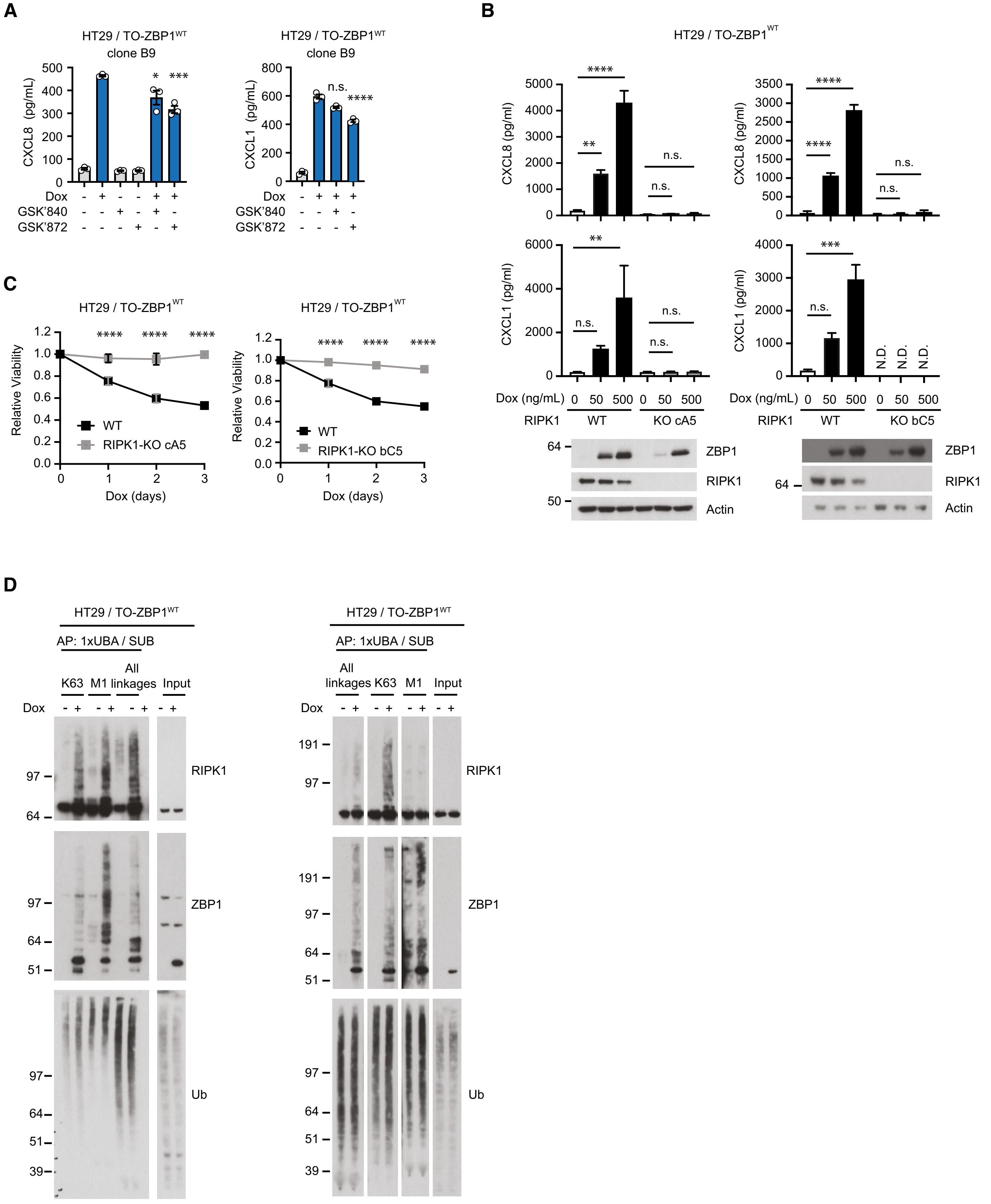


**Figure EV3.**

 © 2022 The Authors

**Figure EV4. RIPK3 oligomerization induces inflammatory signaling.**

A Relative viability of HT29 or HT29/RIPK3-2xFV cells pretreated with combinations of 100 nM CpA, 20 μM zVAD, and 10 μM Nec1s or DMSO (−) as indicated for 1 h, and then treated with or without 2 ng/ml TNF for 24 h. Viability is normalized to the DMSO-only condition for each cell line. Data are presented as mean with S.E.M. ($n = 3$ biological replicates). Two-way ANOVA and Sidak's multiple comparison test were used to test for statistical differences between indicated conditions and DMSO-treated condition of each cell line. **$P = 0.0044$; ****$P < 0.0001$.

B Western blot analysis of necroptosis markers in HT29 cells pretreated with 100 nM CpA and 20 μM zVAD for 1 h before stimulating with 20 ng/ml TNF as indicated. Blots are representative of four biological replicates.

C Western blot analyses of inflammatory marker proteins in HT29/RIPK3-2xFV cells treated with 100 nM dimerizer. Blots are representative of two biological replicates.

D, E Relative *CXCL8* expression (D) and CXCL8 concentration in culture media (E) of HT29/RIPK3-2xFV cells treated with 0 or 100 nM dimerizer as indicated. Data are presented as mean with S.E.M. ($n = 3$ biological replicates). A Welch's $t$-test was used to test for statistical differences between indicated conditions. **$P = 0.0016$ (D); **$P = 0.0093$ (E).

F Relative viability of HT29/RIPK3-2xFV cells treated with 0 or 100 nM dimerizer for 24 h in the presence of 0.5 μg/ml mouse IgG. Data are presented as mean with S.E.M. ($n = 3$ biological replicates). A Welch's $t$-test was used to test for statistical differences between indicated conditions. n.s. = not significant ($P = 0.3268$).

G, H Western blot analysis of (G) HEK293FT/RIPK3-2xFV cells (FL or ΔC) and (H) U2OS/NOD2 cells stably expressing RIPK3 variants treated with 100 nM dimerizer as indicated. Blots are representative of two biological replicates.

I Time course of relative *CXCL8* and *TNF* expression in U2OS/NOD2/RIPK3-2xFV cells (FL or ΔC) treated with 100 nM dimerizer as indicated. Data are plotted as mean with S.E.M. ($n = 3$ biological replicates). Two-way ANOVA and Tukey's multiple comparisons test were used to test for statistical differences between 0 h and indicated time points in FL cells. **$P = 0.0021$ for 2 h, $P = 0.0010$ for 3 h; n.s. = not significant ($P = 0.6323$); ****$P < 0.0001$.

J, K Relative *CXCL8* and *TNF* expression (J) and CXCL8 concentration in the culture media (K) of HT29/RIPK3-2xFV cells pretreated or not with 2 μM CpA for 1 h before treated or not with 100 nM dimerizer for 3 h. Data are plotted as mean with S.E.M. ($n = 3$ biological replicates). Unpaired $t$-tests were used to test for statistical differences between indicated conditions. ****$P < 0.0001$; *$P = 0.0145$ (J). A Welch's $t$-test was used to test for statistical differences between indicated conditions. **$P = 0.0097$ (K).

L Relative *CXCL8* expression in WT or XIAP-knockout HCT116/RIPK3-2xFV cells treated with or without 100 nM dimerizer for 3 h. Data are plotted as mean with S.E.M. ($n = 3$ biological replicates). A Welch's $t$-test were used to test for statistical differences between indicated conditions. n.s. = not significant ($P = 0.2057$). Cell lysates were analyzed by Western blotting. Blots are representative of two biological replicates.

© 2022 The Authors

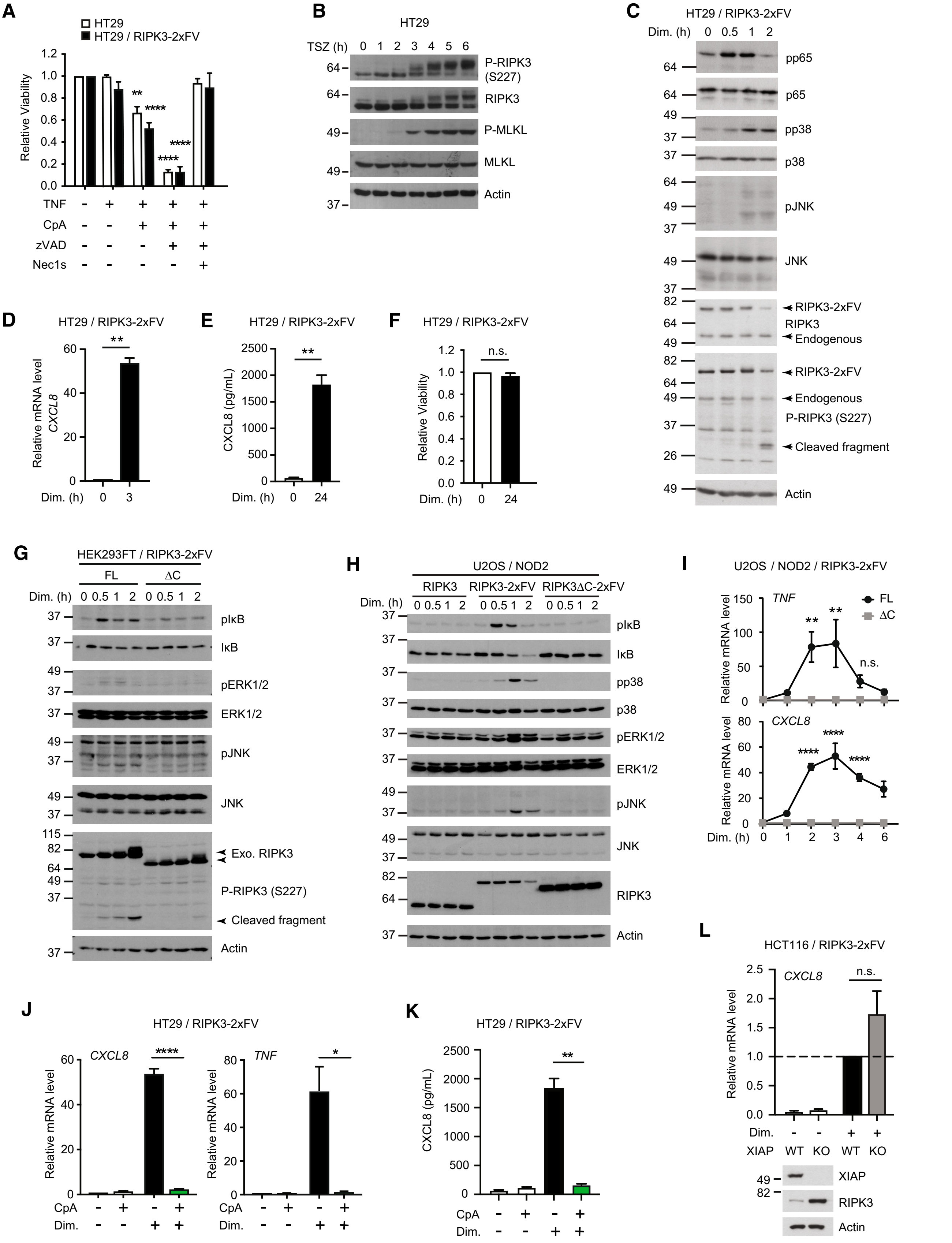


Figure EV4.


© 2022 The Authors

**Figure EV5. ZBP1 mediates SARS-CoV-2-induced inflammation.**

A Relative expression of *ZBP1* in post-mortem lung samples of two COVID-19 patients compared with healthy lung biopsies (Blanco-Melo *et al*, 2020a, Data ref: Blanco-Melo *et al*, 2020b).

B Patient-averaged single cell Transcript Per Million (TPM) values of *ZBP1* in lung and peripheral blood of COVID-19 patients in progressive or convalescent stage, compared to healthy controls (Ren *et al*, 2021a, Data ref: Ren *et al*, 2021b). Data are plotted as individual values per patient with mean and S.E.M. ($n = 25$ for healthy, $n = 77$ for progression, $n = 102$ for convalescence patients). Kruskai–Wallis test and Dunn's multiple comparisons test were used to test for statistical differences between indicated conditions. ****$P < 0.0001$; *$P = 0.0375$; **$P = 0.0036$.

C Pearson's correlation of individual single cell TPM values of indicated genes with virus load (Ren *et al*, 2021a, Data ref: Ren *et al*, 2021b). Data are presented as individual values from each cell, with linear regression line and its 95% confidence bands. *ZBP1*, $n = 720$, *IL*-6, $n = 94$, *TNF*, $n = 211$, *CXCL10*, $n = 1{,}053$, *CXCL8*, $n = 1{,}463$, *ACTB*, $n = 2{,}683$ (cells). The positive correlation is significant ($P < 0.0001$) between TPM (virus) and TPM (*ZBP1*), TPM (*IL*-6), TPM (*TNF*), TPM (*CXCL10*), or TPM (*CXCL8*). Correlation between TPM (virus) and TPM (*ACTB*) is not significant ($P = 0.7862$).

D Fold of mRNA levels of SARS-CoV-2-encoded *N1* and *N2* (C) over β-Actin (ACTB, control), at indicated time after infection in Calu-3 cells, normalized over mock 16 h. Data is presented as mean with S.E.M ($n = 3$ biological replicates). Two-way ANOVA and Tukey's multiple comparison tests were used to test for the statistical differences between each time point with 16 h within the same MOI. n.s., not significant ($P > 0.4$); *$P = 0.0376$; ****$P < 0.0001$.

E Western blot analysis of SARS-CoV-2-infected Calu-3 cells at MOI = 2 for the indicated time. Analysis was performed on one biological replicate.

F, G Expression of *IFNB1* and *IFIT1* (F) or SARS-CoV-2-encoded *N1* and *N2* (G) relative to β-Actin in Calu-3 cells with stable knockdown of *ZBP1* (shZBP1-50, shZBP1-52 and shZBP1-53) and control cells (shMM) infected with mock or SARS-CoV-2 virus at MOI = 2 for 72 h. Data are presented as mean with S.E.M ($n = 3$ biological replicates). One-way ANOVA and Sidak's multiple comparisons test were used to test for statistical differences between indicated conditions. n.s. not significant ($P = 0.3831$); ***$P = 0.0006$ for *IFNB1*, $P = 0.0003$ for *IFIT1*; *$P = 0.0143$; ****$P < 0.0001$ (F) and to test for statistical differences between shMM MOI = 2 and indicated conditions. *$P = 0.0294$ for shZBP1-50, $P = 0.0259$ for shZBP1-53; ***$P = 0.0008$; n.s., not significant ($P > 0.05$) (G).

© 2022 The Authors 

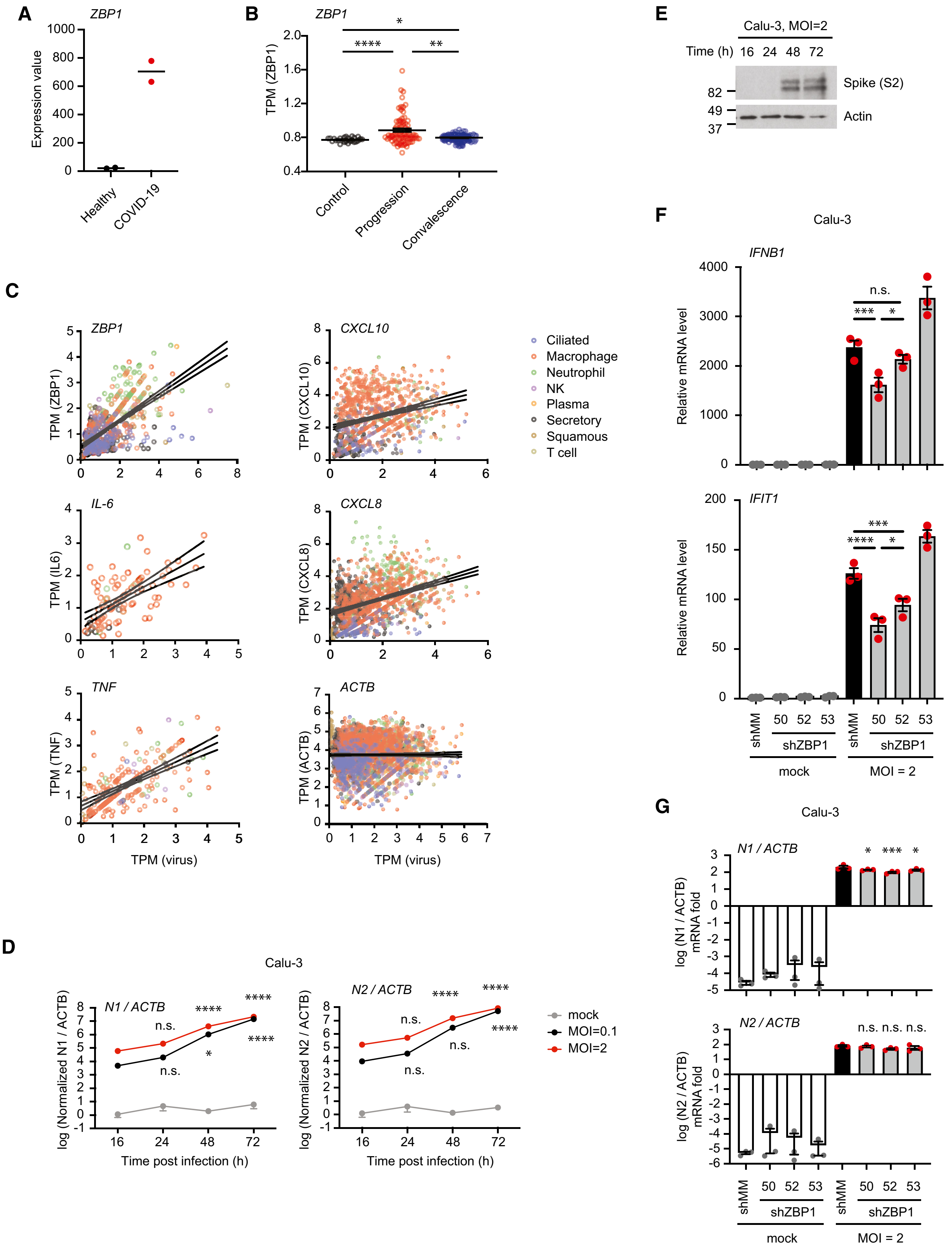


**Figure EV5.**

 © 2022 The Authors
