# Supplementary material for: Human ZBP1 induces cell death‐independent inflammatory signaling via RIPK3 and RIPK1
Source: EMBO Rep. 2022 Oct 21;23(12):e55839. doi: 10.15252/embr.202255839 (PMC9724671; doi:10.15252/embr.202255839)
Supplement: Supplementary file 11 [file EMBR-23-e55839-s009.pdf]

EMBO reports

Human ZBP1 induces cell death-independent inflammatory signaling via RIPK3 and RIPK1

Ruoshi Peng, Chris Wang, Xuan Wang-Kan, Manja Idorn, Majken Kjær, Felix Zhou, Berthe Fiil, Frederik Timmermann, Susana Orozco, Julia McCarthy, Carol Leung, Xin Lu, Katrin Bagola, Jan Rehwinkel, Andrew Oberst, Jonathan Maelfait, Søren Paludan, and Mads Gyrd-Hansen
DOI: 10.15252/embr.202255839

Corresponding author(s): Mads Gyrd-Hansen (mgyrd@sund.ku.dk)

Review Timeline:

Submission Date:	22nd Jul 22
Editorial Decision:	26th Jul 22
Revision Received:	11th Sep 22
Editorial Decision:	30th Sep 22
Revision Received:	6th Oct 22
Accepted:	7th Oct 22

Editor: Achim Breiling

Transaction Report: This manuscript was transferred to EMBO reports following peer review at The EMBO Journal.

(Note: With the exception of the correction of typographical or spelling errors that could be a source of ambiguity, letters and reports are not edited. Depending on transfer agreements, referee reports obtained elsewhere may or may not be included in this compilation. Referee reports are anonymous unless the Referee chooses to sign their reports.)

Dear Prof. Gyrd-Hansen,

Thank you for transferring your manuscript to EMBO reports and for the detailed online discussion regarding the further revisions you suggest. I now went again through your manuscript and the referee reports from The EMBO Journal (attached below again). The referees acknowledge that the findings are of interest, but they have a number of remaining concerns and suggestions to improve the manuscript, or to strengthen the data and the conclusions drawn.

EMBO reports emphasizes novel functional over detailed mechanistic insight, but asks for strong in vivo relevance of the findings, and clear experimental support of the major conclusions. Thus, we will not require addressing points regarding more mechanistic details experimentally. However, it will be necessary that during revision you address all points questioning the main conclusions of the study, and all technical concerns, or points regarding the experimental designs, model systems used, or data presentation, either by the addition of further data, text changes or good arguments in the detailed p-b-p-response I ask you to submit together with your revised manuscript.

In this specific case, I ask you to address the remaining points of the referees in a detailed p-b-p-response and by text changes, clarifications and data re-analysis or different data presentation. As discussed, we would not require the addition of further major experiments, but if you already have data that would strengthen the manuscript in line with the referee reports, I ask you to add these.

When submitting your revised manuscript, please also carefully review the instructions that follow below.

PLEASE NOTE THAT upon resubmission revised manuscripts are subjected to an initial quality control prior to exposition to re-review. Upon failure in the initial quality control, the manuscripts are sent back to the authors, which may lead to delays. Frequent reasons for such a failure are the lack of the data availability section (please see below) and the presence of statistics based on n=2 (the authors are then asked to present scatter plots or provide more data points).

When submitting your revised manuscript, we will require:

1) a .docx formatted version of the final manuscript text (including legends for main figures, EV figures and tables), but without the figures included. Please make sure that changes are highlighted to be clearly visible. Figure legends should be compiled at the end of the manuscript text.

2) individual production quality figure files as .eps, .tif, .jpg (one file per figure), of main figures and EV figures. Please upload these as separate, individual files upon re-submission. Please make sure that all figure panels are called out separately and sequentially in the manuscript text

The Expanded View format, which will be displayed in the main HTML of the paper in a collapsible format, has replaced the Supplementary information. You can submit up to 5 images as Expanded View. Please follow the nomenclature Figure EV1, Figure EV2 etc. The figure legend for these should be included in the main manuscript document file in a section called Expanded View Figure Legends after the main Figure Legends section. Additional Supplementary material should be supplied as a single pdf file labeled Appendix. The Appendix should have page numbers and needs to include a table of content on the first page (with page numbers) and legends for all content. Please follow the nomenclature Appendix Figure Sx, Appendix Table Sx etc. throughout the text, and also label the figures and tables according to this nomenclature.

I would suggest having 7 main figures and to combine the present supplementary figures into 5 EV figures. Then, we would not need an Appendix.

For more details please refer to our guide to authors:
http://www.embopress.org/page/journal/14693178/authorguide#manuscriptpreparation

See also our guide for figure preparation:
http://wol-prod-cdn.literatumonline.com/pb-assets/embo-site/EMBOPress_Figure_Guidelines_061115-1561436025777.pdf

See also the guidelines for figure legend preparation:
https://www.embopress.org/page/journal/14693178/authorguide#figureformat

3) a .docx formatted letter INCLUDING the reviewers' reports and your detailed point-by-point responses to their comments. As

part of the EMBO Press transparent editorial process, the point-by-point response is part of the Review Process File (RPF), which will be published alongside your paper.

4) a complete author checklist, which you can download from our author guidelines (https://www.embopress.org/page/journal/14693178/authorguide). Please insert page numbers in the checklist to indicate where the requested information can be found in the manuscript. The completed author checklist will also be part of the RPF.

Please also follow our guidelines for the use of living organisms, and the respective reporting guidelines: http://www.embopress.org/page/journal/14693178/authorguide#livingorganisms

5) that primary datasets produced in this study (e.g. RNA-seq, ChIP-seq and array data) are deposited in an appropriate public database. This is now mandatory (like the COI statement). If no primary datasets have been deposited in any database, please state this in this section (e.g. 'No primary datasets have been generated and deposited').

See also: http://embor.embopress.org/authorguide#datadeposition

Please remember to provide a reviewer password if the datasets are not yet public.

The accession numbers and database should be listed in a formal "Data Availability " section (placed after Materials & Methods) that follows the model below. Please note that the Data Availability Section is restricted to new primary data that are part of this study.

Data availability

The datasets produced in this study are available in the following databases:

- RNA-Seq data: Gene Expression Omnibus GSE46843 (https://www.ncbi.nlm.nih.gov/geo/query/acc.cgi?acc=GSE46843)
- [data type]: [name of the resource] [accession number/identifier/doi] ([URL or identifiers.org/DATABASE:ACCESSION])

*** Note - All links should resolve to a page where the data can be accessed. ***

Moreover, I have these editorial requests:

6) We strongly encourage the publication of original source data with the aim of making primary data more accessible and transparent to the reader. The source data will be published in a separate source data file online along with the accepted manuscript and will be linked to the relevant figure. If you would like to use this opportunity, please submit the source data (for example scans of entire gels or blots, data points of graphs in an excel sheet, additional images, etc.) of your key experiments together with the revised manuscript. If you want to provide source data, please include size markers for scans of entire gels, label the scans with figure and panel number, and send one PDF file per figure.

7) Our journal encourages inclusion of *data citations in the reference list* to directly cite datasets that were re-used and obtained from public databases. Data citations in the article text are distinct from normal bibliographical citations and should directly link to the database records from which the data can be accessed. In the main text, data citations are formatted as follows: "Data ref: Smith et al, 2001" or "Data ref: NCBI Sequence Read Archive PRJNA342805, 2017". In the Reference list, data citations must be labeled with "[DATASET]". A data reference must provide the database name, accession number/identifiers and a resolvable link to the landing page from which the data can be accessed at the end of the reference. Further instructions are available at: http://www.embopress.org/page/journal/14693178/authorguide#referencesformat

8) Regarding data quantification and statistics, please make sure that the number "n" for how many independent experiments were performed, their nature (biological versus technical replicates), the bars and error bars (e.g. SEM, SD) and the test used to calculate p-values is indicated in the respective figure legends (also for potential EV figures and all those in the final Appendix). Please also check that all the p-values are explained in the legend, and that these fit to those shown in the figure. Please provide statistical testing where applicable. Please avoid the phrase 'independent experiment', but clearly state if these were biological or technical replicates. See also: http://www.embopress.org/page/journal/14693178/authorguide#statisticalanalysis

9) Please note our reference format: http://www.embopress.org/page/journal/14693178/authorguide#referencesformat

10) We updated our journal's competing interests policy in January 2022 and request authors to consider both actual and perceived competing interests. Please review the policy https://www.embopress.org/competing-interests and add a statement declaring your competing interests. Please name that section 'Disclosure and Competing Interests Statement' and add it after the author contributions section. Please remove the COI statement from the title page.

11) We now use CRediT to specify the contributions of each author in the journal submission system. CRediT replaces the author contribution section. Please use the free text box to provide more detailed descriptions. Thus, please remove the author contributions section from the manuscript text file. See also guide to authors: https://www.embopress.org/page/journal/14693178/authorguide#authorshipguidelines

12) Please order the manuscript sections like this using these names:
Title page - Abstract - Introduction - Results - Discussion - Materials and Methods - Data availability section (DAS) - Acknowledgements - Disclosure and Competing Interests Statement - References - Figure legends - Expanded View Figure legends

13) Please move all the material and methods information to the main text.

14) Please name 'Table S1' 'Table EV1' and uploaded in word or excel format.

15) Please also make sure that all the funding information is also entered into the online submission system and is complete and similar to the one in the manuscript text file (acknowledgements).

In addition, I would need from you:
- a short, two-sentence summary of the manuscript (around 35 words).
- three to four short bullet points highlighting the key findings of your study (2 lines each).
- a schematic summary figure (in jpeg or tiff format with the exact width of 550 pixels and a height of not more than 400 pixels) that can be used as a visual synopsis on our website.

I look forward to seeing a revised version of your manuscript when it is ready. Please let me know if you have questions or comments regarding the revision.

Please use this link to submit your revision: https://embor.msubmit.net/cgi-bin/main.plex

Kind regards,

Achim

Achim Breiling
Senior Editor
EMBO Reports

Referee #2:

As already mentioned in the review of the original manuscript, the paper was very hard to read and follow. I found that the revised manuscript is not improved in this aspect. Even to someone that is well-versed in the molecules and pathways discussed in the paper, it was impossible to follow the logic of the experiments and often there was the feeling that a leap from one topic to the other left a large unfilled gap of knowledge unaddressed. This is largely due to the fact that the experimental systems change constantly even within a single figure, but this is further amplified by the fact that the different areas covered are not adequately connected. A critical point is also that the authors ignore or downplay discrepancies in the data that have important implications on the interpretation of the experiments. I acknowledge the amount of work that has been performed, however, in my view this manuscript as it is written and presented will cause more confusion than clarity even to specialists. For non-specialists, reading the paper will be a daunting task and I don't think that they will go further than the abstract. For these reasons, I do not recommend publication of this work in the EMBO Journal.

I include some specific comments below that illustrate some of the problems, without going through a very detailed list of all experiments with similar issues.

In the new experiments using clone B9 (Fig. 1K), the induction of CXCL8 is expression is 10x less compared to the pool (100 vs 1000 ng/ml). I would have expected a much more robust cytokine response, while it seems that the increase in CXCL8 expression is very low and not even statistically significant after 50ng/ml DOX. How can this be explained considering that this clone shows expression of ZBP1 in all cells compared to about one third in the pool?

The entire ubiquitination part is very confusing. For example, the authors claim that in the absence of RIPK1 ZBP1 in not ubiquitinated. However, unmodified ZBP1 comes down in the TUBE pulldown in Fig 4D. How is this possible? It is striking this is not discussed by the authors considering their expertise in the ubiquitin field. Similarly, how do all Ub ligases and DUBs co-IP with ZBP1 in the absence of RIPK1 (Fig 5F)? There are several such observations that are left unexplained and are largely ignored in the paper.

In the last part of the paper discussing the amplification of cytokine production by caspase-8 inhibition, the authors conclude that RIPK3 kinase activity and MLKL mediate this response independently of cell death but do not discuss how MLKL can amplify cytokine production. After all, activated MLKL damages the plasma membrane, how does this lead to cytokine production?

The comparison of the ZBP1-mediated signalling and RIPK3 dimerisation-induced signalling is done largely using different cell lines and is therefore not possible to correlate the findings. For example, in Figures 4 E,F,G and Figure 5 A,G,H, it is not clear why use the HEK293FT/RIPK3-2xFV cells instead of HT29 cells to directly compare with the ZBP1 part? This creates a large gap as in HEK293FT/RIPK3-2xFV cells there is a need to show cytokines production particular TNF to rule out an autocrine TNF effect in these cells, and also ZBP1 and RIPK1 ubiquitination assays to show these ubiquitination processes happened in HEK293FT/RIPK3-2xFV cells (no cell death data showed in these cells upon ZBP1 induction accompanying Figure 2H). The jump from one cell line to the other makes it impossible to compare the results.

In the neutrophil migration assays in Fig 1L-N, why is supernatant from cells stimulated with 500 ng/ml DOX used instead of 50 ng/ml? How much of the effect seen is due to cell death? Also, why is zVAD combined with Nec1s in panel N and not with RIPK3 inhibitor?

There are also several details that show discrepancies between experiments that are not discussed. For example, in Fig. 1D, ZBP1mZa1a2 expression induced by 500 ng DOX does not cause cell death over 72 hours. However, in Fig. S1A, the same amount of DOX in the same cells causes significantly increased cell death at 48 hours.

Finally, the SARS-CoV-2 part is entirely disconnected from the rest of the paper. It does seem as if the authors try to use the timeliness of the virus causing COVID to boost the chances of their paper getting published, which might be understandable but does not improve the quality of the manuscript.

Referee #3:

The revised manuscript is much improved. I am satisfied with their experiments ruling out the contribution of cell death in the culture.

However, many of my previous concerns were unfortunately not satisfactorily addressed. For example, we are still not closer to understand why their over-expression system behaves so different to endogenous ZBP1 in mice (raised in point 1). It is important to resolve whether the difference in signalling is a ZBP1 over-expression effect, or actually reflects a true difference between human versus mouse. This could easily have been addressed by comparing the behaviour of the same over-expression system in mouse cells. But unfortunately, no such comparative experiments were conducted.

Likewise, the authors decided not to corroborate their data using endogenous ZBP1 (Response to point 2). I feel, this is a missed opportunity. While their insights are valuable, one is left wondering whether ZBP1 is capable of signalling in the same manner at physiological levels.

My concern raised in point 3 was also not addressed. No alternative, necroptosis proficient human cell line was used. Hence, the signalling data (NF-kB as well as apoptosis and necroptosis signalling) relies on HT29 cells only.

All in all, this manuscript provides some interesting leads but certain aspects could have been strengthened to corroborate their findings.

Point-by-point Response – Peng et al. EMBO Reports

Referee #2:

As already mentioned in the review of the original manuscript, the paper was very hard to read and follow. I found that the revised manuscript is not improved in this aspect. Even to someone that is well-versed in the molecules and pathways discussed in the paper, it was impossible to follow the logic of the experiments and often there was the feeling that a leap from one topic to the other left a large unfilled gap of knowledge unaddressed. This is largely due to the fact that the experimental systems change constantly even within a single figure, but this is further amplified by the fact that the different areas covered are not adequately connected. A critical point is also that the authors ignore or downplay discrepancies in the data that have important implications on the interpretation of the experiments. I acknowledge the amount of work that has been performed, however, in my view this manuscript as it is written and presented will cause more confusion than clarity even to specialists. For non-specialists, reading the paper will be a daunting task and I don't think that they will go further than the abstract. For these reasons, I do not recommend publication of this work in the EMBO Journal.

We are surprised that the reviewer does not find the manuscript improved and that they have not commented on the new data we included relating to ZBP1-induced cell death vs. cytokine production, which was one of the main points raised in the original review report.

As outlined in our point-to-point response and the cover letter, we have followed the advice of the reviewers to separate the model systems more clearly. However, we acknowledge that the study is quite data-heavy as we employ several systems to investigate inflammatory signaling vs. cell death by ZBP1 and RIPK3. In addition, we investigate the RIPK3-mediated inflammatory signaling following TSZ treatment. Based on the reviewer's comments and those of other reviewers, we have re-assessed the data included in the manuscript and acknowledge that data relating to the enhanced inflammatory signaling when Casp8 activity is inhibited may be confusing and distracts from the main focus of the study (Figure 6). In particular, the data shown for TSZ-induced inflammatory signaling may be considered superfluous as it essentially describes the same phenomenon previously shown by Zhu et al. CCDis 2018. We have therefore decided to remove the data shown in Figure 6D-6H and Figure S6A from the revised manuscript.

We have also made significant changes to the organization of the manuscript to completely separate the analysis of ZBP1-induced signaling and the analysis of signaling induced by the oligomerization of RIPK3-2xFV. The resulting revised manuscript now contains 6 Main Figures and 5 Extra-view figures. The complete list of the figure changes is at the end of document.

I include some specific comments below that illustrate some of the problems, without going through a very detailed list of all experiments with similar issues.

In the new experiments using clone B9 (Fig. 1K), the induction of CXCL8 is expression is 10x less compared to the pool (100 vs 1000 ng/ml). I would have expected a much more robust cytokine response, while it seems that the increase in CXCL8 expression is very low and not even statistically significant after 50ng/ml DOX. How can this be explained considering that this clone shows expression of ZBP1 in all cells compared to about one third in the pool?

We apologize for the confusion. The new experiments were performed using a 4-fold lower density of cells to accommodate accurate measurements of cell death. At higher cell densities, the SytoxGreen exclusion assay resulted in a high percentage of false-positive cells at baseline. This resulted in 8-10 fold lower cytokine concentrations in the media both at baseline and after Dox-treatment. It is also possible that the measurement of the absolute cytokine concentration is influenced by the fact that the two sets of experiments were performed several years apart in two different laboratories and by different researchers. We have performed new ELISA measurement of cytokine production by the pooled HT29 /TO-ZBP1 cells using the same cell densitiy and culturing conditions used for data shown in Figure 1and the cytokine production of these is comparable to those measured using the B9 single-cell clone (See figure below). Why the single-cell clone population does not produce 3x more cytokines than the pooled population we don't know but there well may well be cell to cell variation in the capacity to produce and secrete cytokines. Nonetheless, we do not believe this puts into question the validity of the data in any way as all experimental conditions are internally compared within the cell line. Also, the aim of the experiments was to investigate how cell death would affect cytokine production and not to compare the absolute amount of cytokines produced by different cell lines.

We have included a note about the culturing conditions in the revised manuscript (Page 7, bottom).

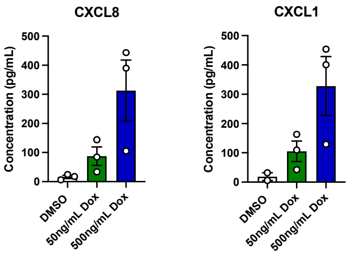

Cytokine measurements in media from HT29 / TO-ZBP1 cells stimulated with Dox for 24 h and cultured under the same conditions as used for the experiments with the monoclonal B9 HT29 / TO-ZBP1 cells shown in Figure 1J, and EV1G.

The entire ubiquitination part is very confusing. For example, the authors claim that in the absence of RIPK1 ZBP1 in not ubiquitinated. However, unmodified ZBP1 comes down in the TUBE pulldown in Fig 4D. How is this possible? It is striking this is not discussed by the authors considering their expertise in the ubiquitin field. Similarly, how do all Ub ligases and DUBs co-IP with ZBP1 in the absence of RIPK1 (Fig 5F)? There are several such observations that are left unexplained and are largely ignored in the paper.

Enrichment of Ub-conjugates using TUBEs, 1xUBA and SUBs result in Ub-independent co-purification of some, but not all, proteins. We have observed this for RIPK1 and other proteins in previous studies (Fiil et al. Mol Cell 2013, Hrdinka et al. Cell Reports 2016) and have not been able to find conditions where this can be avoided. The enrichment of Ub-conjugates happens under non-denaturing conditions and therefore Ub-independent protein-protein interactions are also maintained, which also maintains interactions between modified and non-modified forms of proteins that are dimeric/oligomeric or are part of the complex. This is an inherent limitation of methodology.

We have included a brief explanation of this in the revised manuscript (Page 11, mid/bottom)

In the last part of the paper discussing the amplification of cytokine production by caspase-8 inhibition, the authors conclude that RIPK3 kinase activity and MLKL mediate this response independently of cell death but do not discuss how MLKL can amplify cytokine production. After all, activated MLKL damages the plasma membrane, how does this lead to cytokine production?

We have indeed not addressed HOW MLKL contributes to cytokine production, and we agree it is unexpected that MLKL can have this function. However, Zhu et al. CDDis 2018 showed a similar phenomenon following treatment of HT29 cells with TSZ. Based on the comments by the reviewer, we have decided to remove the relevant figure panels in the revised manuscript (See details in our responses to the reviewer’s general comment)

The comparison of the ZBP1-mediated signalling and RIPK3 dimerisation-induced signalling is done largely using different cell lines and is therefore not possible to correlate the findings. For example, in Figures 4 E,F,G and Figure 5 A,G,H, it is not clear why use the HEK293FT/RIPK3-2xFV cells instead of HT29 cells to directly compare with the ZBP1 part? This creates a large gap as in HEK293FT/RIPK3-2xFV cells there is a need to show cytokines production particular TNF to rule out an autocrine TNF effect in these cells, and also ZBP1 and RIPK1 ubiquitination assays to show these ubiquitination processes happened in HEK293FT/RIPK3-2xFV cells (no cell death data showed in these cells upon ZBP1 induction accompanying Figure 2H). The jump from one cell line to the other makes it impossible to compare the results.
HEK293T cells were used as a separate cell model to study ZBP1-induced inflammatory signaling and the role of the Ub-machinery (LUBAC, cIAPs, OTULIN, CYLD, K63/M1-Ub chains) in facilitating signaling. In Figure 2G-2H, we show that ZBP1-induced NF-kappaB activity in HEK293T cells is mediated by the Za domains in ZBP1 and by RIPK3, and that NF-kappaB activation is not inhibited by RIPK1 or RIPK3 kinase inhibitors (or by the MLKL inhibitor NSA). Thus, the ZBP1-induced pathway in HEK293T cells appears to be quite similar as that induced in the HT29 cell system. In Figure 3 and 4 of the revised manuscript, the role of ubiquitin chains in ZBP1 signaling is investigated biochemically and functionally using HEK293T and HT29 cells. We believe the new organization of the figures makes it easier to follow which experiments are performed in what cell systems.

In the neutrophil migration assays in Fig 1L-N, why is supernatant from cells stimulated with 500 ng/ml DOX used instead of 50 ng/ml? How much of the effect seen is due to cell death? Also, why is zVAD combined with Nec1s in panel N and not with RIPK3 inhibitor?
500 ng/mL Dox was used because it induced a more robust increase in chemotactic cytokines in the media than did 50 ng/mL. The conditioned media from cells treated with 500 ng/mL Dox increased the migration of primary neutrophils by no more than 20%-30%, making it unlikely that 50 ng/mL Dox would induce sufficient cytokine production for this assay.

We mention this in the revised manuscript (Page 8, bottom / Page 9, top).

To investigate if cell death under these conditions contributed to the neutrophil migration, we treated HT29 / TO-ZBP1 cells with Nec1s + zVAD-fmk since the combination of these two inhibitors restored viability of the cells following treatment with 500 ng/mL Dox (Figure 1F). We could also have used zVAD-fmk + a RIPK3 inhibitor, but we unfortunately did not include this combination in the experiments.

Since Nec1s+zVAD treatment did not inhibit neutrophil migration, we are confident about our interpretation of the data that the increased migration of neutrophils is a result of ZBP1-induced inflammatory signaling and not of cell death. This interpretation is also supported by the observation that treatment of HT29 / TO-ZBP1 cells with IKK and TAK1 inhibitors in combination with Dox completely abolished the increase in neutrophil migration (Figure 2C).

There are also several details that show discrepancies between experiments that are not discussed. For example, in Fig. 1D, ZBP1mZa1a2 expression induced by 500 ng DOX does not cause cell death over 72 hours. However, in Fig. S1A, the same amount of DOX in the same cells causes significantly increased cell death at 48 hours.
The perceived discrepancy raised by the reviewer appears to arise from our use to two types of assays to determine cell death; direct determination of dead cells and loss of viability. Cell death measurements were based on loss of cell membrane integrity detected by uptake of SytoxGreen. Viability was determined using the CellTitre-Glo assay, which measures ATP levels in the culture as a measure of metabolically active cells. Viability measurements measure the net loss/accumulation of metabolically active cells as a result of cell death and proliferation. We employed these two measurements to detect both intact dead cells (SytoxGreen) and the net loss of cells from the culture, irrespective of whether they remained intact or had disintegrated. Since cell proliferation is incorporated into the viability measurement, a loss of viability might not be detected when a relatively small percentage of cells die and this happens at later time points (e.g. after 24 h) as increased proliferation may compensate for the loss of cells.

Finally, the SARS-CoV-2 part is entirely disconnected from the rest of the paper. It does seem as if the authors try to use the timeliness of the virus causing COVID to boost the chances of their paper getting published, which might be understandable but does not improve the quality of the manuscript.

The rationale for addressing the role of ZBP1 in the response to SARS-CoV-2 infection was our analysis of available datasets from published studies of cells/tissues from patients with COVID-19. The analysis showed that ZBP1 expression is strongly induced by SARS-CoV-2 infection and correlated with the severity of disease (Figure EV5A-C). We respectfully disagree with the reviewer that the data is disconnected. Whilst the data does not address if ZBP1-dependent signaling under these circumstances involves cell death, it provides evidence that endogenous ZBP1 contributes to production of cytokines in response to virus infection in Calu-3 cells. This is within the main focus of the rest of the study, and we feel it is relevant to the research community to have this information.

Referee #3:

The revised manuscript is much improved. I am satisfied with their experiments ruling out the contribution of cell death in the culture.

We appreciate the reviewer's acknowledgement that we have amply addressed one of the main concerns raised by all reviewers of the original manuscript. We indeed focused our efforts during the revision to address whether low-level cell death might be responsible for the inflammatory response after ZBP1 expression. Demonstrating that this is NOT the case is, in our view, central to the study because it shows that ZBP1 can form a signaling platform that activates signaling akin to other immune receptors.

However, many of my previous concerns were unfortunately not satisfactorily addressed. For example, we are still not closer to understand why their over-expression system behaves so different to endogenous ZBP1 in mice (raised in point 1). It is important to resolve whether the difference in signalling is a ZBP1 over-expression effect, or actually reflects a true difference between human versus mouse. This could easily have been addressed by comparing the behaviour of the same over-expression system in mouse cells. But unfortunately, no such comparative experiments were conducted.
We agree with the reviewer that it will be important to understand the basis for the different ZBP1-mediated responses in human vs. mouse and under different conditions. However, this is unlikely to be a simple task as illustrated by accumulating evidence from murine systems that the signaling outcomes of ZBP1 engagement is context-specific; for example, during the revision of the manuscript, several studies were published that show that ZBP1 mediates fatal autoinflammation and immune pathology in mice caused by mutations in the RNA deaminase ADAR1, including two studies by co-authors of this manuscript. Whilst ZBP1 was found to contribute to cell death, inhibition of caspase-8-mediated apoptosis and RIPK3-MLKL-mediated necroptosis did not rescue the pathology and death of the animals caused by ADAR1 mutation. Instead, ZBP1 was proposed to mediate pathogenic type-I IFN responses. This contrasts the published role of ZBP1 in mediating cell death-driven skin inflammation in mice when RIPK1 is absent and of ZBP1 in the host-defense to viral infection in mice. This highlights that there are fundamental differences in the mechanism by which ZBP1 contributes to inflammation within mouse models, depending on the model. We therefore believe that this topic should be addressed in depth in a stand-alone future study/studies.

Regarding a direct comparison of the response to over-expression of human vs. mouse ZBP1. One of the studies published by one of the co-authors of this study (Andrew Oberst, Hubbard et al. Nature 2022) showed that chemical oligomerization of 2xFV-ZBP1 in mouse embryonic fibroblasts stimulated cytokine production via RIPK1 and independently of cell death, suggesting that ZBP1 in that context stimulates inflammatory signaling via molecular mechanisms that are similar to those we show in human cells.

Likewise, the authors decided not to corroborate their data using endogenous ZBP1 (Response to point 2). I feel, this is a missed opportunity. While their insights are valuable, one is left wondering whether ZBP1 is capable of signalling in the same manner at physiological levels.

We agree that it would be relevant to investigate the ZBP1 signaling pathway without overexpression/ectopic expression of components. However, this is not as trivial as it may seem given the complexity of establishing conditions where both ZBP1 protein and/or ZBP1 ligands accumulate acutely in the cell, which is key for biochemical investigation of signaling pathways. We are planning to establish such systems using inducible ADAR1 knockdown and/or systems to degrade ADAR1 but this requires substantial optimization and validation of the system, since complete absence of ADAR1 leads to cell death and ZBP1 expression would need to be induced by type-I IFN treatment. We would also point to the data presented in three back-to-back studies (Hubbard et al., Jiao et al., de Reuver et al. Nature 2022) which provide compelling evidence that ZBP1 mediates signaling and immune pathology independently of apoptosis/necroptosis. Notably, the study by Hubbard et al. further reported that ablation of *Casp8* exacerbated the mouse pathology and this is associated with upregulation of several pro-inflammatory NF-kappaB target genes. This is consistent with our data showing that zVAD enhances cytokine production in response to ZBP1 expression. Irrespective of these findings, it will be important to investigate the molecular details and regulation of the endogenous ZBP1 pathway in the future.

My concern raised in point 3 was also not addressed. No alternative, necroptosis proficient human cell line was used. Hence, the signalling data (NF-kB as well as apoptosis and necroptosis signalling) relies on HT29 cells only.

We indeed attempted to address this using the human keratinocyte cell line HaCaT cells, which previously has been shown to undergo necroptosis and upregulate pro-inflammatory cytokines in response to TSZ treatment (Duan et al. CCDis 2020). However, the Dox-inducible ZBP1 expressing cells we generated constitutively secreted high levels of cytokines, precluding an assessment of ZBP1 inflammatory signaling vs. cell death responses.

However, the molecular pathway activated by ZBP1 expression to stimulate NF-kappaB activity was investigated also in HEK293T cells with stable RIPK3 expression. Like the HT29 /TO-ZBP1 cell model, inflammatory signaling in HEK293T cells relied on intact ZBP1 Zalpha domains and was mediated by RIPK3 but not its kinase activity, and ZBP1 signaling was blocked by IAP antagonists (CpA) in both cell systems. Our study does not exclude that there might also be cell-type differences but based on studies of other immune receptor signaling, e.g. the TNFR1 and NOD2 pathway, the central components of the signaling pathway tend to be the same irrespective of the cell system in which they are studied. One of the main scopes of the study was to characterize the components needed for inflammatory signaling in the context of ZBP1, which we feel we have done.

All in all, this manuscript provides some interesting leads but certain aspects could have been strengthened to corroborate their findings.

List of figure changes:

Figure 1H -> incorporated into EV1C. The rest of Figure 1 panels are re-numbered accordingly.

Figure 2: Unchanged

Figure 3A -> Figure 5A
Figure 3B-C -> EV4D-E
Figure 3D -> EV4C
Figure 3E -> EV4F
Figure 3F-G -> EV4A-B
Figure 3H-N -> Figure 5B-G

Figure 4 -> Figure 3
Anti-P-RIPK3 blots in Figure 4B (Figure 3B in revised manuscript) have been exchanged with correct blots. In the original figure, the blots were anti-RIPK3 blots that had been copied in by mistake. The uncropped blots are shown in the Source Data Figure.

Figure 5A-F -> Figure 4A-F
Figure 5G -> Figure 5K
Figure 5H -> Figure 5J
Figure 5I -> Figure 5H

Figure 6A -> EV1H.
The column graph is reordered so +/- zVAD conditions are grouped. The western blots are changed to blots where samples were loaded in the corresponding order. The reason to change the order is to more clearly show the effects of the necroptosis inhibitors (GSK872, Nec1s, NSA) on cytokine production in the presence and absence of zVAD.

Figure 6B -> Figure 5L.
Only *CXCL8* expression is shown now in order to simplify the data presentation and to align with expression data in Figure 5M.

Figure 6C -> Figure 5M

Figure 6D-H -> Removed.
Based on reviewer comments, our assessment is that the data shown in these panels do not add insights but rather cause confusion since the observations are not followed up with additional experiments to elucidate the mechanism. The data shown in Figure 6E-H is essentially the same phenomenon as was reported in Zhu et al. CCDis 2018

Figure 7A-B -> EV5A-B.

Figure 7C-H -> Figure 6A-F
Figure 7H (revised Figure 6F) has been modified to reflect the revised discussion of the RIPK1/RIPK3 kinase-dependent mechanism of cytokine production. In the revised manuscript, we now discuss the possibility that the enhancement of cytokine production in the presence of zVAD (caspase-8 inhibition) might be associated with necroptosis-associated inflammatory signaling.

Figure S1F -> EV1A. The rest of Figure EV1 panels are re-numbered accordingly.

Figure S2E-G -> EV3A-C

Figure S3A -> Removed.
The data is redundant. Gene expression of *CXCL8/1* in response to dimerizer treatment is also shown in Figure 5C. Expression of *CXCL1* and *A20* does not provide additional insights and was not determined in other experiments.

Figure S3B-D -> EV4G-I

Figure S4 -> EV3D

Figure S5A -> Figure 5I

Figure S5B-D -> EV4J-L

Figure S6A -> Removed.
Expression of *CXCL8* is shown in Figure 5L. *CXCL2*/3 expression does not provide additional insights and was not determined in other experiments.

Figure S7 -> EV6

1st Revision - Editorial Decision 30th Sep 2022

Dear Prof. Gyrd-Hansen,

Thank you for the submission of your revised manuscript to our editorial offices. I have now received the report from the referee (referee #2 from the submission to The EMBO Journal) that I asked to re-evaluate your study, you will find below. As you will see, the referee indicates that the study still has limitations, but now supports the publication in EMBO reports.

Before proceeding with formal acceptance, I have these editorial requests I ask you to address in a final revised version of the manuscript:

- Please change all the callouts for the EV figures in the manuscript text file. Presently, these are called out as 'Figure Sx'. Please use the nomenclature 'Figure EVx'.

- Please add a formal 'Data Availability Section' (DAS) after the Methods section (the manuscript sections should be ordered like this: Title page - Abstract - Introduction - Results - Discussion - Materials and Methods - Data availability section - Acknowledgements - Disclosure and Competing Interests Statement - References - Figure legends - Expanded View Figure legends). This is now mandatory, like the 'Disclosure and Competing Interests Statement'. If no primary datasets have been deposited, please state this in this section (e.g. 'No primary datasets have been generated and deposited').

- Please remove the sections 'Lead Contact' (this is the corresponding author mentioned on the title page) and 'Materials Availability' (this can be moved to the DAS) from the Materials and Methods part.

- It seems you used a live SARS-CoV-2 strain in this study. Please add a paragraph to the methods section (titled 'Biosafety') providing details on where and how biosafety-relevant experiments were performed and that these were approved, and by whom (institution, government).

- We now use CRediT to specify the contributions of each author in the journal submission system. CRediT replaces the author contribution section. Please use the free text box to provide more detailed descriptions. Thus, please remove the author contributions section from the manuscript text file. See also guide to authors: https://www.embopress.org/page/journal/14693178/authorguide#authorshipguidelines

- Please make sure that the number "n" for how many independent experiments were performed, their nature (biological versus technical replicates), the bars and error bars (e.g. SEM, SD) and the test used to calculate p-values is indicated in the respective figure legends (main, EV and Appendix figures), and that statistical testing has been done where applicable. Please avoid phrases like 'independent experiment', but clearly state if these were biological or technical replicates. Please add complete statistical testing to all diagrams (main, EV and Appendix figures). Please also indicate (e.g. with n.s.) if testing was performed, but the differences are not significant. It seems, presently not all diagrams have statistical testing (see e.g. panels 1C and 1G). Please check.

- Thank you for providing the source data for some of the Western blots. Could you submit also the remainder (i.e. source data for the blots shown in Figs. 1, 2, 6 and EV 1,3 and 5). Please upload the source data for the Western blots as one pdf file per figure (main and EV figures) include size markers for the scans of the entire blots and make sure the complete SD is uploaded. Please also label the blots and indicate what part of the blot is shown in the final figure.

- Finally, please find attached a word file of the manuscript text (provided by our publisher) with changes we ask you to include in your final manuscript text, and some queries, we ask you to address. Please use this file for further modifications and provide your final manuscript file with track changes, in order that we can see any modifications done.

I look forward to seeing the final revised version of your manuscript when it is ready. Please let me know if you have questions regarding the revision.

Please use this link to submit your revision: https://embor.msubmit.net/cgi-bin/main.plex

Best,

Achim

Achim Breiling
Senior Editor
EMBO Reports

Referee #1:

In general, the ms is not significantly improved. Clearly, the issues outlined by reviewer 2 and 3 still remain. However, the new outline works much better. Also improved are the experimental settings because the work in HT29 and HECT293 cells are now more complementary. Before, it wasn't clear whether the two systems respond equally. All in all, I think this ms reports an interesting aspect of ZBP1 biology that is supported by a flurry of recent paper. However, it's impact is somewhat limited as too much emphasis is given to an over-expression that does seem to differ to ZBP1 at physiological levels. Therefore, future work will be required to resolve this discrepancy.

The authors have addressed all minor editorial requests.

Prof. Mads Gyrd-Hansen
University of Copenhagen
LEO Foundation Skin Immunology Research Center
Blegdamsvej 3B
Copenhagen 2200
Denmark

Dear Prof. Gyrd-Hansen,

I am very pleased to accept your manuscript for publication in the next available issue of EMBO reports. Thank you for your contribution to our journal.

At the end of this email I include important information about how to proceed. Please ensure that you take the time to read the information and complete and return the necessary forms to allow us to publish your manuscript as quickly as possible.

As part of the EMBO publication's Transparent Editorial Process, EMBO reports publishes online a Review Process File to accompany accepted manuscripts. As you are aware, this File will be published in conjunction with your paper and will include the referee reports, your point-by-point response and all pertinent correspondence relating to the manuscript.

If you do NOT want this File to be published, please inform the editorial office within 2 days, if you have not done so already, otherwise the File will be published by default [contact: emboreports@embo.org]. If you do opt out, the Review Process File link will point to the following statement: "No Review Process File is available with this article, as the authors have chosen not to make the review process public in this case." Please note that the author checklist will still be published even if you opt out of the transparent process.

Thank you again for your contribution to EMBO reports and congratulations on a successful publication. Please consider us again in the future for your most exciting work.

Yours sincerely,

Achim Breiling
Senior Editor
EMBO Reports

****

THINGS TO DO NOW:

Please note that you will be contacted by Wiley Author Services to complete licensing and payment information. The required 'Page Charges Authorization Form' is available here: https://www.embopress.org/pb-assets/embo-site/er_apc.pdf - please download and complete the form and return to embopressproduction@wiley.com

EMBO Press participates in many Publish and Read agreements that allow authors to publish Open Access with reduced/no publication charges. Check your eligibility: https://authorservices.wiley.com/author-resources/Journal-Authors/open-access/affiliation-policies-payments/index.html

You will receive proofs by e-mail approximately 2-3 weeks after all relevant files have been sent to our Production Office; you should return your corrections within 2 days of receiving the proofs.

Please inform us if there is likely to be any difficulty in reaching you at the above address at that time. Failure to meet our deadlines may result in a delay of publication, or publication without your corrections.

All further communications concerning your paper should quote reference number EMBOR-2022-55839V3 and be addressed to emboreports@wiley.com.

Should you be planning a Press Release on your article, please get in contact with emboreports@wiley.com as early as possible, in order to coordinate publication and release dates.

EMBO Press Author Checklist

Corresponding Author Name: Mads Gyrd-Hansen	
Journal Submitted to: EMBO Reports	
Manuscript Number: EMBOR-2022-55839	

USEFUL LINKS FOR COMPLETING THIS FORM
The EMBO Journal - Author Guidelines
EMBO Reports - Author Guidelines
Molecular Systems Biology - Author Guidelines
EMBO Molecular Medicine - Author Guidelines

Reporting Checklist for Life Science Articles (updated January 2022)
This checklist is adapted from Materials Design Analysis Reporting (MDAR) Checklist for Authors. MDAR establishes a minimum set of requirements in transparent reporting in the life sciences (see Statement of Task: 10.31222/osf.io/9sm4x). Please follow the journal's guidelines in preparing your manuscript.
Please note that a copy of this checklist will be published alongside your article.

Abridged guidelines for figures
1. Data
The data shown in figures should satisfy the following conditions:
- the data were obtained and processed according to the field's best practice and are presented to reflect the results of the experiments in an accurate and unbiased manner.
- ideally, figure panels should include only measurements that are directly comparable to each other and obtained with the same assay.
- plots include clearly labeled error bars for independent experiments and sample sizes. Unless justified, error bars should not be shown for technical replicates.
- if n<5, the individual data points from each experiment should be plotted. Any statistical test employed should be justified.
- Source Data should be included to report the data underlying figures according to the guidelines set out in the authorship guidelines on Data Presentation.

2. Captions
Each figure caption should contain the following information, for each panel where they are relevant:
- a specification of the experimental system investigated (eg cell line, species name).
- the assay(s) and method(s) used to carry out the reported observations and measurements.
- an explicit mention of the biological and chemical entity(ies) that are being measured.
- an explicit mention of the biological and chemical entity(ies) that are altered/varied/perturbed in a controlled manner.
- the exact sample size (n) for each experimental group/condition, given as a number, not a range;
- a description of the sample collection allowing the reader to understand whether the samples represent technical or biological replicates (including how many animals, litters, cultures, etc.).
- a statement of how many times the experiment shown was independently replicated in the laboratory.
- definitions of statistical methods and measures:
  - common tests, such as t-test (please specify whether paired vs. unpaired), simple χ2 tests, Wilcoxon and Mann-Whitney tests, can be unambiguously identified by name only, but more complex techniques should be described in the methods section;
  - are tests one-sided or two-sided?
  - are there adjustments for multiple comparisons?
  - exact statistical test results, e.g., P values = x but not P values < x;
  - definition of 'center values' as median or average;
  - definition of error bars as s.d. or s.e.m.

Please complete ALL of the questions below.
Select "Not Applicable" only when the requested information is not relevant for your study.

Materials

Newly Created Materials	Information included in the manuscript?	In which section is the information available? (Reagents and Tools Table, Materials and Methods, Figures, Data Availability Section)
New materials and reagents need to be available; do any restrictions apply?	Not Applicable	

Antibodies	Information included in the manuscript?	In which section is the information available? (Reagents and Tools Table, Materials and Methods, Figures, Data Availability Section)
For **antibodies** provide the following information:<br>- Commercial antibodies: RRID (if possible) or supplier name, catalogue number and or/clone number<br>- Non-commercial: RRID or citation	Yes	Materials and Methods

DNA and RNA sequences	Information included in the manuscript?	In which section is the information available? (Reagents and Tools Table, Materials and Methods, Figures, Data Availability Section)
**Short novel DNA or RNA including primers, probes**: provide the sequences.	Yes	Materials and Methods

Cell materials	Information included in the manuscript?	In which section is the information available? (Reagents and Tools Table, Materials and Methods, Figures, Data Availability Section)
**Cell lines:** Provide species information, strain. Provide accession number in repository **OR** supplier name, catalog number, clone number, and/**OR** RRID.	Yes	Materials and Methods
**Primary cultures:** Provide species, strain, sex of origin, genetic modification status.	Yes	Materials and Methods
Report if the cell lines were recently **authenticated** (e.g., by STR profiling) and tested for mycoplasma contamination.	Yes	Materials and Methods

Experimental animals	Information included in the manuscript?	In which section is the information available? (Reagents and Tools Table, Materials and Methods, Figures, Data Availability Section)
**Laboratory animals or Model organisms:** Provide species, strain, sex, age, genetic modification status. Provide accession number in repository **OR** supplier name, catalog number, clone number, **OR** RRID.	Not Applicable	
**Animal observed in or captured from the field:** Provide species, sex, and age where possible.	Not Applicable	
Please detail **housing and husbandry conditions**.	Not Applicable	

Plants and microbes	Information included in the manuscript?	In which section is the information available? (Reagents and Tools Table, Materials and Methods, Figures, Data Availability Section)
**Plants:** provide species and strain, ecotype and cultivar where relevant, unique accession number if available, and source (including location for collected wild specimens).	Not Applicable	
**Microbes:** provide species and strain, unique accession number if available, and source.	Not Applicable	

Human research participants	Information included in the manuscript?	In which section is the information available? (Reagents and Tools Table, Materials and Methods, Figures, Data Availability Section)
If collected and within the bounds of privacy constraints report on age, sex and gender or ethnicity for all study participants.	Not Applicable	

Core facilities	Information included in the manuscript?	In which section is the information available? (Reagents and Tools Table, Materials and Methods, Figures, Data Availability Section)
If your work benefited from core facilities, was their service mentioned in the acknowledgments section?	Not Applicable	

Design

**Study protocol**	**Information included in the manuscript?**	**In which section is the information available?** (Reagents and Tools Table, Materials and Methods, Figures, Data Availability Section)
If study protocol has been **pre-registered, provide DOI in the manuscript**. For clinical trials, provide the trial registration number **OR** cite DOI.	Not Applicable	
Report the **clinical trial registration number** (at ClinicalTrials.gov or equivalent), where applicable.	Not Applicable	

**Laboratory protocol**	**Information included in the manuscript?**	**In which section is the information available?** (Reagents and Tools Table, Materials and Methods, Figures, Data Availability Section)
Provide DOI OR other citation details if **external detailed step-by-step protocols** are available.	Not Applicable	

**Experimental study design and statistics**	**Information included in the manuscript?**	**In which section is the information available?** (Reagents and Tools Table, Materials and Methods, Figures, Data Availability Section)
Include a statement about **sample size** estimate even if no statistical methods were used.	Not Applicable	
Were any steps taken to minimize the effects of subjective bias when allocating animals/samples to treatment (e.g. **randomization procedure**)? If yes, have they been described?	Not Applicable	
Include a statement about **blinding** even if no blinding was done.	Not Applicable	
Describe **inclusion/exclusion criteria** if samples or animals were excluded from the analysis. Were the criteria pre-established?<br><br>If sample or data points were omitted from analysis, report if this was due to attrition or intentional exclusion and provide justification.	Yes	Materials and Methods
For every figure, are **statistical tests** justified as appropriate? Do the data meet the assumptions of the tests (e.g., normal distribution)? Describe any methods used to assess it. Is there an estimate of variation within each group of data? Is the variance similar between the groups that are being statistically compared?	Yes	Materials and Methods

**Sample definition and in-laboratory replication**	**Information included in the manuscript?**	**In which section is the information available?** (Reagents and Tools Table, Materials and Methods, Figures, Data Availability Section)
In the figure legends: state number of times the experiment was **replicated** in laboratory.	Yes	Figures
In the figure legends: define whether data describe **technical or biological replicates**.	Yes	Figures

Ethics

**Ethics**	**Information included in the manuscript?**	**In which section is the information available?** (Reagents and Tools Table, Materials and Methods, Figures, Data Availability Section)
Studies involving **human participants**: State details of **authority granting ethics approval** (IRB or equivalent committee(s), provide reference number for approval.	Yes	Materials and Methods
Studies involving **human participants**: Include a statement confirming that **informed consent** was obtained from all subjects and that the experiments conformed to the principles set out in the WMA Declaration of Helsinki and the Department of Health and Human Services Belmont Report.	Yes	Materials and Methods
Studies involving **human participants:** For publication of **patient photos**, include a statement confirming that consent to publish was obtained.	Not Applicable	
Studies involving experimental **animals**: State details of **authority granting ethics approva**l (IRB or equivalent committee(s), provide reference number for approval. Include a statement of compliance with ethical regulations.	Not Applicable	
Studies involving **specimen and field samples:** State if relevant **permits** obtained, provide details of authority approving study; if none were required, explain why.	Not Applicable	

**Dual Use Research of Concern (DURC)**	**Information included in the manuscript?**	**In which section is the information available?** (Reagents and Tools Table, Materials and Methods, Figures, Data Availability Section)
Could your study fall under dual use research restrictions? Please check biosecurity documents and list of **select agents and toxins** (CDC): https://www.selectagents.gov/sat/list.htm	Not Applicable	
If you used a select agent, is the security level of the lab appropriate and reported in the manuscript?	Not Applicable	
If a study is subject to dual use research of concern regulations, is the name of the **authority granting approval and reference number** for the regulatory approval provided in the manuscript?	Not Applicable	

Reporting

The MDAR framework recommends adoption of discipline-specific guidelines, established and endorsed through community initiatives. Journals have their own policy about requiring specific guidelines and recommendations to complement MDAR.

**Adherence to community standards**	**Information included in the manuscript?**	**In which section is the information available?** (Reagents and Tools Table, Materials and Methods, Figures, Data Availability Section)
State if relevant guidelines or checklists (e.g., **ICMJE, MIBBI, ARRIVE, PRISMA**) have been followed or provided.	Not Applicable	
For **tumor marker prognostic studies,** we recommend that you follow the **REMARK** reporting guidelines (see link list at top right). See author guidelines, under 'Reporting Guidelines'. Please confirm you have followed these guidelines.	Not Applicable	
For **phase II and III randomized controlled trials**, please refer to the **CONSORT** flow diagram (see link list at top right) and submit the CONSORT checklist (see link list at top right) with your submission. See author guidelines, under 'Reporting Guidelines'. Please confirm you have submitted this list.	Not Applicable	

Data Availability

**Data availability**	**Information included in the manuscript?**	**In which section is the information available?** (Reagents and Tools Table, Materials and Methods, Figures, Data Availability Section)
Have **primary datasets** been deposited according to the journal's guidelines (see 'Data Deposition' section) and the respective accession numbers provided in the Data Availability Section?	Not Applicable	
Were **human clinical and genomic datasets** deposited in a public access-controlled repository in accordance to ethical obligations to the patients and to the applicable consent agreement?	Not Applicable	
Are **computational models** that are central and integral to a study available without restrictions in a machine-readable form? Were the relevant accession numbers or links provided?	Not Applicable	
If publicly available data were reused, provide the respective **data citations in the reference list**.	Yes	Materials and Methods AND Reference list
