# Supplementary material for: Human ZBP1 induces cell death‐independent inflammatory signaling via RIPK3 and RIPK1
Source: EMBO Rep. 2022 Oct 21;23(12):e55839. doi: 10.15252/embr.202255839 (PMC9724671; doi:10.15252/embr.202255839)

Figure EV4

B

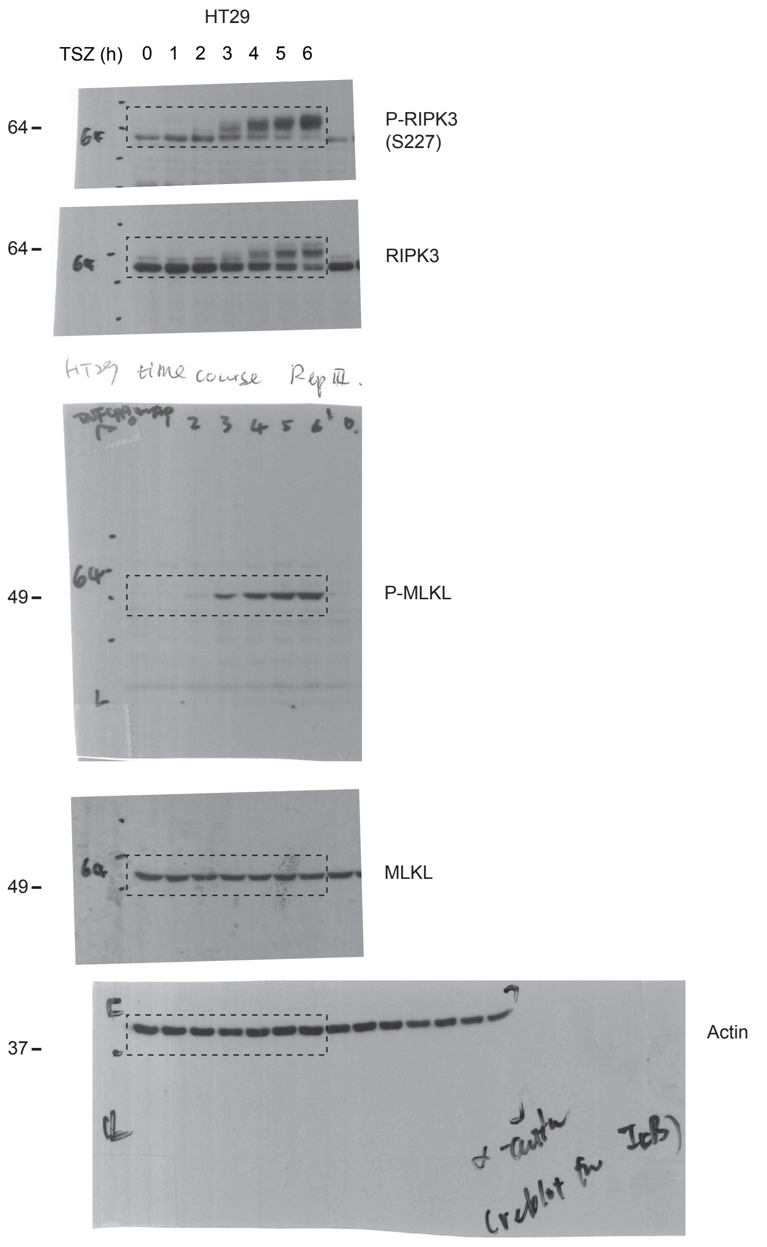

C

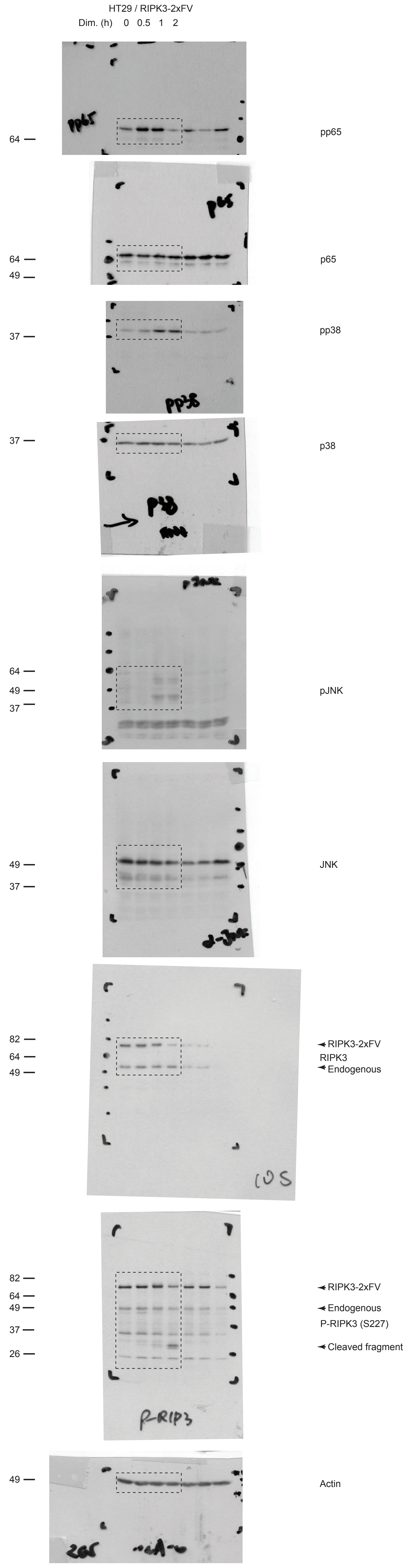

Supplement: Supplementary file 3 — Source Data for Expanded View [file EMBR-23-e55839-s011.zip › FigEV4.zip!/EMBOR-2022-55839V2-Figure_EV4_Source_Data-sd.pdf]
