# Supplementary material for: Human ZBP1 induces cell death‐independent inflammatory signaling via RIPK3 and RIPK1
Source: EMBO Rep. 2022 Oct 21;23(12):e55839. doi: 10.15252/embr.202255839 (PMC9724671; doi:10.15252/embr.202255839)
Supplement: Supplementary file 3 — Source Data for Expanded View [file EMBR-23-e55839-s011.zip › FigEV4.zip!/EMBOR-2022-55839V3-Figure_EV4_Source_Data-sd.pdf]

Figure EV4

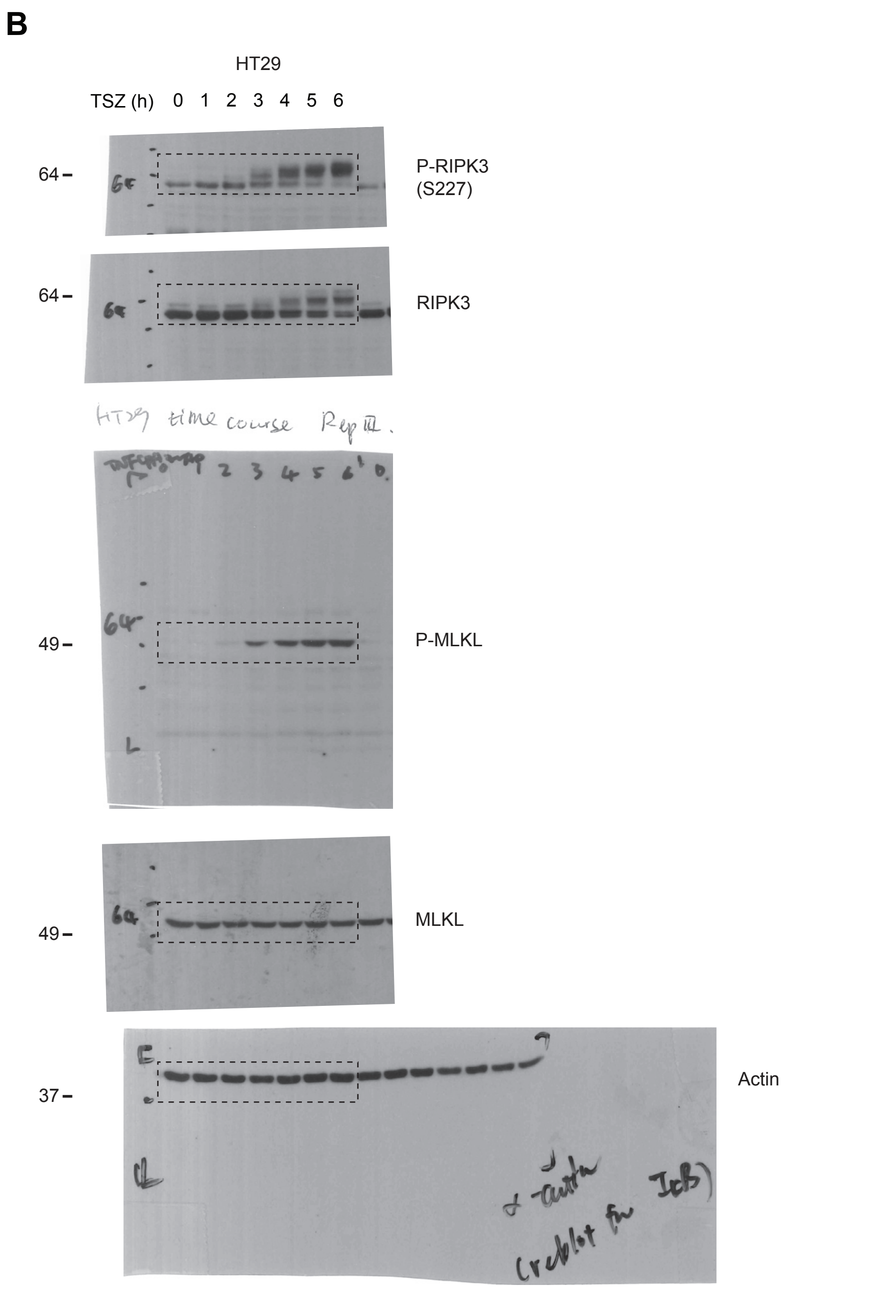
B
HT29
TSZ (h) 0 1 2 3 4 5 6
64
P-RIPK3 (S227)
64
RIPK3
49
P-MLKL
49
MLKL
37
Actin

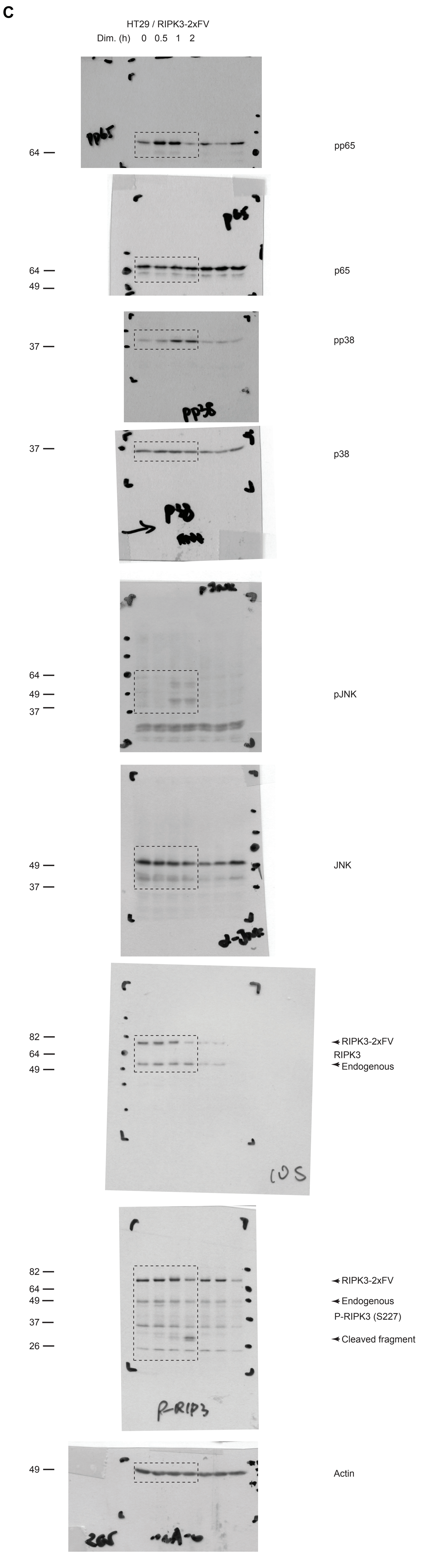
C
HT29 / RIPK3-2xFV
Dim. (h) 0 0.5 1 2
64
pp65
64
49
p65
37
pp38
37
p38
64
49
37
pJNK
49
37
JNK
82
64
49
RIPK3-2xFV
RIPK3
Endogenous
82
64
49
37
26
RIPK3-2xFV
Endogenous
P-RIPK3 (S227)
Cleaved fragment
49
Actin

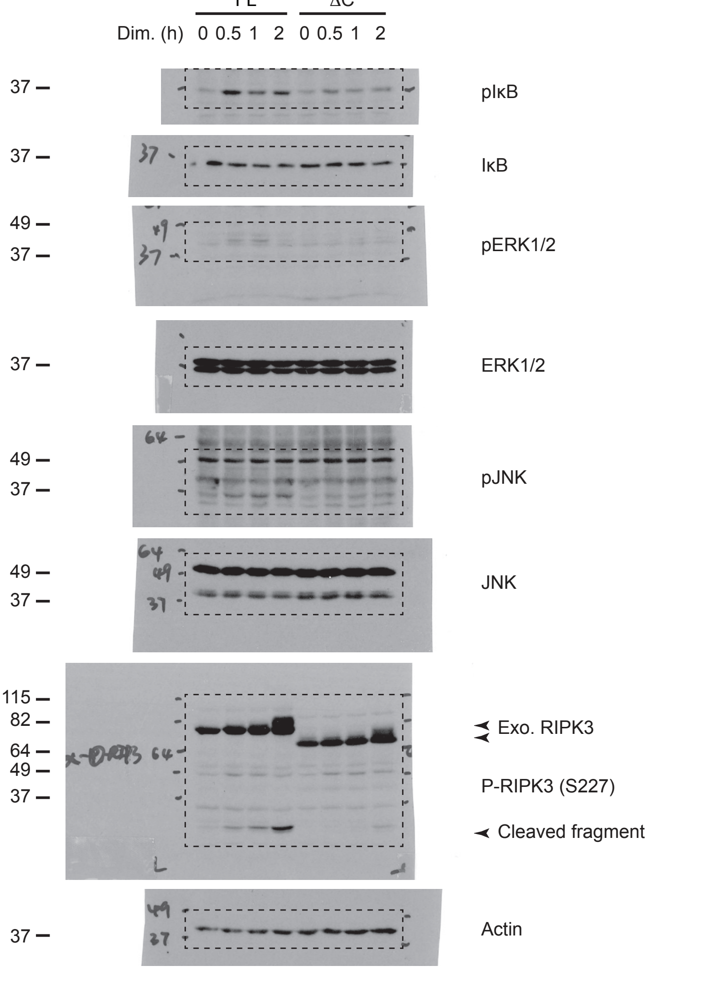
G
HEK293FT / RIPK3-2xFV
FL
ΔC
Dim. (h) 0 0.5 1 2 0 0.5 1 2
37
pIκB
37
IκB
49
37
pERK1/2
37
ERK1/2
49
37
pJNK
49
37
JNK
115
82
64
49
37
Exo. RIPK3
P-RIPK3 (S227)
Cleaved fragment
37
Actin

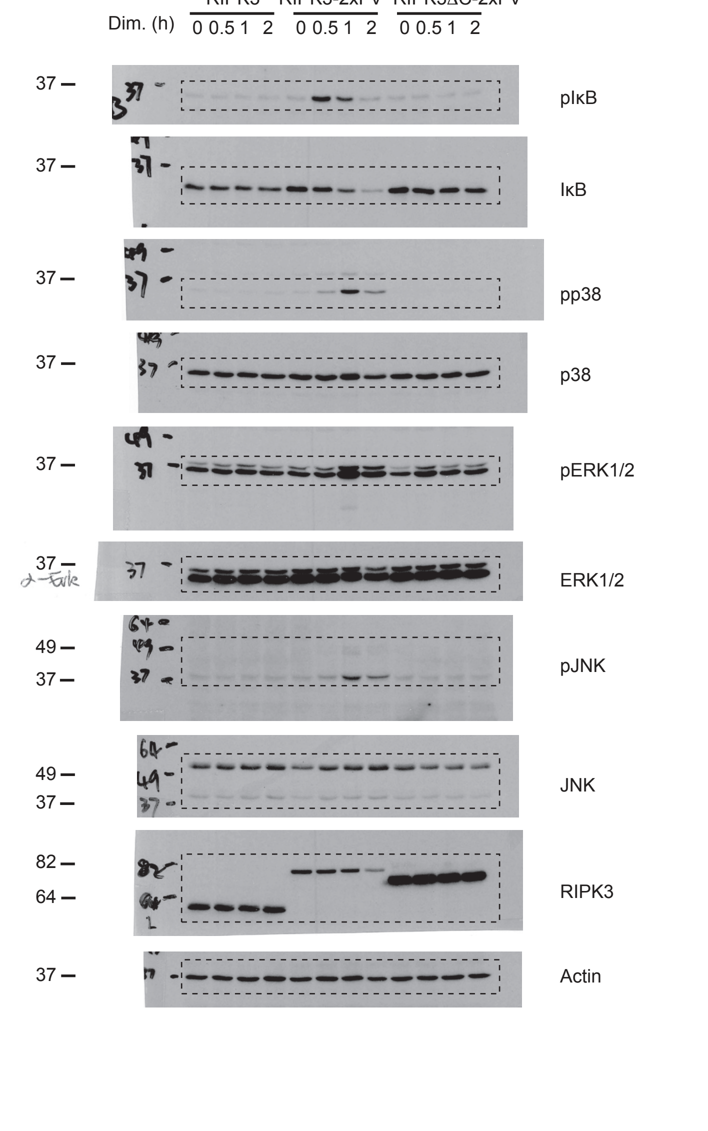
H
U2OS / NOD2
RIPK3
RIPK3-2xFV
RIPK3ΔC-2xFV
Dim. (h) 0 0.5 1 2 0 0.5 1 2 0 0.5 1 2
37
pIκB
37
IκB
37
pp38
37
p38
37
pERK1/2
37
ERK1/2
49
37
pJNK
49
37
JNK
82
64
RIPK3
37
Actin
